# Supplementary material for: The eutherian-specific histone H3.4 promotes germ cell development and reproductive fitness
Source: Nat Commun. 2026 Jun 5;17:7212. doi: 10.1038/s41467-026-73954-9 (PMC13396413; doi:10.1038/s41467-026-73954-9)
Supplement: Supplementary file 1 — Supplementary Information [file 41467_2026_73954_MOESM1_ESM.pdf]

The eutherian-specific histone H3.4 promotes germ cell development and reproductive fitness

Pavel A. Komarov, Philipp Bammer, Ching-Yeu Liang, Hans-Rudolf Hotz, Grigorios Fanourgakis, Sunwoo Chun, Hubertus Kohler, Tim-Oliver Buchholz, Jean-Francois Spetz and Antoine H.F.M Peters

Supplementary Figures

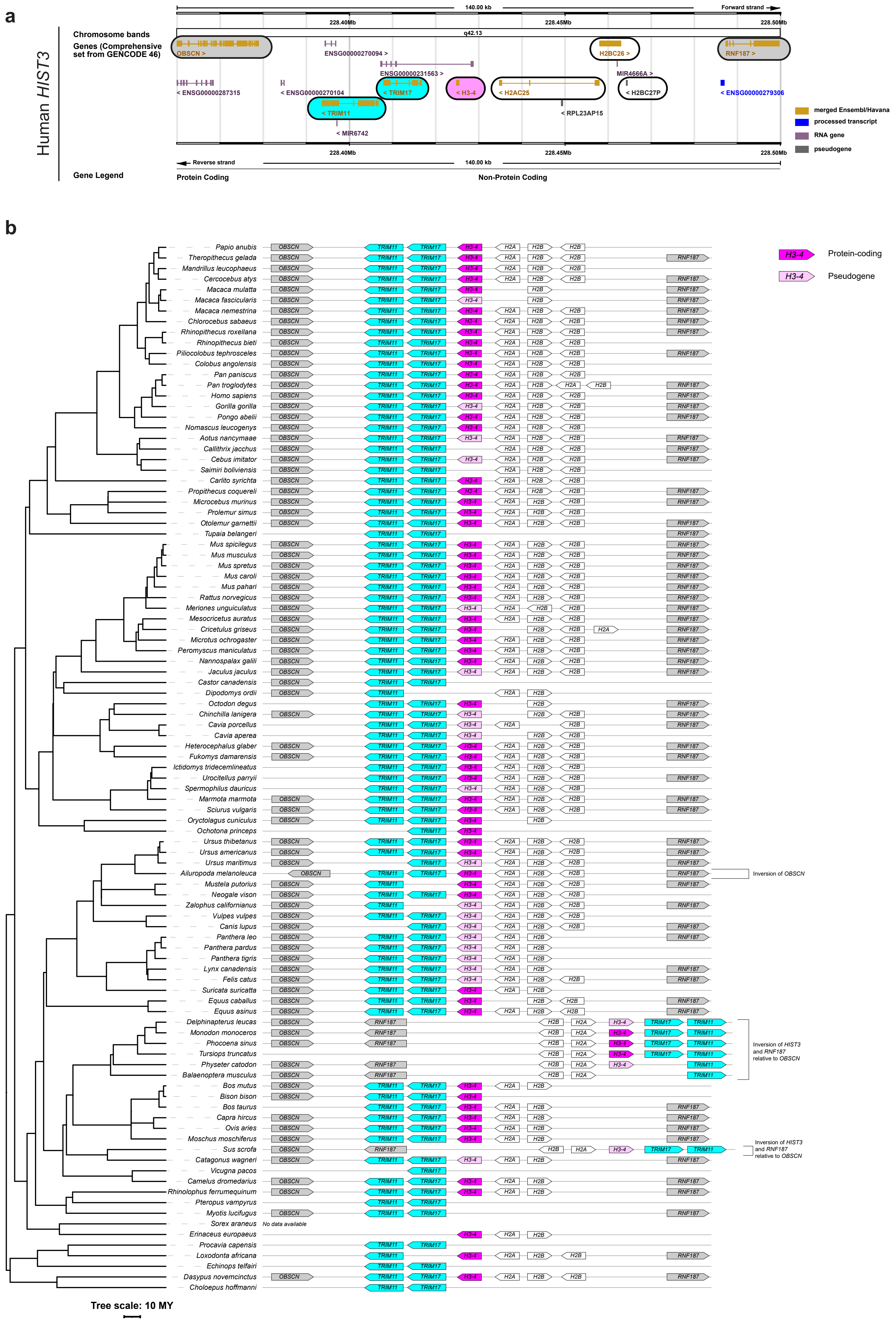
a
Human HIST3
Chromosome bands
Genes (Comprehensive set from GENCODE 46)
Gene Legend
140.00 kb
Forward strand
Reverse strand
228.40Mb
228.45Mb
228.50Mb
q42.13
OBSCN >
ENSG00000270094 >
ENSG00000231563 >
H2BC26 >
RNF187 >
MIR4666A >
< ENSG00000287315
< ENSG00000270104
< TRIM17
< H3-4
< H2AC25
< H2BC27P
< ENSG00000279306
< TRIM11
< RPL23AP15
< MIR6742
merged Ensembl/Havana
processed transcript
RNA gene
pseudogene
Protein Coding
Non-Protein Coding
b
H3-4 Protein-coding
H3-4 Pseudogene
Papio anubis
Theropithecus gelada
Mandrillus leucophaeus
Cercocebus atys
Macaca mulatta
Macaca fascicularis
Macaca nemestrina
Chlorocebus sabaeus
Rhinopithecus roxellana
Rhinopithecus bieti
Piliocolobus tephrosceles
Colobus angolensis
Pan paniscus
Pan troglodytes
Homo sapiens
Gorilla gorilla
Pongo abelii
Nomascus leucogenys
Aotus nancymaae
Callithrix jacchus
Cebus imitator
Saimiri boliviensis
Carlito syrichta
Propithecus coquereli
Microcebus murinus
Prolemur simus
Otolemur garnettii
Tupaia belangeri
Mus spicilegus
Mus musculus
Mus spretus
Mus caroli
Mus pahari
Rattus norvegicus
Meriones unguiculatus
Mesocricetus auratus
Cricetulus griseus
Microtus ochrogaster
Peromyscus maniculatus
Nannospalax galili
Jaculus jaculus
Castor canadensis
Dipodomys ordii
Octodon degus
Chinchilla lanigera
Cavia porcellus
Cavia aperea
Heterocephalus glaber
Fukomys damarensis
Ictidomys tridecemlineatus
Urocitellus parryii
Spermophilus dauricus
Marmota marmota
Sciurus vulgaris
Oryctolagus cuniculus
Ochotona princeps
Ursus thibetanus
Ursus americanus
Ursus maritimus
Ailuropoda melanoleuca
Mustela putorius
Neogale vison
Zalophus californianus
Vulpes vulpes
Canis lupus
Panthera leo
Panthera pardus
Panthera tigris
Lynx canadensis
Felis catus
Suricata suricatta
Equus caballus
Equus asinus
Delphinapterus leucas
Monodon monoceros
Phocoena sinus
Tursiops truncatus
Physeter catodon
Balaenoptera musculus
Bos mutus
Bison bison
Bos taurus
Capra hircus
Ovis aries
Moschus moschiferus
Sus scrofa
Catagonus wagneri
Vicugna pacos
Camelus dromedarius
Rhinolophus ferrumequinum
Pteropus vampyrus
Myotis lucifugus
Sorex araneus
No data available
Erinaceus europaeus
Procavia capensis
Loxodonta africana
Echinops telfairi
Dasypus novemcinctus
Choloepus hoffmanni
OBSCN
TRIM11
TRIM17
H3-4
H2A
H2B
RNF187
Inversion of OBSCN
Inversion of HIST3 and RNF187 relative to OBSCN
Tree scale: 10 MY

Supplementary Figure 1. Conservation of *H3.4*-containing gene clusters in selected mammalian species

a, Ensembl genome browser snapshots of the *H3-4* locus and neighboring genes in the human genome (Release 112). The link to human genome browser view is: https://may2024.archive.ensembl.org/Homo_sapiens/Location/View?db=core;r=1:228360000-228500000

b, Organization of *HIST3 - TRIM* clusters in eutherian species based on genome data in ENSEMBL (release 112) and listed in Supplementary Data 1. The order of species was determined by TimeTree (https://timetree.org). *TRIM17* and *TRIM11* are displayed in cyan. *H3-4* protein-coding orthologs and pseudogenes are presented in purple and light-purple, respectively. Neighboring *H2A* and *H2B* genes are in white and flanking *OBSCN* and *RNF187* genes are shown in gray. Additional non-histone genes, if present within or outside the cluster, are not displayed. If a gene was not annotated with a name but only e.g. with an ENSEMBL ID, we performed a BLASTP search to determine its identity and to define its name. The coordinates of *H3-4* orthologs and details on sequences in different species are available in Supplementary Data 1. For species where we were unable to find *H3-4* orthologs, we included the configuration of *TRIM*, other histone and flanking genes. We used the same color annotation for the different genes as shown in Fig. 1b.

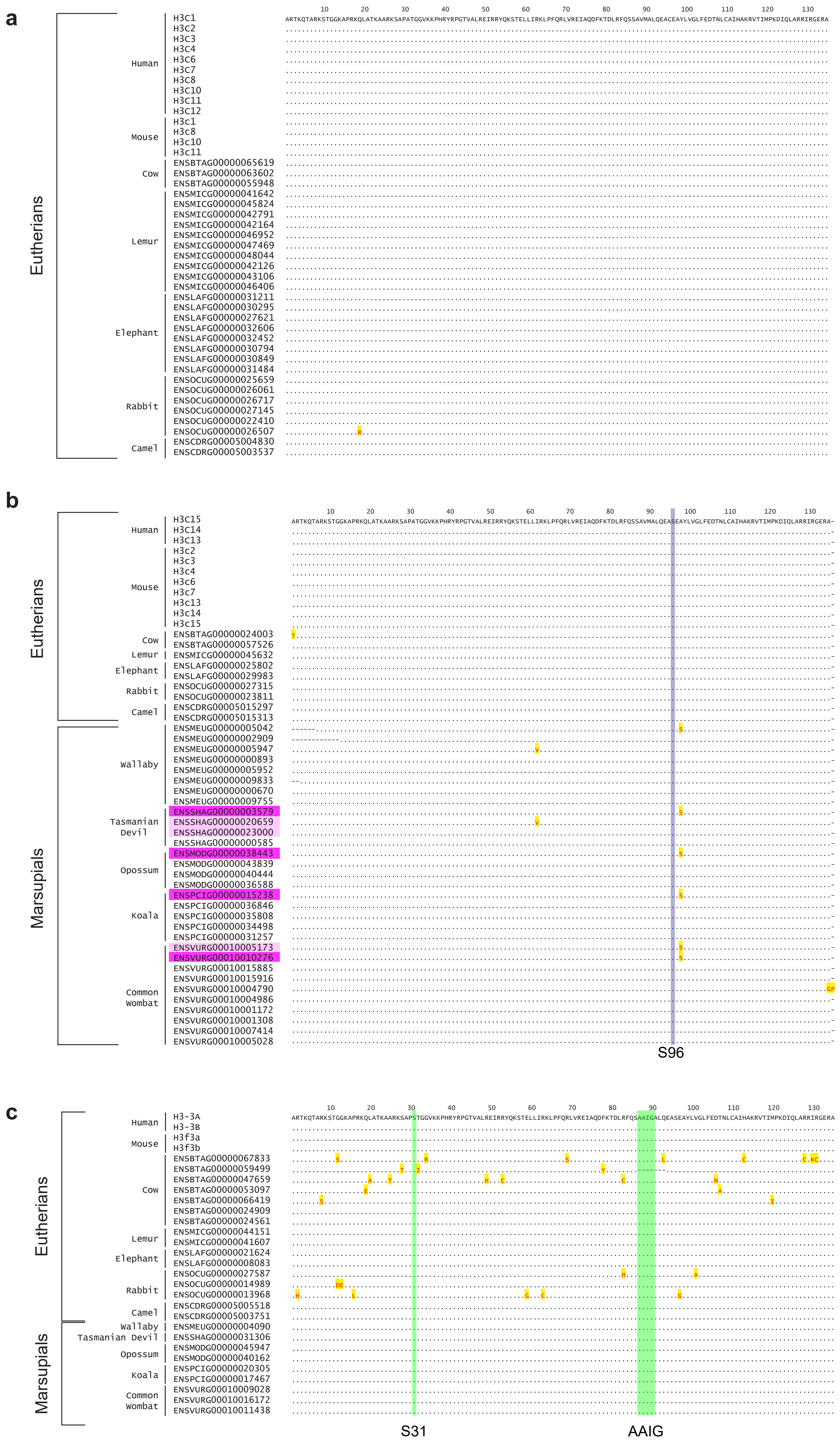
a
Eutherians
Human
H3C1
H3C2
H3C3
H3C4
H3C6
H3C7
H3C8
H3C10
H3C11
H3C12
Mouse
H3c1
H3c8
H3c10
H3c11
Cow
ENSBTAG00000065619
ENSBTAG00000063602
ENSBTAG00000055948
Lemur
ENSMICG00000041642
ENSMICG00000045824
ENSMICG00000042791
ENSMICG00000042164
ENSMICG00000046952
ENSMICG00000047469
ENSMICG00000048044
ENSMICG00000042126
ENSMICG00000043106
ENSMICG00000046406
Elephant
ENSLAFG00000031211
ENSLAFG00000030295
ENSLAFG00000027621
ENSLAFG00000032606
ENSLAFG00000032452
ENSLAFG00000030794
ENSLAFG00000030849
ENSLAFG00000031484
Rabbit
ENSOCUG00000025659
ENSOCUG00000026061
ENSOCUG00000026717
ENSOCUG00000027145
ENSOCUG00000022410
ENSOCUG00000026507
Camel
ENSCDRG00005004830
ENSCDRG00005003537
10 20 30 40 50 60 70 80 90 100 110 120 130
ARTKQTARKSTGGKAPRKQLATKAARKSAPATGGVKKPHRYRPGTVALREIRRYQKSTELLIRKLPFQRLVREIAQDFKTDLRFQSSAVMALQEACEAYLVGLFEDTNLCAIHAKRVTIMPKDIQLARRIRGERA
b
Eutherians
Human
H3C15
H3C14
H3C13
Mouse
H3c2
H3c3
H3c4
H3c6
H3c7
H3c13
H3c14
H3c15
Cow
ENSBTAG00000024003
ENSBTAG00000057526
Lemur
ENSMICG00000045632
Elephant
ENSLAFG00000025802
ENSLAFG00000029983
Rabbit
ENSOCUG00000027315
ENSOCUG00000023811
Camel
ENSCDRG00005015297
ENSCDRG00005015313
Marsupials
Wallaby
ENSMEUG00000005042
ENSMEUG00000002909
ENSMEUG00000005947
ENSMEUG00000000893
ENSMEUG00000005952
ENSMEUG00000009833
ENSMEUG00000000670
ENSMEUG00000009755
Tasmanian Devil
ENSSHAG00000003579
ENSSHAG00000020659
ENSSHAG00000023000
ENSSHAG00000000585
Opossum
ENSMODG00000038443
ENSMODG00000043839
ENSMODG00000040444
ENSMODG00000036588
Koala
ENSPCIG00000015238
ENSPCIG00000036846
ENSPCIG00000035808
ENSPCIG00000034498
ENSPCIG00000031257
Common Wombat
ENSVURG00010005173
ENSVURG00010010276
ENSVURG00010015885
ENSVURG00010015916
ENSVURG00010004790
ENSVURG00010004986
ENSVURG00010001172
ENSVURG00010001308
ENSVURG00010007414
ENSVURG00010005028
10 20 30 40 50 60 70 80 90 100 110 120 130
ARTKQTARKSTGGKAPRKQLATKAARKSAPATGGVKKPHRYRPGTVALREIRRYQKSTELLIRKLPFQRLVREIAQDFKTDLRFQSSAVMALQEASEAYLVGLFEDTNLCAIHAKRVTIMPKDIQLARRIRGERA-
S96
c
Eutherians
Human
H3-3A
H3-3B
Mouse
H3f3a
H3f3b
Cow
ENSBTAG00000067833
ENSBTAG00000059499
ENSBTAG00000047659
ENSBTAG00000053097
ENSBTAG00000066419
ENSBTAG00000024909
ENSBTAG00000024561
Lemur
ENSMICG00000044151
ENSMICG00000041607
Elephant
ENSLAFG00000021624
ENSLAFG00000008083
Rabbit
ENSOCUG00000027587
ENSOCUG00000014989
ENSOCUG00000013968
Camel
ENSCDRG00005005518
ENSCDRG00005003751
Marsupials
Wallaby
ENSMEUG00000004090
Tasmanian Devil
ENSSHAG00000031306
Opossum
ENSMODG00000045947
ENSMODG00000040162
Koala
ENSPCIG00000020305
ENSPCIG00000017467
Common Wombat
ENSVURG00010009028
ENSVURG00010016172
ENSVURG00010011438
10 20 30 40 50 60 70 80 90 100 110 120 130
ARTKQTARKSTGGKAPRKQLATKAARKSAPSTGGVKKPHRYRPGTVALREIRRYQKSTELLIRKLPFQRLVREIAQDFKTDLRFQSAAIGALQEASEAYLVGLFEDTNLCAIHAKRVTIMPKDIQLARRIRGERA
S31
AAIG

Supplementary Figure 2. Protein sequences of H3.1, H3.2 and H3.3 in selected placental and marsupial mammals

Amino acid sequence alignments of H3.1 (**a**), H3.2 (**b**) and H3.3 (**c**) proteins in selected placental and marsupial species. Marsupial *H3.2* genes that are putative orthologs of placental *H3-4* and are linked to *TRIM11* genes are highlighted in purple, while those not located adjacent to *TRIM-11* genes are shown in light-purple. For H3.2 proteins, S96 and S98 residues are highlighted in blue and yellow respectively. For H3.3 proteins, S31 and AAIG residues are highlighted in green. Moreover, variant amino acids are highlighted in yellow.

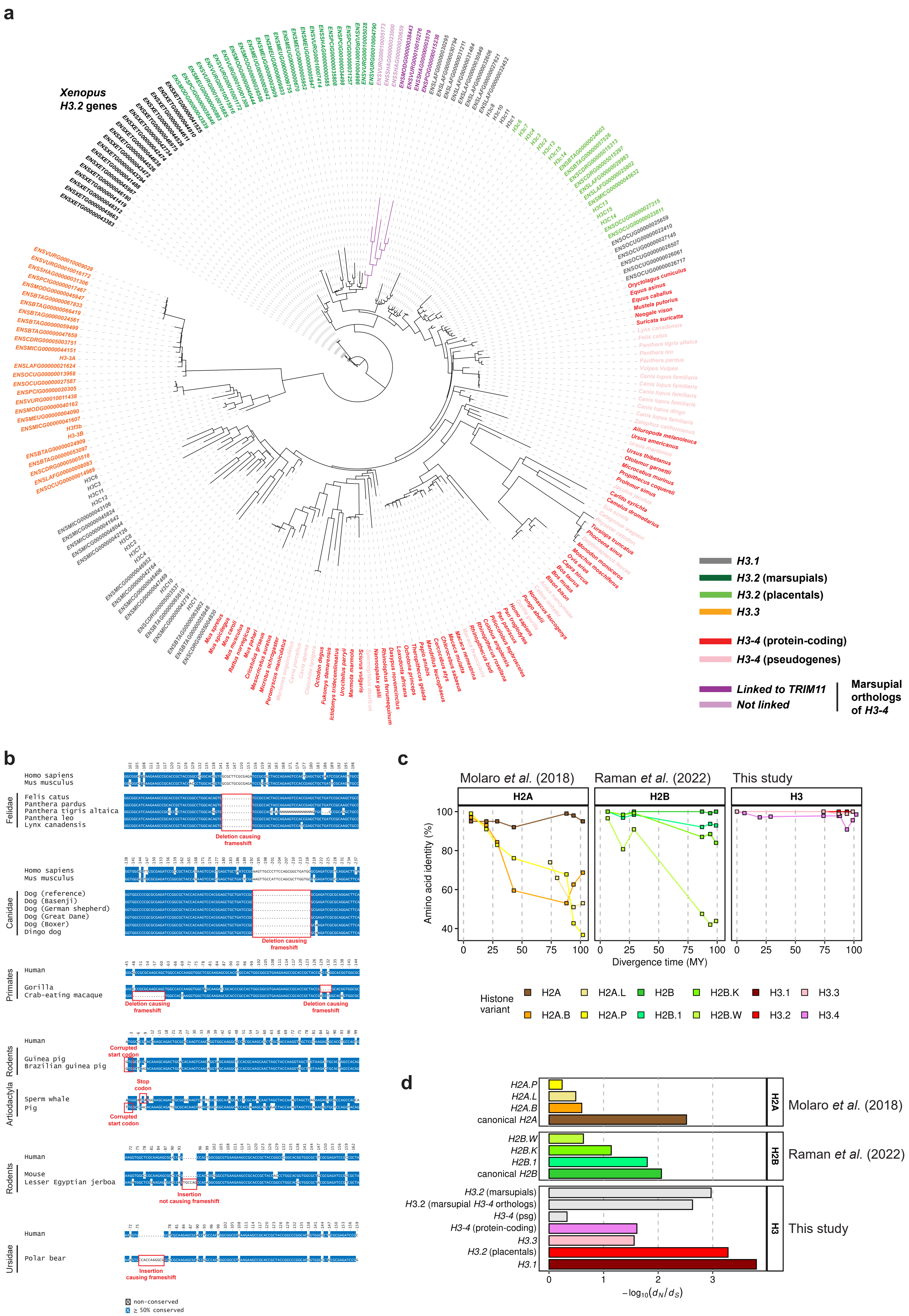
a
Xenopus H3.2 genes
H3.1
H3.2 (marsupials)
H3.2 (placentals)
H3.3
H3-4 (protein-coding)
H3-4 (pseudogenes)
Linked to TRIM11
Not linked
Marsupial orthologs of H3-4
b
Felidae
Canidae
Primates
Rodents
Artiodactyla
Ursidae
Homo sapiens
Mus musculus
Felis catus
Panthera pardus
Panthera tigris altaica
Panthera leo
Lynx canadensis
Deletion causing frameshift
Dog (reference)
Dog (Basenji)
Dog (German shepherd)
Dog (Great Dane)
Dog (Boxer)
Dingo dog
Human
Gorilla
Crab-eating macaque
Guinea pig
Brazilian guinea pig
Corrupted start codon
Stop codon
Sperm whale
Pig
Mouse
Lesser Egyptian jerboa
Insertion not causing frameshift
Polar bear
Insertion causing frameshift
non-conserved
≥ 50% conserved
c
Molaro et al. (2018)
Raman et al. (2022)
This study
H2A
H2B
H3
Amino acid identity (%)
Divergence time (MY)
Histone variant
H2A
H2A.B
H2A.L
H2A.P
H2B
H2B.1
H2B.K
H2B.W
H3.1
H3.2
H3.3
H3.4
d
H2A.P
H2A.L
H2A.B
canonical H2A
H2B.W
H2B.K
H2B.1
canonical H2B
H3.2 (marsupials)
H3.2 (marsupial H3-4 orthologs)
H3-4 (psg)
H3-4 (protein-coding)
H3.3
H3.2 (placentals)
H3.1
$-\log_{10}(d_N/d_S)$

Supplementary Figure 3. Analysis of sequence divergence and evolutionary dynamics of *H3* genes

a, Cladogram showing the results of maximum-likelihood analysis of *H3* genes. For *H3-4* protein-coding genes (red) and pseudogenes (light-red), sequences from all species were used, if available (Supplementary Data 1). The H3.1, H3.2 and H3.3-encoding genes (gray, green and orange, respectively) were retrieved from the selected species (Supplementary Data 2). Marsupial *H3.2* genes that are putative orthologs of placental *H3-4* and are linked to *TRIM11* genes are highlighted in purple, while those not located adjacent to *TRIM11* genes are shown in light-purple.

b, Examples of truncations, insertions and nonsense mutations of DNA sequences of *H3-4* pseudogenes in different species. Human *H3-4* and mouse *H3f4* sequences are given as references on the top.

c, Analysis of protein sequence variation of H3 variants relative to human. As comparative references, variation rates of several H2A and H2B variants were added from published studies[7,8]. Details of this analysis are available in Supplementary Data 3.

d, Phylogenetic Analysis Using Maximum Likelihood (PAML) of *H3* genes showing ratios of nonsynonymous to synonymous mutation rates (d_n/d_s) ($-log_{10}$-transformed). Separate analyses were done for *H3.1*, *H3.2* and *H3.3* genes in selected species, as well as for protein-coding *H3-4* orthologs and their pseudogenes. More detailed results of statistical testing are provided in Supplementary Data 4. As comparative references, d_n/d_s values of several H2A- and H2B-encoding genes were added from published studies[7,8]. Color coding of genes is the same as in panel c.

Locations of *H3* genes in opossum

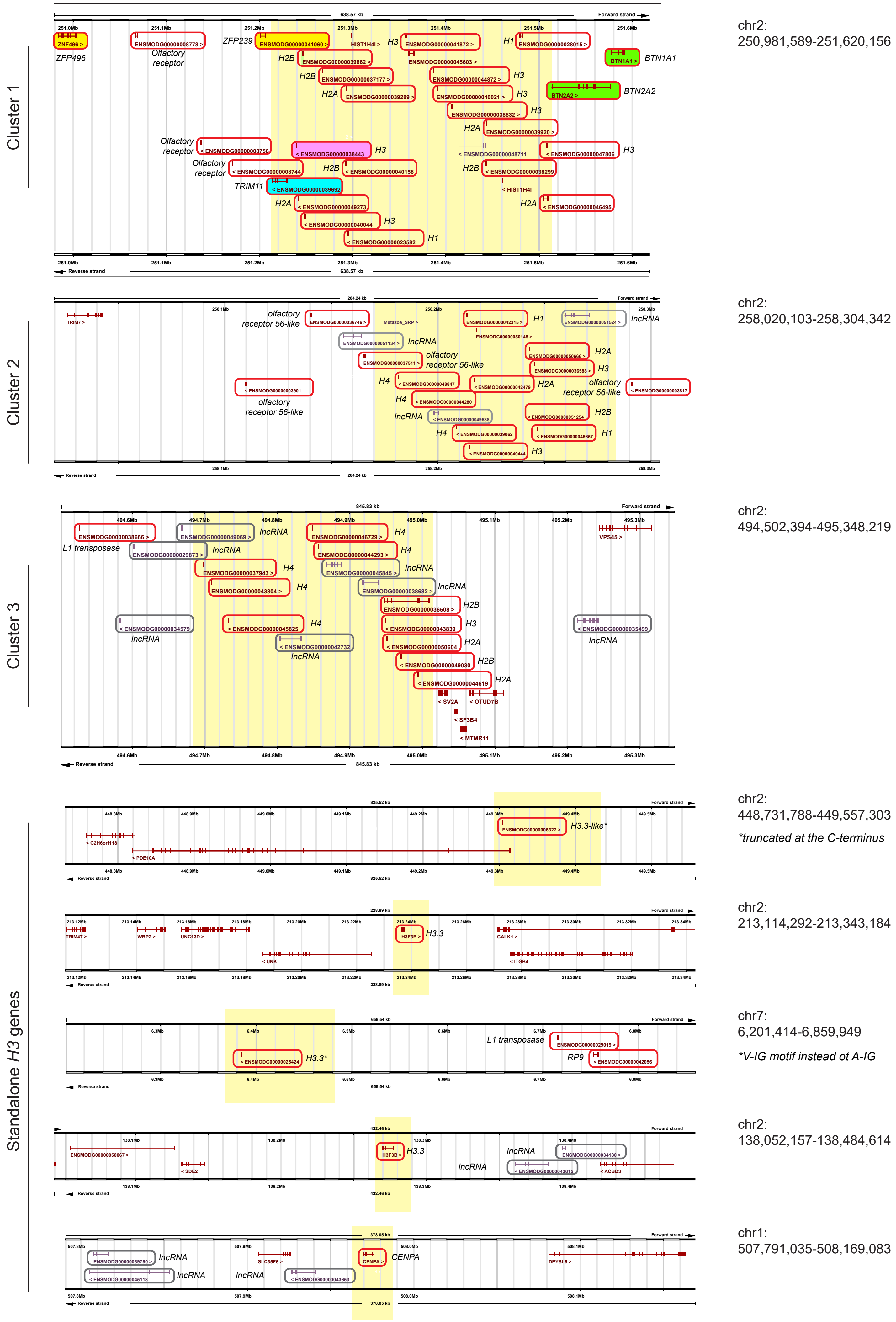

Supplementary Figure 4. Locations of *H3* genes in opossum

The opossum genome (ENSEMBL Release 112) harbors three large histone clusters with replication-dependent *H2A*, *H2B*, *H3* and *H4* genes as well as four *H3.3* loci and one *CENPA* locus. In the Ensembl genome browser snapshots of cluster 1 (on chr. 2: 250,981,589 - 251,620,156), *TRIM11* and *H3.2* (the putative *H3-4* ortholog) are highlighted in cyan and purple, respectively. Neighboring *ZFP239* and *ZFP496* are yellow and *BTN2A2* and *BTN1A1* are green. For each location, the respective coordinates are shown on the right.

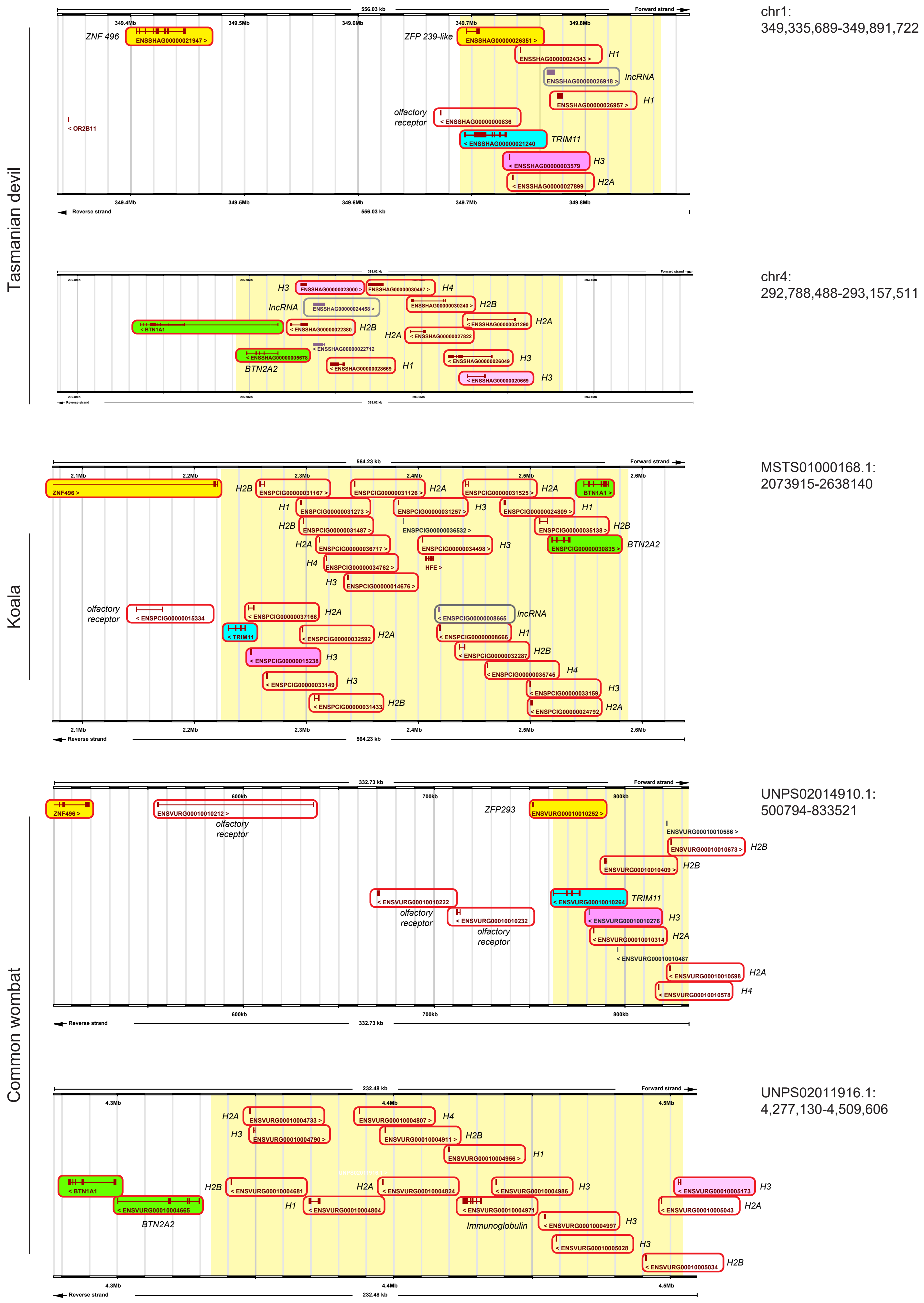
Tasmanian devil
chr1:
349,335,689-349,891,722
556.03 kb
Forward strand
349.4Mb
349.5Mb
349.6Mb
349.7Mb
349.8Mb
ZNF 496
ENSSHAG00000021947 >
ZFP 239-like
ENSSHAG00000026351 >
ENSSHAG00000024343 >
H1
ENSSHAG00000026918 >
lncRNA
ENSSHAG00000026957 >
H1
< OR2B11
olfactory receptor
< ENSSHAG00000000836
< ENSSHAG00000021240
TRIM11
< ENSSHAG00000003579
H3
< ENSSHAG00000027899
H2A
Reverse strand
chr4:
292,788,488-293,157,511
H3
ENSSHAG00000023000 >
ENSSHAG00000030497 >
H4
lncRNA
ENSSHAG00000024458 >
ENSSHAG00000030240 >
H2B
< ENSSHAG00000031290
H2A
< BTN1A1
< ENSSHAG00000022380
H2B
H2A
< ENSSHAG00000027822
< ENSSHAG00000022712
< ENSSHAG00000005678
BTN2A2
< ENSSHAG00000028669
H1
< ENSSHAG00000026049
H3
< ENSSHAG00000020659
H3
Koala
MSTS01000168.1:
2073915-2638140
564.23 kb
2.1Mb
2.2Mb
2.3Mb
2.4Mb
2.5Mb
2.6Mb
ZNF496 >
H2B
ENSPCIG00000031167 >
ENSPCIG00000031126 >
H2A
ENSPCIG00000031525 >
H2A
BTN1A1 >
H1
ENSPCIG00000031273 >
ENSPCIG00000031257 >
H3
ENSPCIG00000024809 >
H1
H2B
ENSPCIG00000031487 >
ENSPCIG00000036532 >
ENSPCIG00000035138 >
H2B
H2A
ENSPCIG00000036717 >
ENSPCIG00000034498 >
H3
ENSPCIG00000030835 >
BTN2A2
H4
ENSPCIG00000034762 >
HFE >
H3
ENSPCIG00000014676 >
olfactory receptor
< ENSPCIG00000015334
< ENSPCIG00000037166
H2A
< ENSPCIG00000008665
lncRNA
< TRIM11
< ENSPCIG00000032592
H2A
< ENSPCIG00000008666
H1
< ENSPCIG00000032287
H2B
< ENSPCIG00000015238
H3
< ENSPCIG00000035745
H4
< ENSPCIG00000033149
H3
< ENSPCIG00000033159
H3
< ENSPCIG00000031433
H2B
< ENSPCIG00000024792
H2A
Common wombat
UNPS02014910.1:
500794-833521
332.73 kb
600kb
700kb
800kb
ZNF496 >
ENSVURG00010010212 >
olfactory receptor
ZFP293
ENSVURG00010010252 >
ENSVURG00010010586 >
ENSVURG00010010673 >
H2B
ENSVURG00010010409 >
H2B
< ENSVURG00010010222
olfactory receptor
< ENSVURG00010010232
olfactory receptor
< ENSVURG00010010264
TRIM11
< ENSVURG00010010276
H3
< ENSVURG00010010314
H2A
< ENSVURG00010010487
< ENSVURG00010010598
H2A
< ENSVURG00010010578
H4
UNPS02011916.1:
4,277,130-4,509,606
232.48 kb
4.3Mb
4.4Mb
4.5Mb
H2A
ENSVURG00010004733 >
ENSVURG00010004807 >
H4
H3
ENSVURG00010004790 >
ENSVURG00010004911 >
H2B
ENSVURG00010004956 >
H1
< BTN1A1
H2B
< ENSVURG00010004681
H2A
< ENSVURG00010004824
< ENSVURG00010004986
H3
< ENSVURG00010005173
H3
< ENSVURG00010004665
H1
< ENSVURG00010004804
< ENSVURG00010004971
< ENSVURG00010005043
H2A
BTN2A2
Immunoglobulin
< ENSVURG00010004997
H3
< ENSVURG00010005028
H3
< ENSVURG00010005034
H2B

Supplementary Figure 5. Genomic locations of putative *H3-4* orthologs in Tasmanian devil, Koala and Common wombat

Ensembl genome browser snapshots of histone clusters harboring *H3.2* genes that are putative *H3-4* orthologs in Tasmanian devil, Koala, and Common wombat. The color annotation of genes is identical to the one shown in Supplementary Fig. 4. *H3.2* genes with sequence similarity to *H3.2* genes that are putative *H3-4* orthologs but that are not located next to *TRIM11* genes are displayed in light-purple. For each location, the respective coordinates are shown on the right.

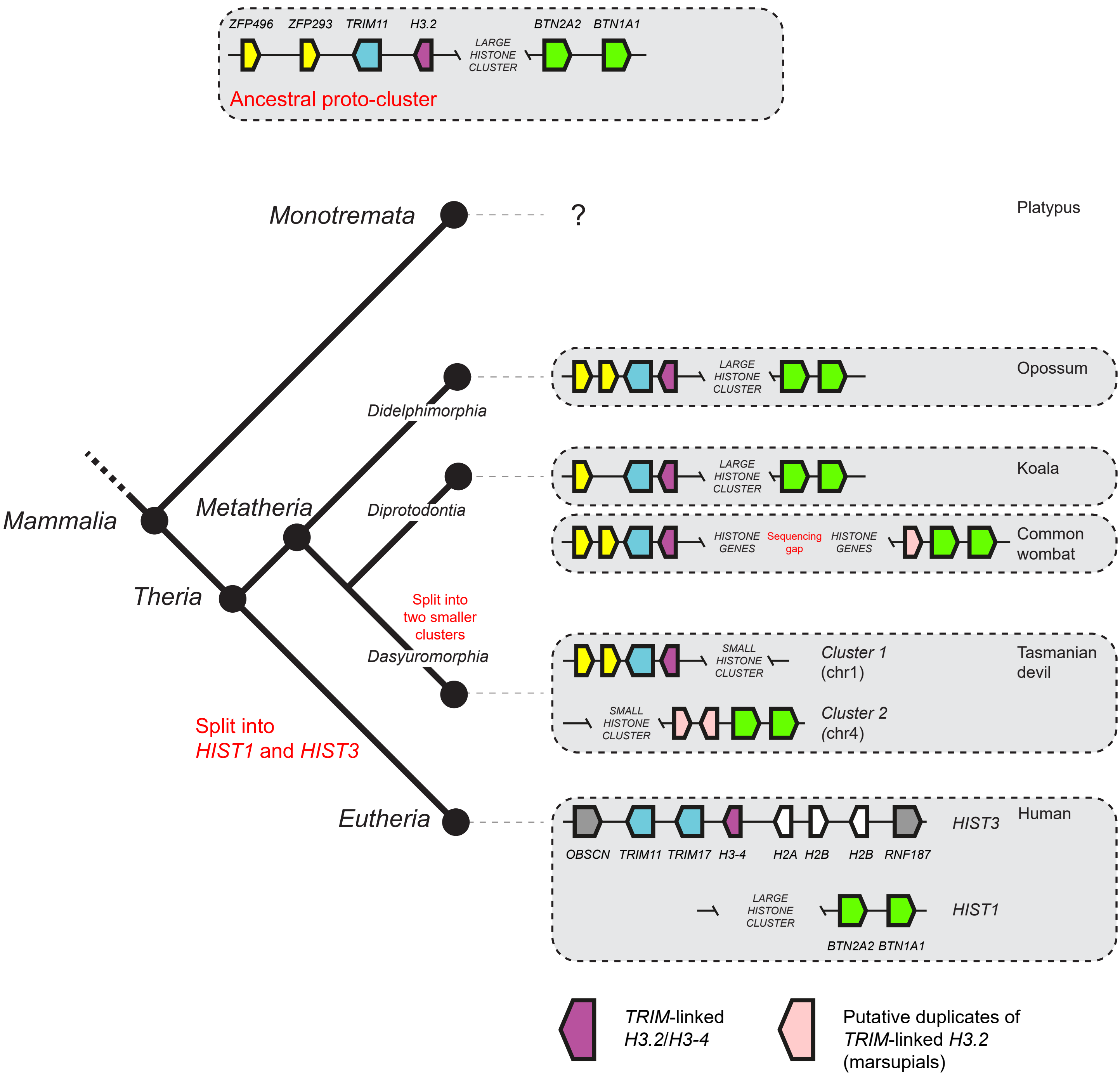
ZFP496
ZFP293
TRIM11
H3.2
LARGE HISTONE CLUSTER
BTN2A2
BTN1A1
Ancestral proto-cluster
Monotremata
?
Platypus
Didelphimorphia
Opossum
Diprotodontia
Koala
HISTONE GENES
Sequencing gap
Common wombat
Mammalia
Metatheria
Theria
Split into two smaller clusters
Dasyuromorphia
SMALL HISTONE CLUSTER
Cluster 1 (chr1)
Cluster 2 (chr4)
Tasmanian devil
Split into HIST1 and HIST3
Eutheria
OBSCN
TRIM17
H3-4
H2A
H2B
RNF187
HIST3
HIST1
Human
TRIM-linked H3.2/H3-4
Putative duplicates of TRIM-linked H3.2 (marsupials)

Supplementary Figure 6. Evolution of the ancestral *TRIM11*-linked histone cluster and its derivatives in marsupials and eutherian species

Top: composition of an ancestral histone protocluster proposed to exist in the common ancestor of marsupials and eutherians. Schematic views of histone cluster derivatives existing in four marsupial species belonging to the Monotremata and Metatheria lineages and in Eutherians (human) are indicated below. In Common wombat, due to missing sequencing information, we were unable to establish whether the shown histone cluster is intact or split in two. In Tasmanian devil, two smaller histone clusters were detected on chr1 and chr4. In Wallaby, we could not obtain data due to poor assembly. Cladogram structure is inferred from TimeTree (https://timetree.org), nodes and branches are not scaled.

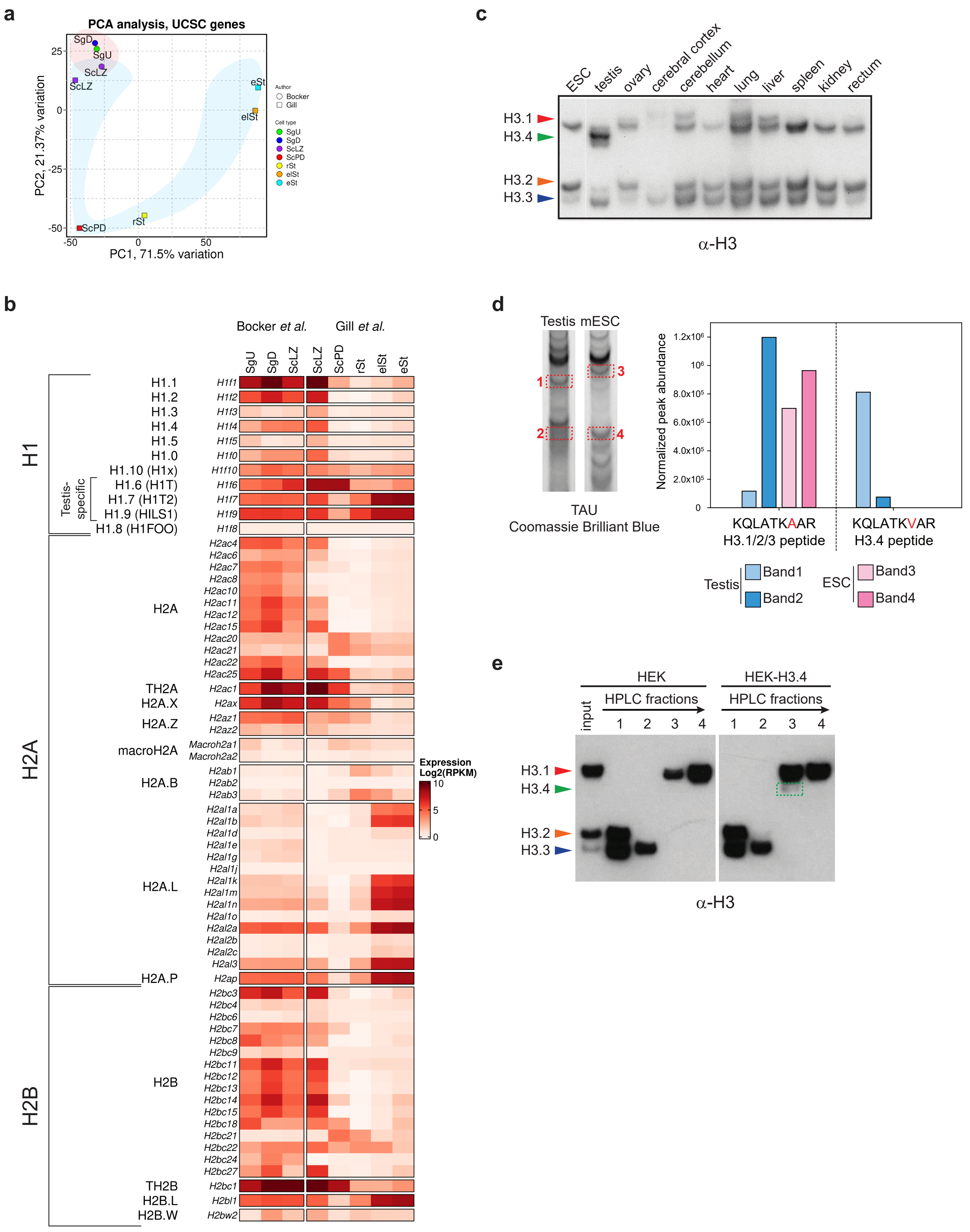

a
PCA analysis, UCSC genes
PC2, 21.37% variation
PC1, 71.5% variation
SgD
SgU
ScLZ
ScLZ
eSt
elSt
rSt
ScPD
Author
Bocker
Gill
Cell type
SgU
SgD
ScLZ
ScPD
rSt
elSt
eSt
c
ESC
testis
ovary
cerebral cortex
cerebellum
heart
lung
liver
spleen
kidney
rectum
H3.1
H3.4
H3.2
H3.3
α-H3
b
Bocker et al.
Gill et al.
SgU
SgD
ScLZ
ScLZ
ScPD
rSt
elSt
eSt
H1
H1.1 H1f1
H1.2 H1f2
H1.3 H1f3
H1.4 H1f4
H1.5 H1f5
H1.0 H1f0
H1.10 (H1x) H1f10
Testis-specific
H1.6 (H1T) H1f6
H1.7 (H1T2) H1f7
H1.9 (HILS1) H1f9
H1.8 (H1FOO) H1f8
H2A
H2A
H2ac4
H2ac6
H2ac7
H2ac8
H2ac10
H2ac11
H2ac12
H2ac15
H2ac20
H2ac21
H2ac22
H2ac25
TH2A H2ac1
H2A.X H2ax
H2A.Z H2az1 H2az2
macroH2A Macroh2a1 Macroh2a2
H2A.B H2ab1 H2ab2 H2ab3
H2A.L
H2al1a
H2al1b
H2al1d
H2al1e
H2al1g
H2al1j
H2al1k
H2al1m
H2al1n
H2al1o
H2al2a
H2al2b
H2al2c
H2al3
H2A.P H2ap
H2B
H2B
H2bc3
H2bc4
H2bc6
H2bc7
H2bc8
H2bc9
H2bc11
H2bc12
H2bc13
H2bc14
H2bc15
H2bc18
H2bc21
H2bc22
H2bc24
H2bc27
TH2B H2bc1
H2B.L H2bl1
H2B.W H2bw2
Expression
Log2(RPKM)
10
5
0
d
Testis mESC
1
2
3
4
TAU
Coomassie Brilliant Blue
Normalized peak abundance
1.2x10⁶
10⁶
8.0x10⁵
6.0x10⁵
4.0x10⁵
2.0x10⁵
0
KQLATKAAR
H3.1/2/3 peptide
KQLATKVAR
H3.4 peptide
Testis
Band1
Band2
ESC
Band3
Band4
e
HEK
HEK-H3.4
HPLC fractions
HPLC fractions
input
1
2
3
4
1
2
3
4
H3.1
H3.4
H3.2
H3.3
α-H3

Supplementary Figure 7. The expression of H3 variants in mouse tissues

a, Principal component analysis (PCA) comparing RNA-seq expression of UCSC-annotated genes in different cell types[57,58]. The RNA-seq libraries of the two independent studies recapitulate the respective developmental trajectories during spermatogenesis.

b, Heatmap showing mRNA expression H1, H2A and H2B encoding genes during spermatogenesis. The names of histone genes are according to the current nomenclature[34]. Abbreviations: SgU, SgD, ScLZ, ScPD, rSt, elSt and eSt refer to populations of FACS-sorted undifferentiated and differentiating spermatogonia, leptotene/zygotene and pachytene/diplotene stage spermatocytes and round, elongating and elongated spermatids.

c, Western blot showing H3.1, H3.4, H3.2 and H3.3 proteins separated by TAU gel electrophoresis in extracts of mouse embryonic stem cells and various tissues isolated from adult mice. A panH3 antibody recognizing the C-terminus of H3 as used to detect all H3 variants.

d, Mass spectrometry analysis of histones isolated from testes and mESCs. We separated histones by TAU gel electrophoresis and stained them with Coomassie Brilliant Blue. We excised histone protein containing bands and subjected them to mass spectrometry. We quantified normalized peak abundances corresponding to the H3.1/H3.2/H3.3 (KQLATKA$_{24}$AR, left) common peptide and the H3.4 (KQLATV$_{24}$AR, right) specific peptide in the four excised bands. The diagnostic amino acids of the two peptides are labeled in red. Band 1 contains: H3.4, band 2: H3.3, band 3: H3.1 and band 4: H3.3.

e, Proteins blots showing the migration characteristics of H3.4 relative to other H3 proteins separated by TAU gel electrophoresis. We prepared histone extracts from control HEK293 cells or HEK293 cells expressing H3.4. We obtained different H3 protein containing fractions by reverse phase HPLC separation and subjected them to TAU gel electrophoresis. Fraction 3 contains the H3.4 protein.

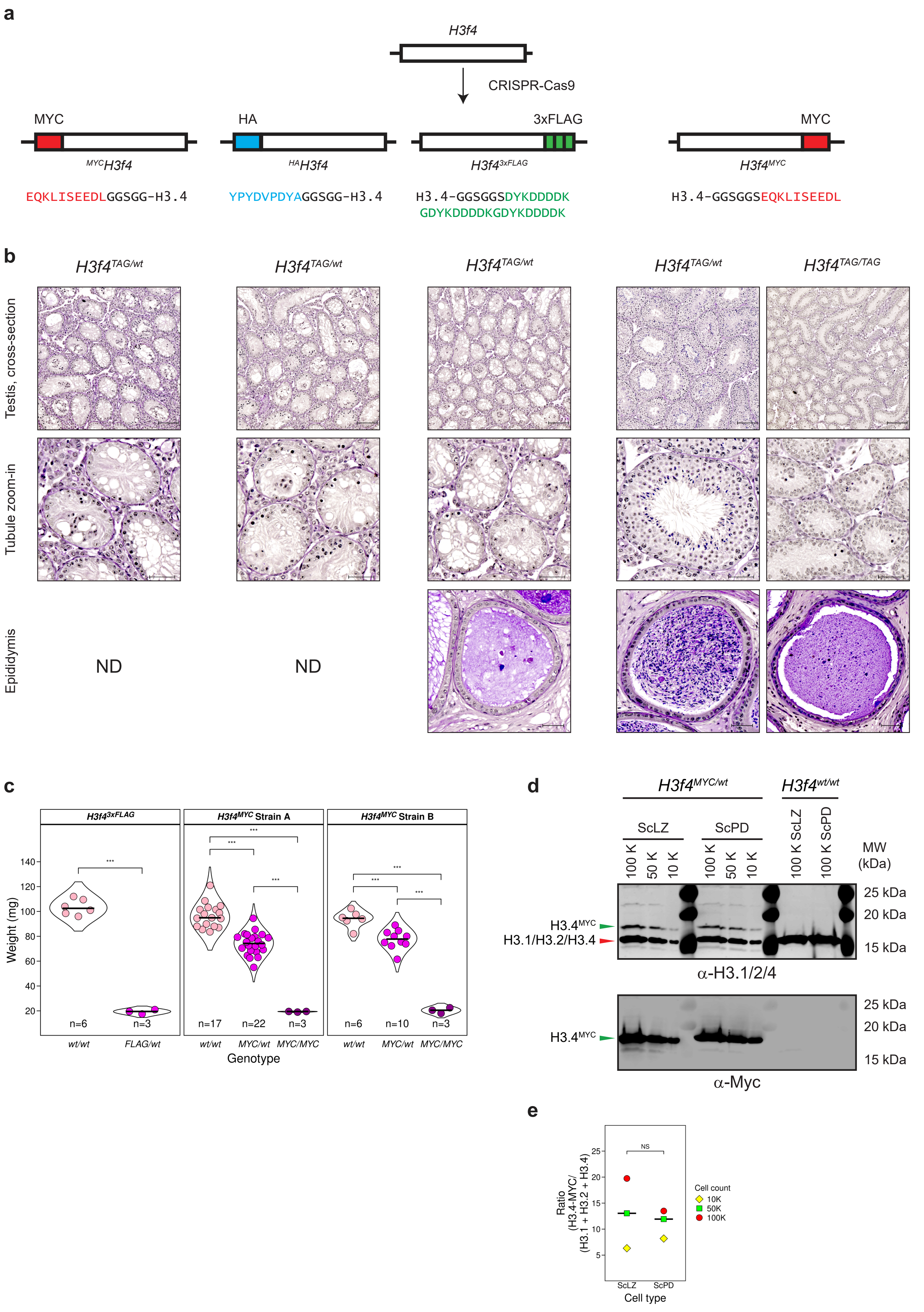
a
H3f4
CRISPR-Cas9
MYC
HA
3xFLAG
MYC
MYCH3f4
HAH3f4
H3f4 3xFLAG
H3f4 MYC
EQKLISEEDLGGSGG-H3.4
YPYDVPDYAGGSGG-H3.4
H3.4-GGSGGSDYKDDDDK
GDYKDDDDKGDYKDDDDK
H3.4-GGSGGSEQKLISEEDL
b
H3f4 TAG/wt
H3f4 TAG/wt
H3f4 TAG/wt
H3f4 TAG/wt
H3f4 TAG/TAG
Testis, cross-section
Tubule zoom-in
Epididymis
ND
ND
c
H3f4 3xFLAG
H3f4 MYC Strain A
H3f4 MYC Strain B
Weight (mg)
140
120
100
80
60
40
20
***
n=6
n=3
n=17
n=22
n=3
n=6
n=10
n=3
wt/wt
FLAG/wt
wt/wt
MYC/wt
MYC/MYC
wt/wt
MYC/wt
MYC/MYC
Genotype
d
H3f4 MYC/wt
H3f4 wt/wt
ScLZ
ScPD
100 K
50 K
10 K
100 K
50 K
10 K
100 K ScLZ
100 K ScPD
MW (kDa)
25 kDa
20 kDa
15 kDa
H3.4 MYC
H3.1/H3.2/H3.4
α-H3.1/2/4
H3.4 MYC
α-Myc
e
Ratio (H3.4-MYC/ (H3.1 + H3.2 + H3.4)
25
20
15
10
5
NS
Cell count
10K
50K
100K
ScLZ
ScPD
Cell type

Supplementary Figure 8. CRISPR/Cas9 approaches to tag the endogenous *H3f4* gene

a, Schematic overview of four different transgenic mouse lines generated by CRISPR/Cas9-mediated tagging of the endogenous *H3f4* gene. The sequences of protein tags are displayed in different colors and linker sequences in black. Sequences of sgRNAs and repair templates are provided in Supplementary Data 6 and 7, respectively.

b, PAS-haematoxylin-stained histological cross-sections of testis and epididymal tissues obtained from mice bearing tagged *H3f4* genes. Scale bars: 100 µm (testis, cross-section overview) and 50 µm (tubule zoom-in and epididymis). ND: not determined.

c, Violin plots showing testicular weights of control, heterozygous and homozygous transgenic mice of the $H3f4^{3xFLAG}$ and two independent $H3f4^{MYC}$ strains. In the violin plots, each dot represents the mean weight of two testes taken from each mouse. The number (n) of mice per genotype are indicated. Two-sided t-test was performed. When three genotypes were compared, Bonferroni correction was used. *** $P \leq 0.001$.

d, SDS-PAGE Western blot analysis of $H3.4^{MYC}$ expression in FACS-purified ScLZ and ScPD cells of wildtype $H3f4^{wt/wt}$ and heterozygous $H3f4^{MYC/wt}$ mice. Blots were probed with antibodies recognizing H3.1/H3.2/H3.4 (upper panel) and MYC (lower panel) epitopes. Bands corresponding to the tagged $H3.4^{MYC}$ and untagged H3.1, H3.2 and H3.4 proteins are indicated by arrows.

e, ImageJ quantification of bands in panel d. Ratios of $H3.4^{MYC}$ (upper band) relative to the band corresponding to the untagged H3 proteins (lower). Separate data points correspond to lanes with 100K, 50K and 10K cells. Two-sided t-test was performed to compare the values of ScLZ and ScPD bands. NS – not significant.

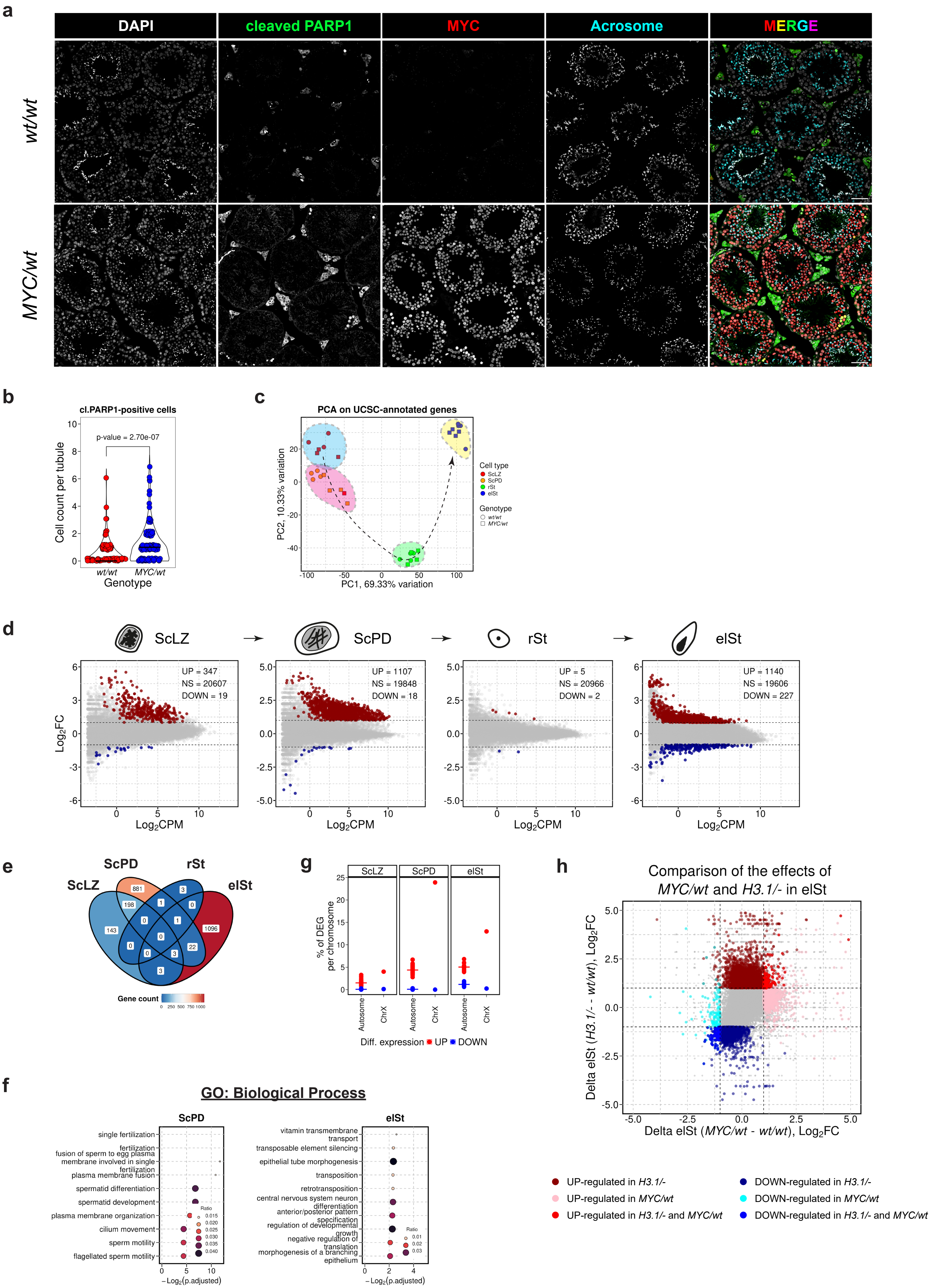
a
DAPI
cleaved PARP1
MYC
Acrosome
MERGE
wt/wt
MYC/wt
b
cl.PARP1-positive cells
p-value = 2.70e-07
Cell count per tubule
wt/wt
MYC/wt
Genotype
c
PCA on UCSC-annotated genes
PC2, 10.33% variation
PC1, 69.33% variation
Cell type
ScLZ
ScPD
rSt
elSt
Genotype
wt/wt
MYC/wt
d
ScLZ
ScPD
rSt
elSt
UP = 347
NS = 20607
DOWN = 19
UP = 1107
NS = 19848
DOWN = 18
UP = 5
NS = 20966
DOWN = 2
UP = 1140
NS = 19606
DOWN = 227
Log2FC
Log2CPM
e
ScPD
rSt
ScLZ
elSt
Gene count
g
ScLZ
ScPD
elSt
% of DEG per chromosome
Autosome
ChrX
Diff. expression UP DOWN
h
Comparison of the effects of MYC/wt and H3.1/- in elSt
Delta elSt (H3.1/- - wt/wt), Log2FC
Delta elSt (MYC/wt - wt/wt), Log2FC
UP-regulated in H3.1/-
UP-regulated in MYC/wt
UP-regulated in H3.1/- and MYC/wt
DOWN-regulated in H3.1/-
DOWN-regulated in MYC/wt
DOWN-regulated in H3.1/- and MYC/wt
f
GO: Biological Process
ScPD
single fertilization
fertilization
fusion of sperm to egg plasma membrane involved in single fertilization
plasma membrane fusion
spermatid differentiation
spermatid development
plasma membrane organization
cilium movement
sperm motility
flagellated sperm motility
Ratio
−Log2(p.adjusted)
elSt
vitamin transmembrane transport
transposable element silencing
epithelial tube morphogenesis
transposition
retrotransposition
central nervous system neuron differentiation
anterior/posterior pattern specification
regulation of developmental growth
negative regulation of translation
morphogenesis of a branching epithelium
Ratio
−Log2(p.adjusted)

Supplementary Figure 9. Cell death and transcriptional mis-regulation in germ cells expressing MYC-tagged H3.4

a, Immunofluorescence images showing cleaved PARP1 and MYC signals in sections of seminiferous tubules of control (*H3f4*$^{wt/wt}$) and heterozygous (*H3f4*$^{MYCt/wt}$) mice. Scale bar: 50 μm.

b, Quantification of cells positive for cleaved PARP1, as displayed in panel a. The number of tubules analyzed: 159 for *H3f4*$^{wt/wt}$ and 154 for *H3f*$^{MYCt/wt}$. *p*-value of paired t-test comparison is displayed.

c, Principal component analysis (PCA) comparing RNA-seq expression of UCSC-annotated genes among of biological replicates and cell types from *H3f4*$^{wt/wt}$ and *H3f*$^{MYCt/wt}$ animals.

d, Scatter plots showing gene expression log2 fold changes in FACS-isolated ScLZ, ScPD, rSt and elSt in heterozygous (*H3f4*$^{MYC/wt}$) cells plotted against average gene expression levels in control (*H3f4*$^{wt/wt}$) cells. Data for full-length genic counts is shown. The numbers of significantly upregulated (red) or downregulated (blue) and non-significant (grey) genes are displayed on the top right (logFC ≥ 1, max. FDR = 0.05).

e, Venn diagram showing the overlap between genes upregulated in ScLZ, ScPD, rSt and elSt cells of *H3f*$^{MYCt/wt}$ versus *H3f4*$^{wt/wt}$ males.

f, Results of gene ontology (GO: Biological process) analysis of genes upregulated in ScPD and elSt cells. For ScLZ and rSt populations, we were unable to identify terms overrepresented in upregulated genes. A one-sided over representation hypergeometric test was used, followed by Benjamini–Hochberg correction for multiple testing.

g, Dot plot showing the percentage of up- and downregulated genes for each chromosome in ScLZ, ScPD and elSt cells. The bars represent the mean values for the selected DEGs. Data for rSt population is not shown due to low number of DEGs. Each dot has been derived from the edgeR estimation of DEGs (panel d) based on 4 biological replicates (n=4) for *H3f4*$^{wt/wt}$ and *H3f4*$^{MYC/wt-}$ animals.

h, Scatter plot showing differential expression (log2FC) in *H3f4*$^{MYC/wt}$ versus *H3f4*$^{wt/wt}$ elSts (FDR ≤ 0.05) (x axis) compared to differential expression (log2FC) in *H3f4*$^{H3.1/-}$ versus *H3f4*$^{wt/wt}$ elSts (FDR ≤ 0.05) (y axis).

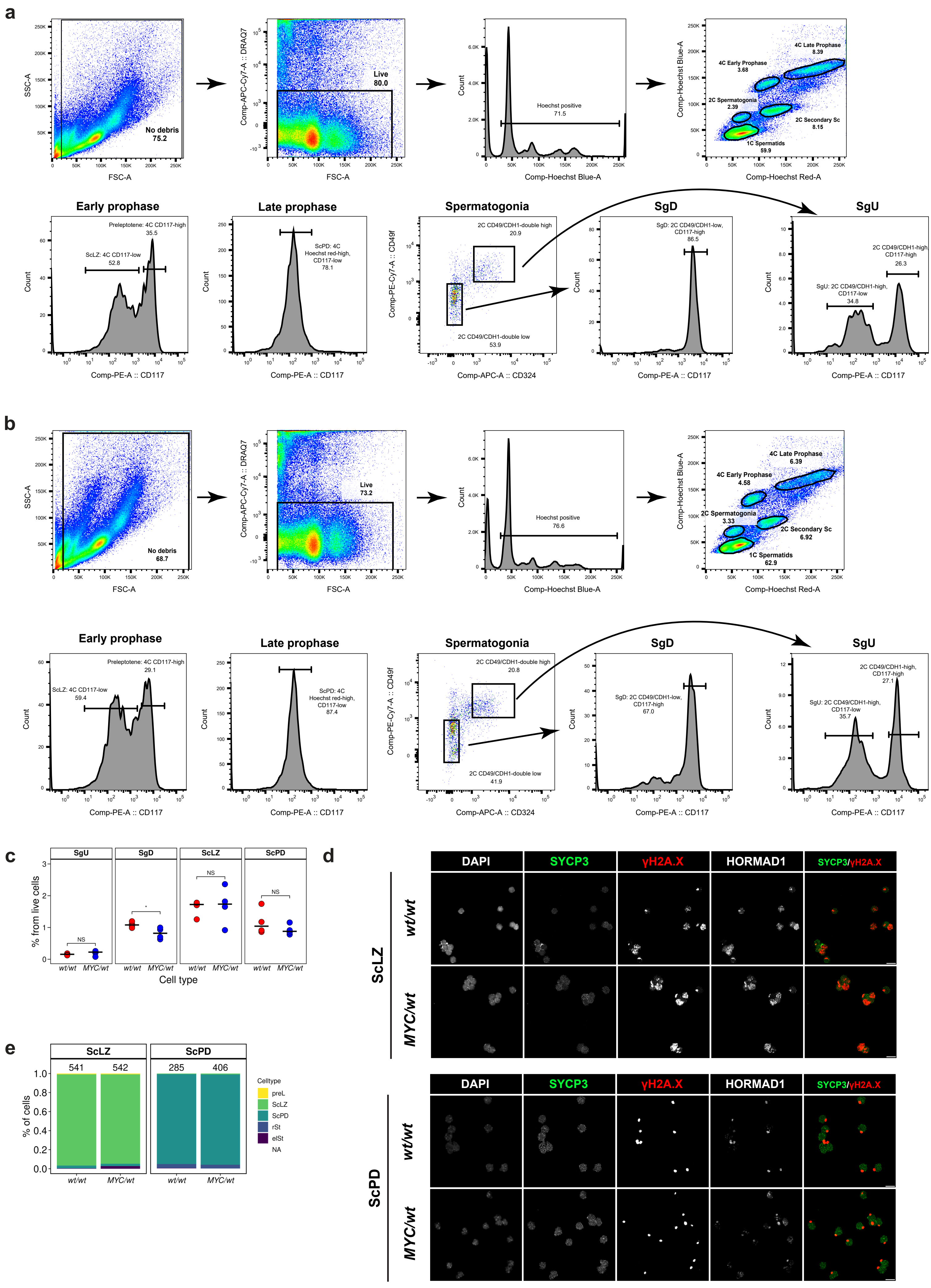

a
SSC-A
FSC-A
No debris
75.2
Comp-APC-Cy7-A :: DRAQ7
Live
80.0
Count
Hoechst positive
71.5
Comp-Hoechst Blue-A
Comp-Hoechst Red-A
4C Late Prophase
8.39
4C Early Prophase
3.68
2C Spermatogonia
2.39
2C Secondary Sc
8.15
1C Spermatids
59.9
Early prophase
Preleptotene: 4C CD117-high
35.5
ScLZ: 4C CD117-low
52.8
Comp-PE-A :: CD117
Late prophase
ScPD: 4C
Hoechst red-high,
CD117-low
78.1
Spermatogonia
2C CD49/CDH1-double high
20.9
2C CD49/CDH1-double low
53.9
Comp-PE-Cy7-A :: CD49f
Comp-APC-A :: CD324
SgD
SgD: 2C CD49/CDH1-low,
CD117-high
86.5
SgU
2C CD49/CDH1-high
CD117-high
26.3
SgU: 2C CD49/CDH1-high,
CD117-low
34.8
b
No debris
68.7
Live
73.2
Hoechst positive
76.6
4C Late Prophase
6.39
4C Early Prophase
4.58
2C Spermatogonia
3.33
2C Secondary Sc
6.92
1C Spermatids
62.9
Early prophase
Preleptotene: 4C CD117-high
29.1
ScLZ: 4C CD117-low
59.4
Late prophase
ScPD: 4C
Hoechst red-high,
CD117-low
87.4
Spermatogonia
2C CD49/CDH1-double high
20.8
2C CD49/CDH1-double low
41.9
SgD
SgD: 2C CD49/CDH1-low,
CD117-high
67.0
SgU
2C CD49/CDH1-high,
CD117-high
27.1
SgU: 2C CD49/CDH1-high,
CD117-low
35.7
c
SgU
SgD
ScLZ
ScPD
% from live cells
NS
*
wt/wt
MYC/wt
Cell type
d
DAPI
SYCP3
γH2A.X
HORMAD1
SYCP3/γH2A.X
ScLZ
wt/wt
MYC/wt
ScPD
e
ScLZ
ScPD
541
542
285
406
% of cells
Celltype
preL
ScLZ
ScPD
rSt
elSt
NA

Supplementary Figure 10. Gating parameters used for the FACS isolation of spermatogonia and spermatocytes

a-b, Representative FACS sorting profiles of germ cells with gating parameters from *H3f4*$^{wt/wt}$ control (a) and *H3f4*$^{MYC/wt}$ tagged (b) mice. FSC (forward size scatter) versus SSC (side size scatter) plot indicating the gate excluding debris. We next gated for DRAQ7-negative cells in the FCS versus DRAQ7 plot to select for living cells. The Hoechst-blue histogram indicates the gate with stained cells. The Hoechst-Red vs Hoechst-Blue plot indicates gates with populations of spermatogonia, spermatocytes and haploid spermatids. To obtain leptotene and zygotene spermatocytes (ScLZ), we gated for CD117 (C-KIT) – negative population in the CD117 histogram. The CD324 (E-cadherin) vs CD49f (Integrin alpha 6) plot shows the gates for undifferentiated spermatogonia (SgU, CD324-high, CD49f-high) and differentiating spermatogonia (SgD, CD324-low, CD49f-low). To sort for SgD, we gated for CD117-positive population and to sort for SgU, we gated for CD117-negative population.

c, Dot plot showing the percentages of sorted SgU, SgD, ScLZ and ScPD cells relative to all viable Hoechst-positive cells from *H3f4*$^{wt/wt}$ and *H3f4*$^{MYC/wt}$ animals. Two-sided t-test was performed to compare between genotypes. * $p < 0.05$, NS – not significant.

d, Purity control of FACS-sorted populations of ScLZ and ScPD spermatocytes. Representative immunofluorescence staining of spread cells with antibodies against synaptonemal complex protein 3 (SYCP3), phosphorylated H2A.X (γH2A.X) marking damaged DNA and HORMAD1 marking unsynapsed chromosome axes. Scale bar: 20 µm.

e, Stacked bar plot showing the relative percentages of cell types withing the target ScLZ and ScPD populations of sorted cells. Data from two biological replicates were pooled, numbers on the top represent the total number of cells analyzed. NA – cell type not possible to identify. We observed no differences in meiotic cell type purities between genotypes.

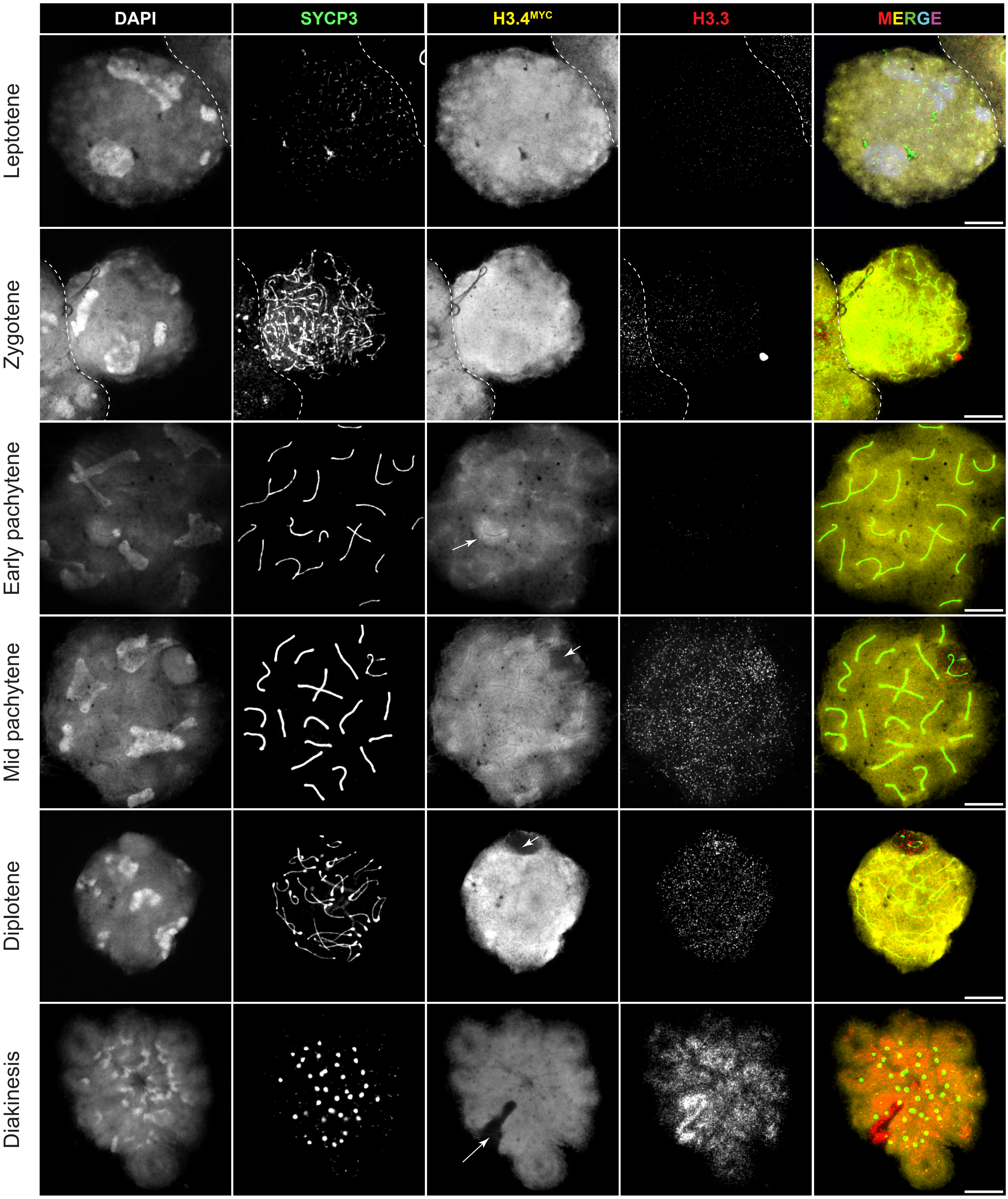
DAPI
SYCP3
H3.4MYC
H3.3
MERGE
Leptotene
Zygotene
Early pachytene
Mid pachytene
Diplotene
Diakinesis

Supplementary Figure 11. H3.4 is evicted from the X and Y chromosomes in mid-pachytene spermatocytes

Meiotic spreads showing temporal dynamics of $H3.4^{MYC}$ and H3.3 proteins during progression through meiotic prophase I. The X and Y chromosomes undergoing eviction of H3.4 are indicated by white arrows. Scale bar: 10 µm.

a

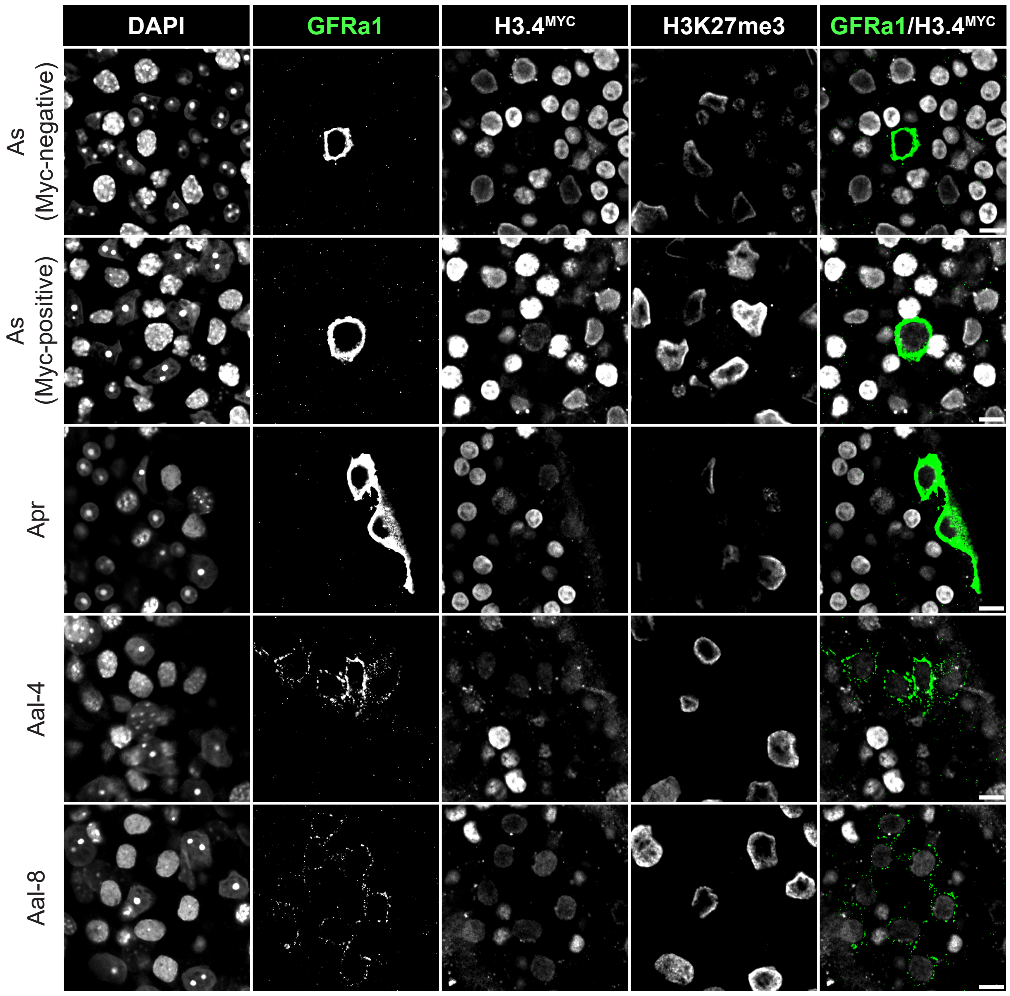
DAPI
GFRa1
H3.4MYC
H3K27me3
GFRa1/H3.4MYC
As (Myc-negative)
As (Myc-positive)
Apr
Aal-4
Aal-8

b

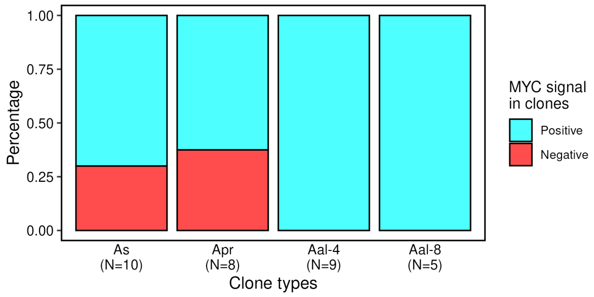
1.00
0.75
0.50
0.25
0.00
Percentage
As (N=10)
Apr (N=8)
Aal-4 (N=9)
Aal-8 (N=5)
Clone types
MYC signal in clones
Positive
Negative

Supplementary Figure 12. H3.4^{MYC} is expressed in undifferentiated spermatogonia

a, Whole-mount immunofluorescence images showing H3.4^{MYC} expression in different clones of GRFα1-positive spermatogonia with different chain length: A_s (single), A_{pr} (paired), $A_{al\text{-}4}$ (chain of 4 aligned cells) and $A_{al\text{-}8}$ (chain of 8 aligned cells). For A_s, an example of a clone positive or negative for H3.4^{MYC} are displayed. Scale bar: 10 µm.

b, Stacked bar plot showing the proportion of GRFα1-positive spermatogonia clones (A_s, A_{pr}, $A_{al\text{-}4}$ and $A_{al\text{-}8}$) positive or negative for H3.4^{MYC} staining. N corresponds to the number of clones assessed.

a

ULI-NChIP

Celltype	Replicate	Genotype	ChIP	Type	Library Name
SgU	rep1	wt/wt	H3.3	PE	SgU_H3.3_rep1
SgU	rep2	wt/wt	H3.3	PE	SgU_H3.3_rep2
SgU	rep3	MYC/wt	H3.3	PE	SgU_H3.3_rep3
SgU	rep4	MYC/wt	H3.3	PE	SgU_H3.3_rep4
SgD	rep1	wt/wt	H3.3	PE	SgD_H3.3_rep1
SgD	rep2	wt/wt	H3.3	PE	SgD_H3.3_rep2
SgD	rep3	MYC/wt	H3.3	PE	SgD_H3.3_rep3
SgD	rep4	MYC/wt	H3.3	PE	SgD_H3.3_rep4
ScLZ	rep1	wt/wt	H3.3	PE	ScLZ_H3.3_rep1
ScLZ	rep2	wt/wt	H3.3	PE	ScLZ_H3.3_rep2
ScLZ	rep3	MYC/wt	H3.3	PE	ScLZ_H3.3_rep3
ScLZ	rep4	MYC/wt	H3.3	PE	ScLZ_H3.3_rep4
ScPD	rep1	wt/wt	H3.3	PE	ScPD_H3.3_rep1
ScPD	rep2	wt/wt	H3.3	PE	ScPD_H3.3_rep2
ScPD	rep3	MYC/wt	H3.3	PE	ScPD_H3.3_rep3
ScPD	rep4	MYC/wt	H3.3	PE	ScPD_H3.3_rep4
SgU	rep1	MYC/wt	MYC	PE	SgU_MYC_rep1
SgU	rep2	MYC/wt	MYC	PE	SgU_MYC_rep2
SgD	rep1	MYC/wt	MYC	PE	SgD_MYC_rep1
SgD	rep2	MYC/wt	MYC	PE	SgD_MYC_rep2
ScLZ	rep1	MYC/wt	MYC	PE	ScLZ_MYC_rep1
ScLZ	rep2	MYC/wt	MYC	PE	ScLZ_MYC_rep2
ScPD	rep1	MYC/wt	MYC	PE	ScPD_MYC_rep1
ScPD	rep2	MYC/wt	MYC	PE	ScPD_MYC_rep2
SgD	rep1	wt/wt	H3K4me3	SE	SgD_H3K4me3_rep1[1]
SgD	rep2	wt/wt	H3K4me3	PE	SgD_H3K4me3_rep2[1]
ScLZ	rep1	wt/wt	H3K4me3	SE	ScLZ_H3K4me3_rep1
ScPD	rep1	wt/wt	H3K4me3	SE	ScPD_H3K4me3_rep1
SgD	rep1	wt/wt	H3K27me3	SE	SgD_H3K27me3_rep1[1]
SgD	rep2	wt/wt	H3K27me3	SE	SgD_H3K27me3_rep2[1]
SgD	rep3	wt/wt	H3K27me3	SE	SgD_H3K27me3_rep3[1]
SgD	rep4	wt/wt	H3K27me3	SE	SgD_H3K27me3_rep4[1]
ScLZ	rep1	wt/wt	H3K27me3	SE	ScLZ_H3K27me3_rep1[1]
ScPD	rep1	wt/wt	H3K27me3	SE	ScPD_H3K27me3_rep1[1]
ScPD	rep2	wt/wt	H3K27me3	SE	ScPD_H3K27me3_rep2[1]

[1]Bocker *et al*., (2025) Nat Commun

b

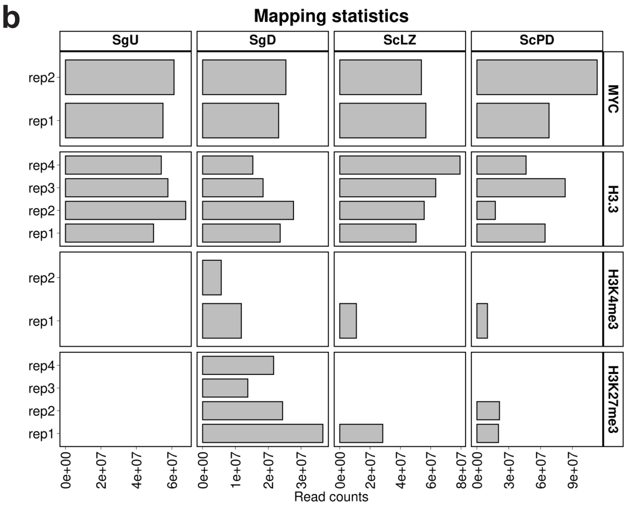

c

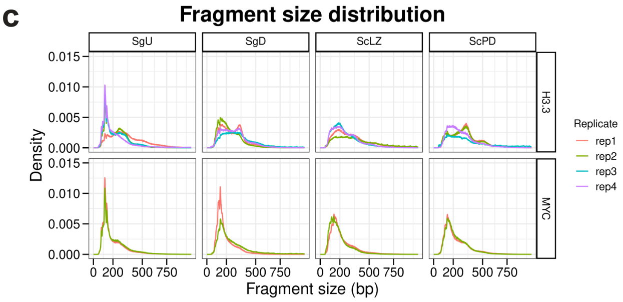

d

conventional ChIP

Cell type	Replicate	Genotype	ChIP	Type	Library Name
ScPD	rep1	wt/wt	H3.3	SE	ScPD_H3.3_rep1
ScPD	rep2	wt/wt	H3.3	SE	ScPD_H3.3_rep2
ScPD	rep3	MYC/wt	H3.3	SE	ScPD_H3.3_rep3
ScPD	rep4	MYC/wt	H3.3	SE	ScPD_H3.3_rep4
rSt	rep1	wt/wt	H3.3	SE	rSt_H3.3_rep1
rSt	rep2	wt/wt	H3.3	SE	rSt_H3.3_rep2
rSt	rep3	MYC/wt	H3.3	SE	rSt_H3.3_rep3
rSt	rep4	MYC/wt	H3.3	SE	rSt_H3.3_rep4
ScPD	rep1	MYC/wt	MYC	SE	ScPD_MYC_rep1
ScPD	rep2	MYC/wt	MYC	SE	ScPD_MYC_rep2
rSt	rep1	MYC/wt	MYC	SE	rSt_MYC_rep1
rSt	rep2	MYC/wt	MYC	SE	rSt_MYC_rep2
ScPD	rep1	wt/wt	H3K4me3	SE	ScPD_H3K4me3_rep1
rSt	rep1	wt/wt	H3K4me3	SE	rSt_H3K4me3_rep1[2]
ScPD	rep1	wt/wt	H3K27me3	SE	ScPD_H3K27me3_rep1
rSt	rep1	wt/wt	H3K27me3	SE	rSt_H3K27me3_rep1[2]
ScPD	rep1	wt/wt	MYC	SE	ScPD_MYC
ScPD	rep2	wt/wt	MYC	SE	ScPD_MYC
rSt	rep1	wt/wt	MYC	SE	rSt_MYC
rSt	rep2	wt/wt	MYC	SE	rSt_MYC
ScPD	rep1	wt/wt	Input	SE	ScPD_Input_rep1
ScPD	rep2	wt/wt	Input	SE	ScPD_Input_rep2
rSt	rep1	wt/wt	Input	SE	rSt_Input_rep1
rSt	rep2	wt/wt	Input	SE	rSt_Input_rep2

[2]Erkek *et al*., (2013) NSMB

e

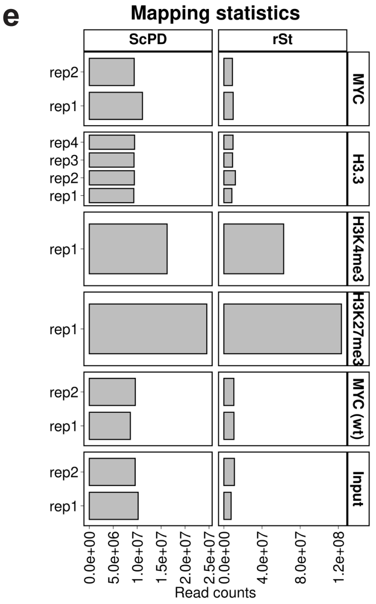

g

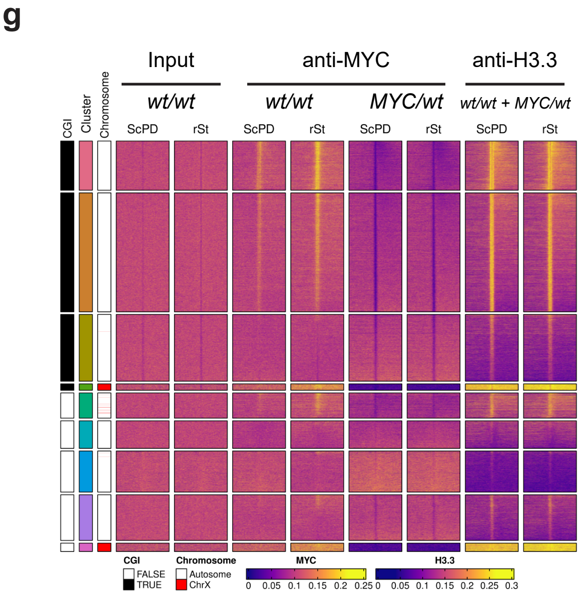

Supplementary Figure 13. Quality control of ChIP-seq libraries used in this study

a, Names of ChIP-seq libraries generated in this study using an ultra-low native ULI-NChIP method or previously published (Bocker *et al.*[57]). Libraries related to different antibodies are highlighted in different shades of gray.

b, Mapping statistics of libraries displayed in panel a.

c, Fragment size distribution of reads from libraries in panel a (only for paired-end libraries)

d, Names of ChIP-seq libraries generated in this study using a conventional ChIP method (see Materials and Methods) or previously published (Erkek *et al.*[61]). Libraries related to different antibodies are highlighted in different shades of gray.

e, Mapping statistics of libraries displayed in panel d.

g, Heatmap comparing ChIP-signal in input, anti-MYC libraries (in $H3f4^{wt/wt}$ and MYC-expressing $H3f4^{MYC/wt}$ animals separately) and anti-H3.3 libraries (signal pooled from control $H3f4^{wt/wt}$ and MYC-expressing $H3f4^{MYC/wt}$ animals). The intensity scale for input and anti-MYC libraries is the same. The order of clusters and genes is the same as in Fig. 3a.

The experiment shows the presence of $H3.4^{MYC}$ in chromatin of ScPD and rSt cells at regions up- and downstream of TSS in a sequence-context specific manner. In cells from transgenic animals we observe the following: At CGI promoter genes, $H3.4^{MYC}$ occupancy is visible in flanking regions surrounding TSS while the signal is reduced at the central CGI regions (see Fig 3a for CGI information). At non-CGI promoter genes, $H3.4^{MYC}$ occupancy is largely uniform across the regions except for genes at which nucleosome turnover is observed. Finally, $H3.4^{MYC}$ occupancy is absent at X-linked genic regions indicative of MSCI-related nucleosome replacement of H3.4 by H3.3. H3.3 occupancy reflects an inverse occupancy pattern as seen for $H3.4^{MYC}$, with high levels at regions having undergone eviction of $H3.4^{MYC}$, either at TSS or along the X chromosome. In cells of wildtype control mice, the anti-MYC antibody reveals a non-uniform pattern around TSS of CGI genes and partially along X-linked genic regions, opposite of the patterns observed in transgenic $H3.4^{MYC}$ expressing cells. Since $H3.4^{MYC}$ enrichment in $H3f4^{wt/wt}$ mice does not reflect the presence or absence of the DNA-sequence motif of the endogenous MYC protein at promoter regions (data analysis not shown), it may point to cross-reactivity with other epitopes. Together, given the inverse ChIP-signal patterns of $H3.4^{MYC}$ and H3.3 proteins, we consider the $H3.4^{MYC}$ signal as representing genuine $H3.4^{MYC}$ occupancy in transgenic animals.

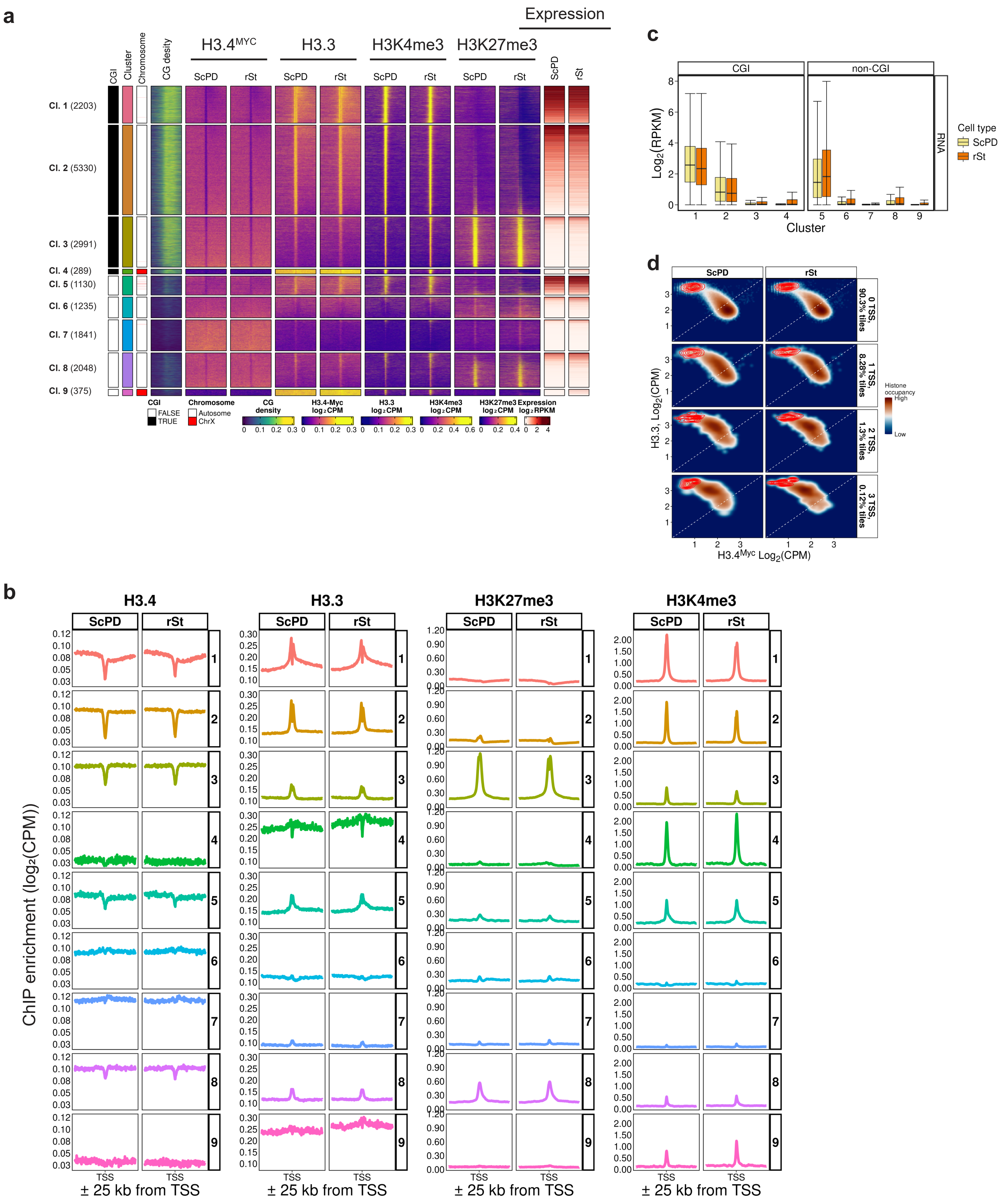
a
Expression
CGI
Cluster
Chromosome
CG desity
H3.4MYC
H3.3
H3K4me3
H3K27me3
ScPD
rSt
Cl. 1 (2203)
Cl. 2 (5330)
Cl. 3 (2991)
Cl. 4 (289)
Cl. 5 (1130)
Cl. 6 (1235)
Cl. 7 (1841)
Cl. 8 (2048)
Cl. 9 (375)
CGI
FALSE
TRUE
Chromosome
Autosome
ChrX
CG density
H3.4-Myc log2CPM
H3.3 log2CPM
H3K4me3 log2CPM
H3K27me3 log2CPM
Expression log2RPKM
c
CGI
non-CGI
RNA
Log2(RPKM)
Cluster
Cell type
ScPD
rSt
d
0 TSS, 90.3% tiles
1 TSS, 8.28% tiles
2 TSS, 1.3% tiles
3 TSS, 0.12% tiles
Histone occupancy
High
Low
H3.3, Log2(CPM)
H3.4Myc Log2(CPM)
b
H3.4
H3.3
H3K27me3
H3K4me3
ChIP enrichment (log2(CPM))
TSS
± 25 kb from TSS

Supplementary Figure 14. Genome localization of H3 variants in ScPD and rSt cells

a, Heatmap displaying chromatin states at TSS regions (± 25 kb; log2CPM) and gene expression levels (log2RPKM) of CGI- and nonCGI-containing genes measured in FACS-purified ScPD and rSt cells. Regions were grouped into 9 gene clusters by k-means clustering on chromatin variables performed separately for CGI- and non-CGI-type promoters.

The number of gene promoters is displayed for each cluster. CG density (±1 kb around TSS), is indicated in percentage. The ChIP-seq for H3K4me3 and H3K27me3 data in rSt were obtained from Erkek *et al.*[61] and RNA expression data in ScPD and rSt from Gill *et al.*[58]. The order of clusters and genes is the same as in Fig. 3a.

b, Metaplots showing quantification of ChIP-seq data at TSS regions (± 25 kb; log2CPM) in clusters 1-9, as displayed in panel a.

c, Boxplot showing quantification of expression of genes (in log2RPKM) belonging to clusters 1-9 in panel a.

d, Density scatter plots displaying log2CPM counts of $H3.4^{MYC}$ (x-axis) and H3.3 (y-axis) on genomic tiles that contain 0, 1, 2 or 3+ transcription units with TSS in ScPD and rSt cells. Red contour lines represent the density of tiles located on the X chromosome. The presence of a transcription unit with TSS was defined by an overlap of a given tile with a region ±1 kb around TSS.

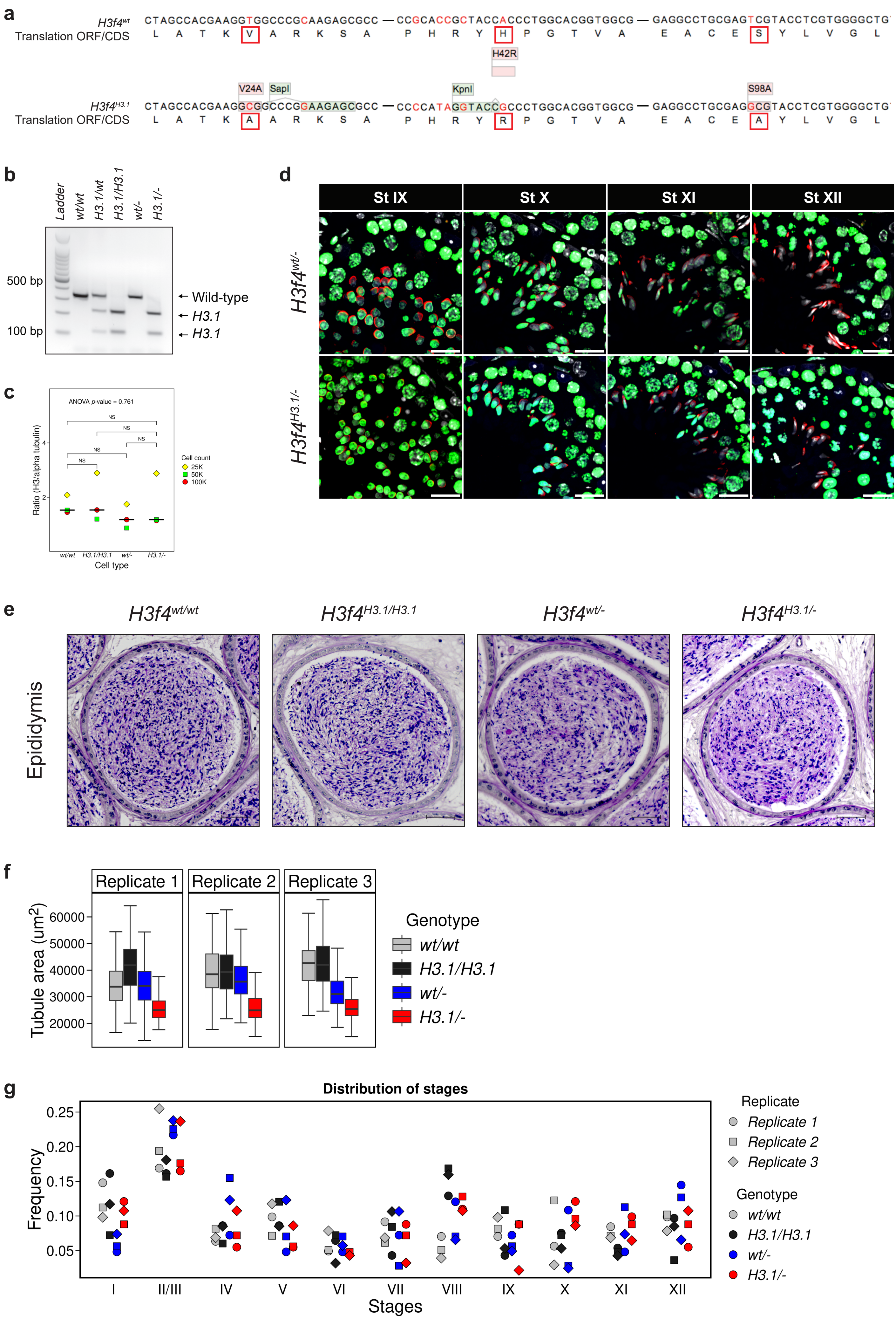

a
H3f4wt
Translation ORF/CDS
CTAGCCACGAAGGTGGCCCGCAAGAGCGCC — CCGCACCGCTACCACCCTGGCACGGTGGCG — GAGGCCTGCGAGTCGTACCTCGTGGGGCTG
L A T K V A R K S A P H R Y H P G T V A E A C E S Y L V G L
H42R
V24A
SapI
KpnI
S98A
H3f4H3.1
CTAGCCACGAAGGCGGCCCGGAAGAGCGCC — CCCCATAGGTACCGCCCTGGCACGGTGGCG — GAGGCCTGCGAGGCGTACCTCGTGGGGCTG
L A T K A A R K S A P H R Y R P G T V A E A C E A Y L V G L
b
Ladder
wt/wt
H3.1/wt
H3.1/H3.1
wt/-
H3.1/-
500 bp
100 bp
← Wild-type
← H3.1
← H3.1
c
ANOVA p-value = 0.761
NS
Ratio (H3/alpha tubulin)
Cell count
25K
50K
100K
wt/wt
H3.1/H3.1
wt/-
H3.1/-
Cell type
d
St IX
St X
St XI
St XII
H3f4wt/-
H3f4H3.1/-
e
H3f4wt/wt
H3f4H3.1/H3.1
H3f4wt/-
H3f4H3.1/-
Epididymis
f
Replicate 1
Replicate 2
Replicate 3
Tubule area (um2)
60000
50000
40000
30000
20000
Genotype
wt/wt
H3.1/H3.1
wt/-
H3.1/-
g
Distribution of stages
Frequency
0.25
0.20
0.15
0.10
0.05
I
II/III
IV
V
VI
VII
VIII
IX
X
XI
XII
Stages
Replicate
Replicate 1
Replicate 2
Replicate 3
Genotype
wt/wt
H3.1/H3.1
wt/-
H3.1/-

Supplementary Figure 15. Generation of the *H3f4*$^{H3.1}$ allele by CRISPR/Cas9 gene editing.

a, Schematic representation of the *H3f4*^{wt} wildtype and *H3f4*$^{H3.1}$ modified alleles, with corresponding H3.4 to H3.1-specific amino acid substitutions (in red boxes). Variant positions in DNA sequences encoding H3.4 and H3.1 are indicated in red, while relevant restriction sites are shown in light green. Abbreviations: V24A, Valine 24 to Alanine; H42R, Histidine 42 to Arginine; S98A, Serine 98 to Alanine. CDS, coding sequence; ORF, open reading frame.

b, PCR genotyping results of the *H3.1* mouse model. The following genotypes are shown: *H3f4*$^{wt/wt}$, *H3f4*$^{H3.1/wt}$, *H3f4*$^{H3.1/H3.1}$, *H3f4*$^{wt/-}$ and *H3f4*$^{H3.1/-}$. Primers and PCR protocols used for genotyping are provided in Supplementary Data 8 and 9, respectively. The PCR products were digested with KpnI into a 338bp restriction fragment for the wildtype allele and two bands of 222 bp and 116 bp for the *H3f4*$^{H3.1}$ allele. A hundred base pair ladder is shown for size comparison.

c, ImageJ quantification of Western blot data shown in Fig. 4c and calculated as the ratio of intensities of bands detected by the pan-H3 antibody over the bands detected by alpha tubulin antibody. Two-sided t-test was performed for each comparison group. NS – not significant. The *p*-value obtained from an ANOVA test is provided at the top.

d, Representative immunofluorescence images of stage IX to XII seminiferous tubules of *H3f4*$^{wt/-}$ and *H3f4*$^{H3.1/-}$ animals, co-stained for DAPI (grey), an antibody that recognizes H3.1, H3.2 and H3.4 (#ab34)[62] (green) and acrosome (red). Scale bar: 20 µm.

e, PAS-haematoxylin staining of caudal epidydimal tissues obtained from *H3f4*$^{wt/wt}$, *H3f4*$^{H3.1/H3.1}$, *H3f4*$^{wt/-}$ and *H3f4*$^{H3.1/-}$ mice. Scale bar: 50 µm.

f, Boxplots showing the area of segmented seminiferous tubules used in the quantification analysis shown in Fig. 4i. Data for three biological replicates per genotype are shown.

g, Distribution of developmental stages of the seminiferous tubule cycle in testes of 3 mice per genotype. Cells in stage V and X tubules were used for automated cell quantification analysis as shown in Fig. 4i. Staging was manually performed as described before[66]. Each dot represents the frequency of a given stage per biological replicate.

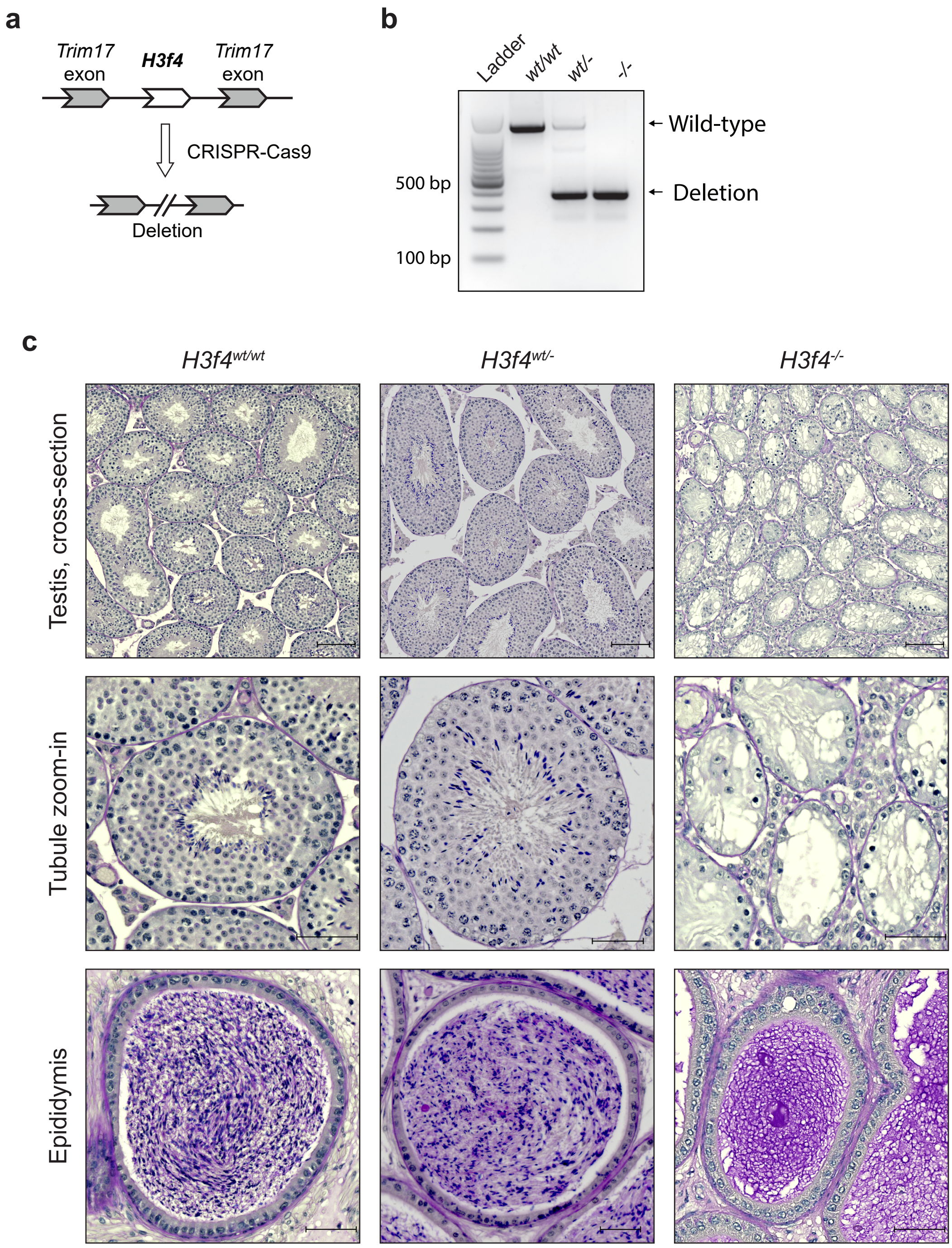
a
Trim17 exon
H3f4
Trim17 exon
CRISPR-Cas9
Deletion
b
Ladder
wt/wt
wt/-
-/-
500 bp
100 bp
Wild-type
Deletion
c
H3f4wt/wt
H3f4wt/-
H3f4-/-
Testis, cross-section
Tubule zoom-in
Epididymis

Supplementary Figure 16. Deletion of the *H3f4* gene leads to infertility.

a, CRISPR/Cas9 strategy to delete the *H3f4* gene. Sequences of sgRNAs are indicated in Supplementary Data 6.

b, Genotyping results of mice with wildtype ($H3f4^{wt/wt}$), heterozygous ($H3f4^{wt/-}$) and *H3f4* knock-out ($H3f4^{-/-}$) genotypes. PCR using primers (Supplementary Datas 8 and 9) that bind up- and downstream of the deletion shows fragment sizes of 1180bp (wildtype allele) and 330bp (deleted allele). Ladder in multiples of hundred base pairs is shown for size comparison.

c, PAS-haematoxylin-stained histological cross-sections of testes and epididymal tissues obtained from $H3f4^{wt/wt}$, $H3f4^{wt/-}$ and $H3f4^{-/-}$ mice. Scale bars: 100 µm (cross-section overview) and 50 µm (tubule zoom-in and epididymis).

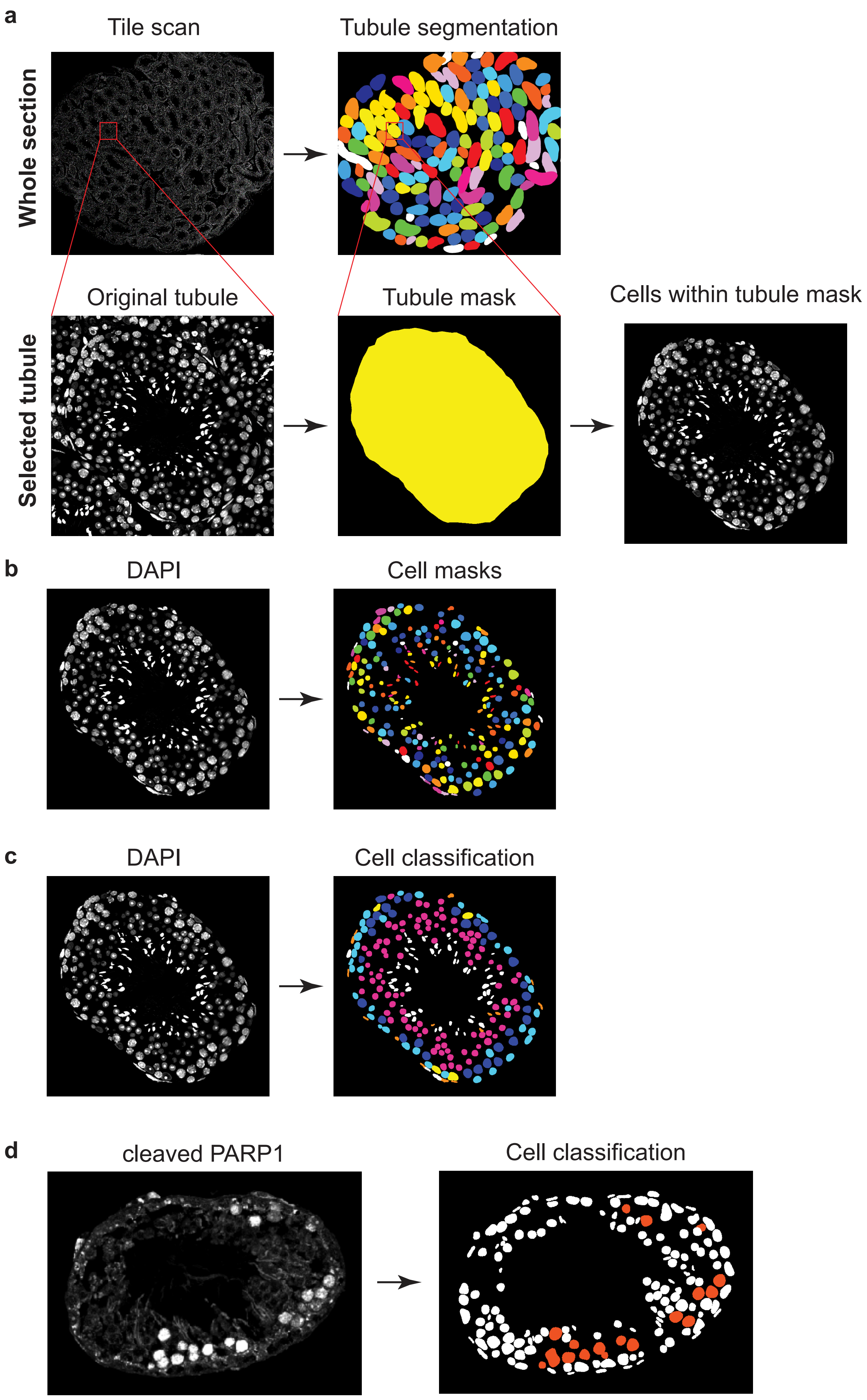
a
Tile scan
Tubule segmentation
Whole section
Original tubule
Tubule mask
Cells within tubule mask
Selected tubule
b
DAPI
Cell masks
c
DAPI
Cell classification
d
cleaved PARP1
Cell classification

Supplementary Figure 17. The pipeline for automated quantification of male germ cells

a, Example of tubule segmentation on an 8-binned stitched scan (DAPI channel used as an input). Raw DAPI and tubule masks are shown for the whole scan (upper panels) and for a selected tubule example (bottom).

b, Example of StarDist-based cell segmentation after manual curation of cell shapes. DAPI images were used as input information for cell segmentation.

c, Example of a multi-type cell classification results performed by Ilastik software[116] on a single channel (DAPI) channel. Ground truth annotation was used to assign classes to cells in a learning phase. Different colors represent cell different cell types.

d, Example of a single-type cell classification results performed by Ilastik software[116] on a single channel (cleaved PARP1) channel. Ground truth annotation was used to assign classes to cells in a learning phase.

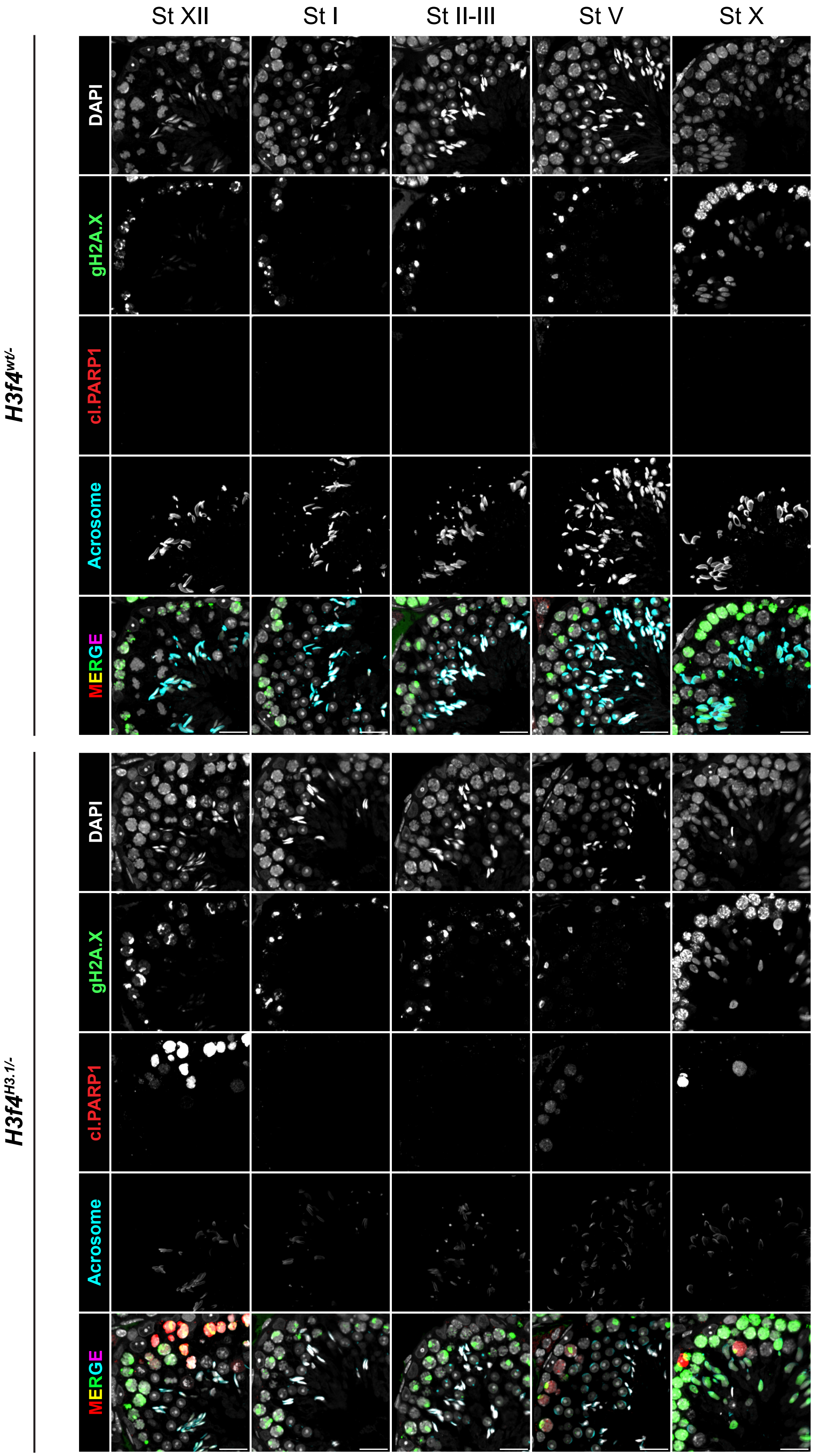
St XII
St I
St II-III
St V
St X
H3f4wt/-
DAPI
gH2A.X
cl.PARP1
Acrosome
MERGE
H3f4H3.1/-
DAPI
gH2A.X
cl.PARP1
Acrosome
MERGE

Supplementary Figure 18. Examples of cleaved PARP1 signal in testicular tubules in *H3f4*$^{wt/-}$ and *H3f4*$^{H3.1/-}$ animals

Representative zoom-up examples of stage I, II-III, V, X and XII seminiferous tubules, related to Fig. 5a. Scale bar: 20 um.

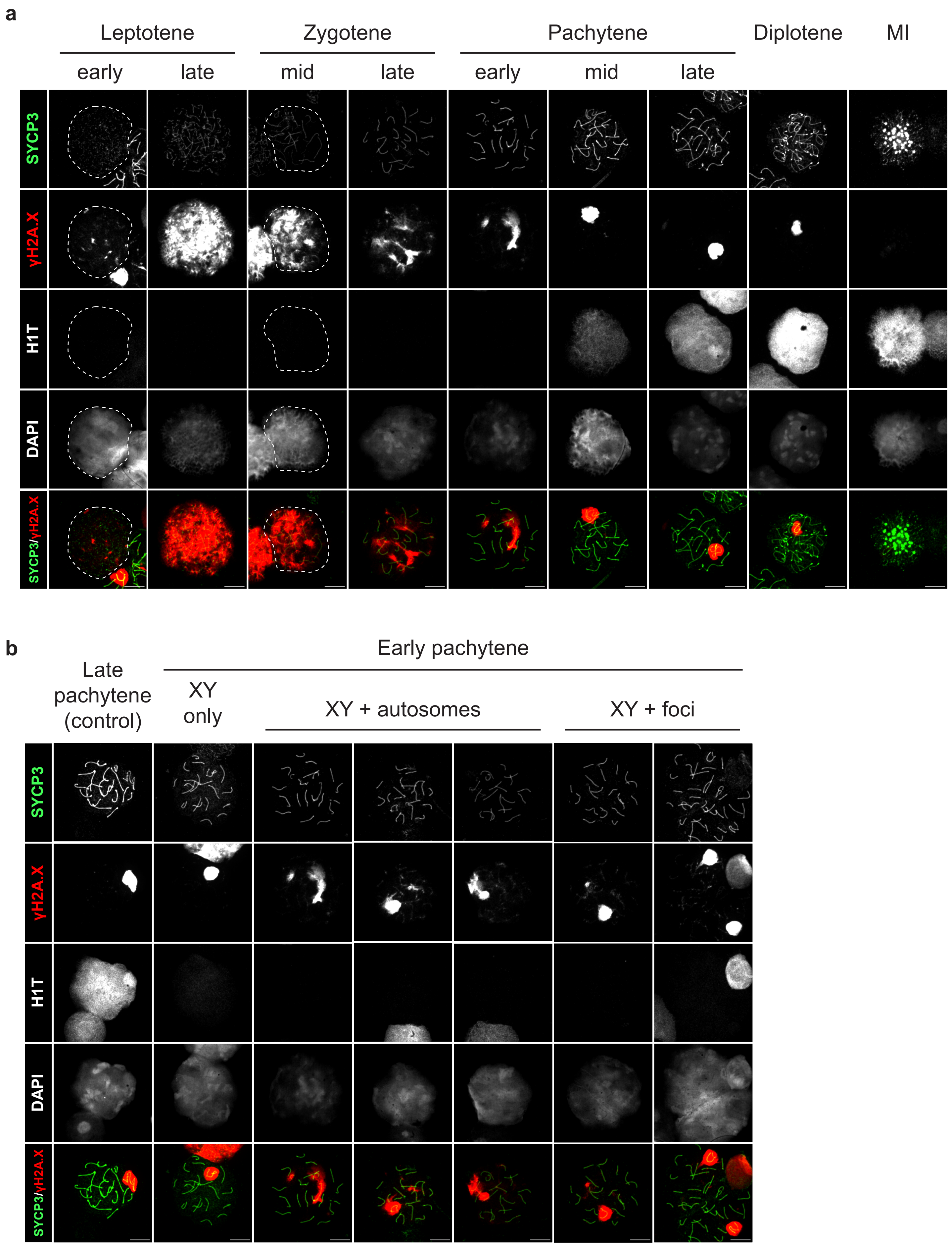
a
Leptotene
Zygotene
Pachytene
Diplotene
MI
early
late
mid
late
early
mid
late
SYCP3
γH2A.X
H1T
DAPI
SYCP3/γH2A.X
b
Late pachytene (control)
Early pachytene
XY only
XY + autosomes
XY + foci
SYCP3
γH2A.X
H1T
DAPI
SYCP3/γH2A.X

Supplementary Figure 19. Synapsis and double strand break formation and repair during meiotic prophase in *H3f4*$^{wt/-}$ control mice

a, Examples of SYCP3 and γH2A.X signals in spread of spermatocytes during their developmental time course of prophase I in *H3f4*$^{wt/-}$ animals. To assess the developmental progression of spermatocytes through pachytene substages, we used immunodetection of H1T histone variant incorporation into meiotic chromatin. Scale bar: Scale bar: 10 μm.

b, Examples of SYCP3 and γH2A.X signals in spread of early pachytene spermatocyte nuclei (lacking H1T expression) in *H3f4*$^{wt/-}$ animals. Scale bar: 10 μm.

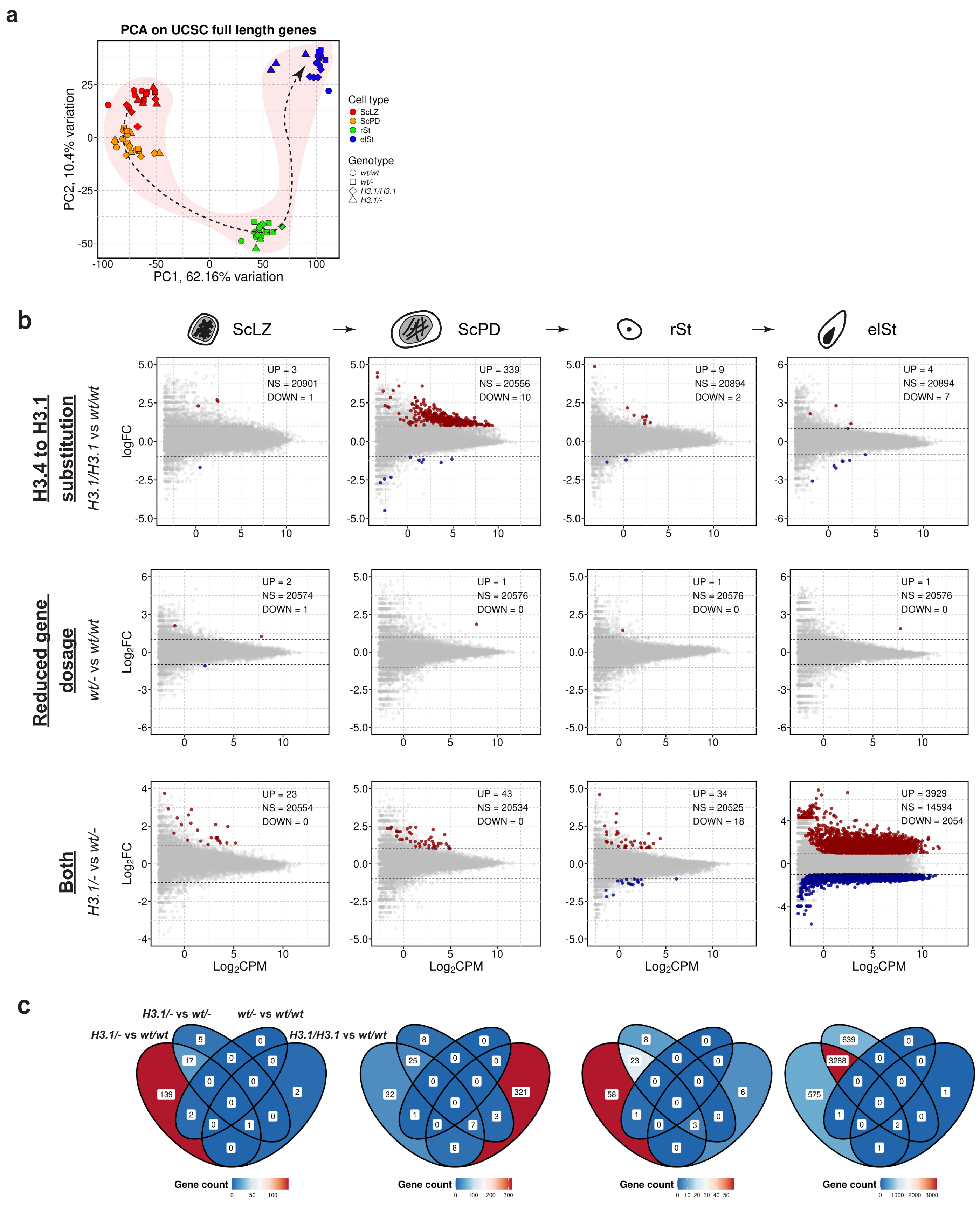
a
PCA on UCSC full length genes
PC2, 10.4% variation
PC1, 62.16% variation
Cell type
ScLZ
ScPD
rSt
elSt
Genotype
wt/wt
wt/-
H3.1/H3.1
H3.1/-
b
ScLZ
ScPD
rSt
elSt
H3.4 to H3.1 substitution
H3.1/H3.1 vs wt/wt
Reduced gene dosage
wt/- vs wt/wt
Both
H3.1/- vs wt/-
logFC
Log2FC
Log2CPM
UP = 3
NS = 20901
DOWN = 1
UP = 339
NS = 20556
DOWN = 10
UP = 9
NS = 20894
DOWN = 2
UP = 4
NS = 20894
DOWN = 7
UP = 2
NS = 20574
DOWN = 1
UP = 1
NS = 20576
DOWN = 0
UP = 1
NS = 20576
DOWN = 0
UP = 1
NS = 20576
DOWN = 0
UP = 23
NS = 20554
DOWN = 0
UP = 43
NS = 20534
DOWN = 0
UP = 34
NS = 20525
DOWN = 18
UP = 3929
NS = 14594
DOWN = 2054
c
H3.1/- vs wt/-
wt/- vs wt/wt
H3.1/- vs wt/wt
H3.1/H3.1 vs wt/wt
Gene count

Supplementary Figure 20. Transcriptional impact of H3.4 to H3.1 substitution and/or of reduced *H3f4* gene dosage.

a, Principal component analysis (PCA) comparing RNA-seq expression of UCSC-annotated genes among biological replicates and genotypes in samples that were used in the study.

b, Scatter plots showing differential gene expression (log2FC) in $H3f4^{H3.1/-}$ versus $H3f4^{wt/-}$ FACS-isolated ScLZ, ScPD, rSt and elSt cells plotted against gene expression levels in $H3f4^{wt/-}$ cells (top row), in $H3f4^{H3.1/H3.1}$ against $H3f4^{wt/wt}$ (middle row) and in $H3f4^{wt/-}$ against $H3f4^{wt/wt}$ (bottom row). Numbers of significantly upregulated (red), downregulated (blue) and non-significant (grey) genes are displayed (logFC ≥ 1, FDR ≤ 0.05).

c, Venn diagrams showing the overlap between genes upregulated in the contrasts shown in Fig. 6a and Supplementary Fig. 20b.

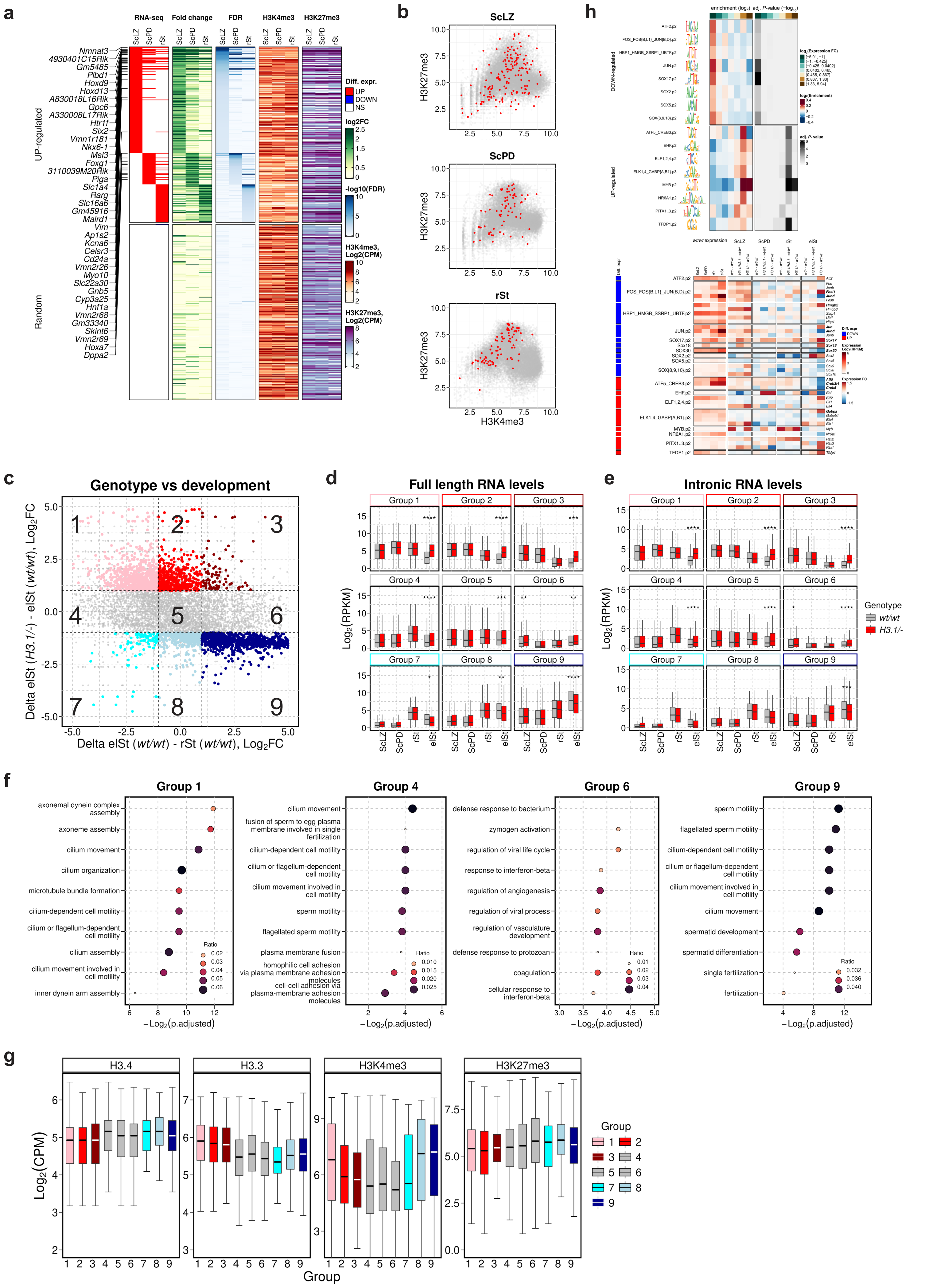
a
RNA-seq
Fold change
FDR
H3K4me3
H3K27me3
ScLZ
ScPD
rSt
UP-regulated
Random
Nmnat3
4930401C15Rik
Gm5485
Plbd1
Hoxd9
Hoxd13
A830018L16Rik
Gpc6
A330008L17Rik
Htr1f
Six2
Vmn1r181
Nkx6-1
Msl3
Foxg1
3110039M20Rik
Piga
Slc1a4
Rarg
Slc16a6
Gm45916
Malrd1
Vim
Ap1s2
Kcna6
Celsr3
Cd24a
Vmn2r26
Myo10
Slc22a30
Gnb5
Cyp3a25
Hnf1a
Vmn2r68
Gm33340
Skint6
Vmn2r69
Hoxa7
Dppa2
Diff. expr.
UP
DOWN
NS
log2FC
-log10(FDR)
H3K4me3, Log2(CPM)
H3K27me3, Log2(CPM)
b
ScLZ
ScPD
rSt
H3K27me3
H3K4me3
h
DOWN-regulated
UP-regulated
enrichment (log₂)
adj. P-value (−log₁₀)
wt/wt expression
Diff. expr
Expression Log2(RPKM)
Expression FC
c
Genotype vs development
Delta elSt (H3.1/-) - elSt (wt/wt), Log2FC
Delta elSt (wt/wt) - rSt (wt/wt), Log2FC
d
Full length RNA levels
e
Intronic RNA levels
Group 1
Group 2
Group 3
Group 4
Group 5
Group 6
Group 7
Group 8
Group 9
Log2(RPKM)
Genotype
wt/wt
H3.1/-
f
Group 1
axonemal dynein complex assembly
axoneme assembly
cilium movement
cilium organization
microtubule bundle formation
cilium-dependent cell motility
cilium or flagellum-dependent cell motility
cilium assembly
cilium movement involved in cell motility
inner dynein arm assembly
Group 4
cilium movement
fusion of sperm to egg plasma membrane involved in single fertilization
cilium-dependent cell motility
cilium or flagellum-dependent cell motility
cilium movement involved in cell motility
sperm motility
flagellated sperm motility
plasma membrane fusion
homophilic cell adhesion via plasma membrane adhesion molecules
cell-cell adhesion via plasma-membrane adhesion molecules
Group 6
defense response to bacterium
zymogen activation
regulation of viral life cycle
response to interferon-beta
regulation of angiogenesis
regulation of viral process
regulation of vasculature development
defense response to protozoan
coagulation
cellular response to interferon-beta
Group 9
sperm motility
flagellated sperm motility
cilium-dependent cell motility
cilium or flagellum-dependent cell motility
cilium movement involved in cell motility
cilium movement
spermatid development
spermatid differentiation
single fertilization
fertilization
Ratio
−Log2(p.adjusted)
g
H3.4
H3.3
H3K4me3
H3K27me3
Log2(CPM)
Group

Supplementary Figure 21. Major transcriptional changes in elongating spermatids of *H3f4*$^{H3.1/-}$ mice

a, Heatmap showing ChIP-seq enrichments of H3K4me3 and H3K27me3 in control samples at promoters of genes (TSS ±1 kb; log2(CPM)) upregulated in ScLZ, ScPD and rSt cells of *H3f4*$^{H3.1/-}$ animals (Fig. 6a). For reference, a randomly selected set of genes is shown below. The EdgeR results for RNA-seq data (significance, fold-change and FDR) are shown for each gene. Names of genes commonly upregulated in at least two cell populations are indicated on the left. H3K27me3 data in ScLZ and ScPD was obtained from Bocker *et al.*[57], H3K4me3 and H3K27me3 in rSt was obtained from Erkek *et al.*[61]

b, Scatter plots showing the enrichment of H3K27me3 versus H3K4me3 at promoters (TSS ±1 kb; log2(CPM)) of all genes (grey) and of the genes upregulated in ScLZ, ScPD and rSt cells (red) (Fig. 6a).

c, Scatter plot showing differential expression (log2FC) of non-CGI type promoter genes in *H3f4*$^{wt/wt}$ elSts versus *H3f4*$^{wt/wt}$ rSts (FDR ≤ 0.05) (x axis) compared to differential expression (log2FC) in *H3f4*$^{H3.1/-}$ versus *H3f4*$^{wt/wt}$ elSts (FDR ≤ 0.05) (y axis). Genes up- and downregulated in *H3f4*$^{H3.1/-}$ elSts are highlighted in red and blue, respectively. DEGs are separated into nine groups, based on the |log2FC| = 1 cutoff in both contrasts (dashed lines).

d-e, Boxplots showing absolute quantification of exonic and intronic gene expression in groups 1-9 defined in panel d, comparing *H3f4*$^{H3.1/-}$ to *H3f4*$^{wt/wt}$ cells. Paired t-tests were performed between indicated genotypes: * P < 0.05, ** P ≤ 0.01, *** P ≤ 0.001, **** P ≤ 0.0001.

f, GO term search (GO: Biological Process) for genes in groups 1, 4, 6 and 9 (panel c). A one-sided over representation hypergeometric test was used, followed by Benjamini–Hochberg correction for multiple testing.

g, Boxplots displaying ChIP-seq enrichments of different chromatin signatures (H3.4^{MYC}, H3.3, H3K4me3 and H3K27me3 at TSS ±1 kb) in wildtype rSts for groups 1-9 as defined in panel d.

h, Top: heatmap showing enrichments and adjusted p-values of transcription factor (TF) motifs ($\log_2$) among up- and downregulated genes in *H3f4*$^{H3.1/-}$ elSt cells. Bottom: expression ($\log_2$(RPKM)) of corresponding TF genes in *H3f4*$^{wt/wt}$ and of differential expression (log2FC) in 3 substitution genotypes of ScLZ, ScPD, rSt and elSt cells. Color annotation shows whether the corresponding TF motif was found among the up- or downregulated genes in *H3f4*$^{H3.1/-}$ elSt cells.

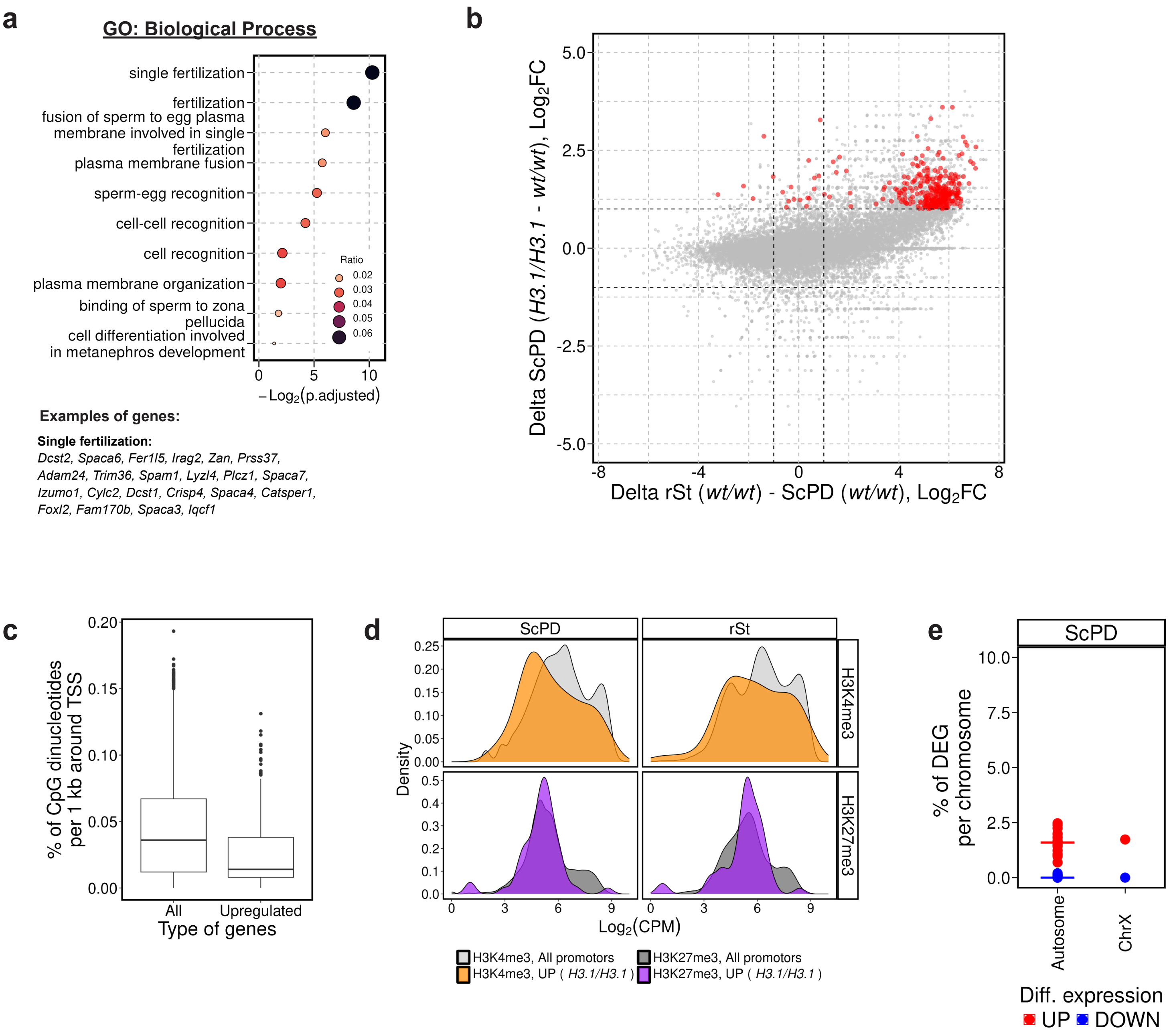
a
GO: Biological Process
single fertilization
fertilization
fusion of sperm to egg plasma membrane involved in single fertilization
plasma membrane fusion
sperm-egg recognition
cell-cell recognition
cell recognition
plasma membrane organization
binding of sperm to zona pellucida
cell differentiation involved in metanephros development
Ratio
0.02
0.03
0.04
0.05
0.06
0
5
10
−Log2(p.adjusted)
Examples of genes:
Single fertilization:
Dcst2, Spaca6, Fer1l5, Irag2, Zan, Prss37, Adam24, Trim36, Spam1, Lyzl4, Plcz1, Spaca7, Izumo1, Cylc2, Dcst1, Crisp4, Spaca4, Catsper1, Foxl2, Fam170b, Spaca3, Iqcf1
b
Delta ScPD (H3.1/H3.1 - wt/wt), Log2FC
5.0
2.5
0.0
-2.5
-5.0
-8
-4
0
4
8
Delta rSt (wt/wt) - ScPD (wt/wt), Log2FC
c
% of CpG dinucleotides per 1 kb around TSS
0.20
0.15
0.10
0.05
0.00
All
Upregulated
Type of genes
d
ScPD
rSt
H3K4me3
H3K27me3
Density
0.25
0.20
0.15
0.10
0.05
0.00
0.5
0.4
0.3
0.2
0.1
0.0
0
3
6
9
Log2(CPM)
H3K4me3, All promotors
H3K4me3, UP (H3.1/H3.1)
H3K27me3, All promotors
H3K27me3, UP (H3.1/H3.1)
e
ScPD
% of DEG per chromosome
10.0
7.5
5.0
2.5
0.0
Autosome
ChrX
Diff. expression
UP
DOWN

Supplementary Figure 22. Gene ontology and chromatin features of genes differentially expressed in ScPD spermatocytes of *H3f4*$^{H3.1/H3.1}$ animals

a, GO term analysis (GO: Biological process) of genes upregulated in *H3f4*$^{H3.1/H3.1}$ versus *H3f4*$^{wt/wt}$ ScPD spermatocytes . Examples of genes within selected terms are displayed below. A one-sided over representation hypergeometric test was used, followed by Benjamini–Hochberg correction for multiple testing.

b, Scatter plot showing differential expression (log2FC) of genes in *H3f4*$^{wt/wt}$ rSts versus *H3f4*$^{wt/wt}$ ScPD (FDR ≤ 0.05) (x axis) compared to differential expression (log2FC) in *H3f4*$^{H3.1/H3.1}$ versus *H3f4*$^{wt/wt}$ ScPD (FDR ≤ 0.05) (y axis). Genes upregulated in *H3f4*$^{H3.1/3.1}$ SCPD are highlighted in red (logFC ≥ 1, FDR ≤ 0.05).

c, Density plots showing the distribution of H3K4me3 and H3K27me3 enrichments at TSS (±1 kb) for promoters of genes upregulated in *H3f4*$^{H3.1/H3.1}$ ScPD (colored fill) and for promoters of all genes (grey fill), as measured in indicated wildtype cell types.

d, Dot plot showing percentages of up- and downregulated genes for each chromosome in *H3f4*$^{H3.1/H3.1}$ ScPD cells. The bars represent the mean values for the selected DEGs. Each dot has been derived from the edgeR estimation of DEGs (panel d) based on 3 biological replicates (n=3) for *H3f4*$^{wt/wt}$ and four biological replicates (n=4) for *H3f4*$^{H3.1/H3.1}$ animals.

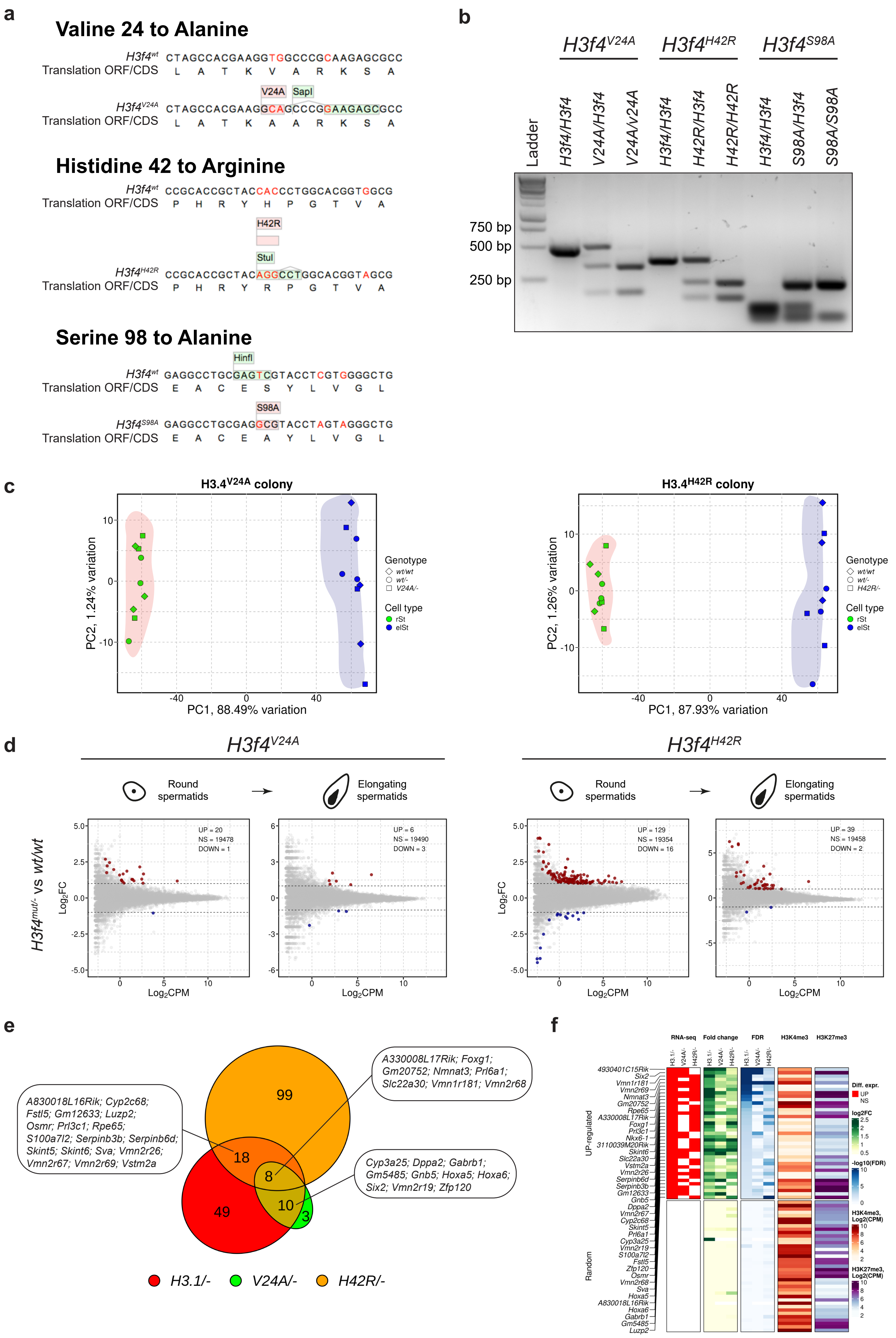

a
Valine 24 to Alanine
H3f4wt CTAGCCACGAAGGTGGCCCGCAAGAGCGCC
Translation ORF/CDS L A T K V A R K S A
V24A SapI
H3f4V24A CTAGCCACGAAGGCAGCCCGGAAGAGCGCC
Translation ORF/CDS L A T K A A R K S A
Histidine 42 to Arginine
H3f4wt CCGCACCGCTACCACCCTGGCACGGTGGCG
Translation ORF/CDS P H R Y H P G T V A
H42R
StuI
H3f4H42R CCGCACCGCTACAGGCCTGGCACGGTAGCG
Translation ORF/CDS P H R Y R P G T V A
Serine 98 to Alanine
HinfI
H3f4wt GAGGCCTGCGAGTCGTACCTCGTGGGGCTG
Translation ORF/CDS E A C E S Y L V G L
S98A
H3f4S98A GAGGCCTGCGAGGCGTACCTAGTAGGGCTG
Translation ORF/CDS E A C E A Y L V G L
b
H3f4V24A H3f4H42R H3f4S98A
Ladder H3f4/H3f4 V24A/H3f4 V24A/V24A H3f4/H3f4 H42R/H3f4 H42R/H42R H3f4/H3f4 S98A/H3f4 S98A/S98A
750 bp
500 bp
250 bp
c
H3.4V24A colony
PC2, 1.24% variation
PC1, 88.49% variation
Genotype
wt/wt
wt/-
V24A/-
Cell type
rSt
elSt
H3.4H42R colony
PC2, 1.26% variation
PC1, 87.93% variation
Genotype
wt/wt
wt/-
H42R/-
Cell type
rSt
elSt
d
H3f4V24A
H3f4H42R
Round spermatids
Elongating spermatids
H3f4mut/- vs wt/wt
Log2FC
Log2CPM
UP = 20 NS = 19478 DOWN = 1
UP = 6 NS = 19490 DOWN = 3
UP = 129 NS = 19354 DOWN = 16
UP = 39 NS = 19458 DOWN = 2
e
99
18
8
49
10
3
A830018L16Rik; Cyp2c68; Fstl5; Gm12633; Luzp2; Osmr; Prl3c1; Rpe65; S100a7l2; Serpinb3b; Serpinb6d; Skint5; Skint6; Sva; Vmn2r26; Vmn2r67; Vmn2r69; Vstm2a
A330008L17Rik; Foxg1; Gm20752; Nmnat3; Prl6a1; Slc22a30; Vmn1r181; Vmn2r68
Cyp3a25; Dppa2; Gabrb1; Gm5485; Gnb5; Hoxa5; Hoxa6; Six2; Vmn2r19; Zfp120
H3.1/- V24A/- H42R/-
f
RNA-seq Fold change FDR H3K4me3 H3K27me3
H3.1/- V24A/- H42R/-
UP-regulated
Random
4930401C15Rik Six2 Vmn1r181 Vmn2r69 Nmnat3 Gm20752 Rpe65 A330008L17Rik Foxg1 Prl3c1 Nkx6-1 3110039M20Rik Skint6 Slc22a30 Vstm2a Vmn2r26 Serpinb6d Serpinb3b Gm12633 Gnb5 Dppa2 Vmn2r67 Cyp2c68 Skint5 Prl6a1 Cyp3a25 Vmn2r19 S100a7l2 Fstl5 Zfp120 Osmr Vmn2r68 Sva Hoxa5 A830018L16Rik Hoxa6 Gabrb1 Gm5485 Luzp2
Diff. expr. UP NS
log2FC
-log10(FDR)
H3K4me3, Log2(CPM)
H3K27me3, Log2(CPM)

Supplementary Figure 23. Individual contributions of H3.4-specific amino acids to efficiency of spermatogenesis.

a, Schematic representation of *H3f4* point substitution alleles. Variant positions in DNA sequence between wildtype *H3f4* (up) and point mutation alleles (down) are indicated in red. For each mouse model, the DNA sequence of the mutated position within *H3f4* is shown and the translation is shown below. Changes in amino acid sequence are indicated in light red and relevant restriction sites are shown in light green. Changes in DNA sequence that are not annotated are silent mutations that were added to prevent cutting of the sgRNA after successful recombination. Abbreviations: CDS, coding sequence; ORF, open reading frame.

b, Genotyping results of wildtype, heterozygous and homozygous mice from colonies with H3.1-specific point mutations. Fragments were PCR amplified (Supplementary Datas 8 and 9) and digested with the following restriction enzymes: SapI for $H3f4^{V24A}$; StuI for $H3f4^{H42R}$; HinfI for $H3f4^{S98A}$ allele. Fragment sizes after nuclease digestion: SapI: 516 bp ($H3f4^{wt/wt}$), 340 bp + 176 bp ($H3f4^{V24A/V24A}$); StuI: 384 bp + 27 bp (shorter band not shown) ($H3f4^{wt/wt}$), 227 bp + 157 bp + 27 bp (shorter band not shown) ($H3f4^{H42R/H42R}$); HinfI: 125 bp + 107 bp + 76 bp ($H3f4^{wt/wt}$), 230 bp + 76 bp ($H3f4^{S98A/S98A}$). Ladder with indicated size (bp) is shown for comparison on the left.

c, Principal component analysis (PCA) comparing RNA-expression of USCS-annotated genes among FACS-isolated rSt and elSt cells of $H3f4^{wt/wt}$, $H3f4^{wt/-}$, $H3f4^{V24A/-}$ and $H3f4^{H42R/-}$ genotypes.

d, Scatter plots showing differential gene expression (log2FC) in $H3f4^{V24A/-}$ (left) or $H3f4^{H42R/-}$ (right) versus $H3f4^{wt/wt}$ FACS-isolated rSt and elSt cells plotted against gene expression levels in control $H3f4^{wt/wt}$ cells. Numbers of significantly upregulated (red), downregulated (blue) and non-significant (grey) genes are displayed (logFC ≥ 1, FDR ≤ 0.05).

e, Euler diagram showing overlaps in DEGs between rSt cells of three different mutants: $H3f4^{H3.1/-}$ (Fig. 6a), $H3f4^{V24A/-}$ and $H3f4^{H42R/-}$ (Supplementary Fig. 23d).

f, Heatmap showing ChIP-seq enrichments of H3K4me3 and H3K27me3 in control rSt cells at promoters of genes (TSS ±1 kb; log2(CPM)) upregulated in $H3f4^{H3.1/-}$, $H3f4^{V24A/-}$ and $H3f4^{H42R/-}$ rSt cells (Fig. 6a). For reference, a randomly selected set of genes is shown below. The EdgeR results for RNA-seq data (significance, fold-change and FDR) are shown for each gene. Names of genes commonly upregulated in at least two cell populations are indicated on the left. H3K4me3 and H3K27me3 in rSt was obtained from Erkek *et al*[61].
