# Supplementary material for: The eutherian-specific histone H3.4 promotes germ cell development and reproductive fitness
Source: Nat Commun. 2026 Jun 5;17:7212. doi: 10.1038/s41467-026-73954-9 (PMC13396413; doi:10.1038/s41467-026-73954-9)
Supplement: Supplementary file 3 — Description of Additional Supplementary Files [file 41467_2026_73954_MOESM3_ESM.pdf]

**Description of Additional Supplementary Data**

**File Name:** Supplementary Data 1.

**Description:** Mammalian orthologs of human *H3-4.*

The Data contains the results of the search of genes orthologous to human *H3-4* in 109 genome assemblies of placental and marsupial species (ENSEMBL release 112). The first three rows represent the sequences of mouse *H3.1*, *H3.2* and *H3.3* used as references in Fig. 1a. For species with known *H3-4* orthologs, the corresponding coordinates, type and sequence (amino acid and nucleotide) are provided. For species where no *H3-4* orthologs were identified, only the latin name and TaxID are provided. We included only protein-coding *H3-4* orthologs residing in *HIST3* cluster into the alignment of protein sequences (Fig.1a). In column K, arrow heads indicate the annotated strand direction of the respective genes: > 5'→3', < 3'←5'.

**File Name:** Supplementary Data 2.

**Description:** Genomic data of *H3* genes in selected placental and marsupial species.

Genomic data of *H3* genes in seven selected placental (human, mouse, elephant, cow, lemur, rabbit, camel) and four marsupial (Tasmanian Devil, opossum, koala, common wombat) species. For each gene, the ENSEMBL ID, coordinates, strand and symbol (if available) are given.

**File Name:** Supplementary Data 3.

**Description:** Divergence rates of H3 proteins in eutherian species.

Calculation of protein divergence rates of H3 variants per species relative to human. As external references, variation rates of several H2A and H2B variants were obtained from published studies[7,8]. The results of this analysis are displayed in Supplementary Fig. 5c.

**File Name:** Supplementary Data 4.

**Description:** Nonsynonymous and synonymous mutations in *H3* genes in eutherian and marsupial species.

Calculation of the ratios of nonsynonymous over synonymous mutations in *H3* genes. As external references, variation rates of several H2A and H2B variants were obtained from previously published studies[7,8]. Significance of positive selection was evaluated using a likelihood ratio test (LRT) with p-values calculated from the $\chi^2$ distribution. The results of this analysis are displayed in Supplementary Fig. 5d.

**File Name:** Supplementary Data 5.

**Description:** Differential gene expression values of all RNA-seq contrasts used in the study.

Genes that are differentially expressed in all contrasts used in the study: $H3f4^{H3.1/-}$ compared to $H3f4^{wt/wt}$, $H3f4^{H3.1/-}$ compared to $H3f4^{wt/-}$, $H3f4^{wt/-}$ compared to $H3f4^{wt/wt}$, $H3f4^{H3.1/H3.1}$ compared to $H3f4^{wt/wt}$, and $H3f4^{MYC/wt}$ compared to $H3f4^{wt/wt}$. Data on FACS-isolated ScLZ, ScPD, rSt and elSt populations. Statistical testing was performed using edgeR with the following parameters: $|logFC| > 1$, $FDR \leq 0.05$, minCpm = 1 (please see Methods for details). Abbreviations: log(PRKM) - log values of reads per kilobase per million in control, logFC - log values of fold change in a contrast, FDR – false discovery rate, sig – UP, DOWN, NS: non-significant.

**File Name:** Supplementary Data 6.

**Description:** Sequences of sgRNAs used for *H3f4* gene editing.

Sequences of sgRNAs used for generating a knock-out deletion allele of the *H3f4* gene as well as epitope-tagged *H3f4* alleles. Synthesis protocols are described in the Methods section.

**File Name:** Supplementary Data 7.

**Description:** Sequences of homology templates used for *H3f4* gene editing.

Data showing the sequences of homology repair templates, their types and methods used for purification. The corresponding alleles are indicated.

**File Name:** Supplementary Data 8.

**Description:** Sequences of oligonucleotides used for genotyping.

Data showing the names and sequences of oligonucleotides used in the study, as well as purposes of their use.

**File Name:** Supplementary Data 9.

**Description:** Strategies used for genotyping of *H3f4* mutant alleles.

Data showing the PCR protocols used for the genotyping of *H3f4* mutant alleles.

**File Name:** Supplementary Data 10.

**Description:** Antibodies used in the study.

Data showing the primary and secondary antibodies used in this study. The dilutions for each assay are indicated.
