# Supplementary material for: The eutherian-specific histone H3.4 promotes germ cell development and reproductive fitness
Source: Nat Commun. 2026 Jun 5;17:7212. doi: 10.1038/s41467-026-73954-9 (PMC13396413; doi:10.1038/s41467-026-73954-9)
Supplement: Supplementary file 2 — Transparent Peer Review file [file 41467_2026_73954_MOESM2_ESM.pdf]

nature portfolio

Peer Review File

The eutherian-specific histone H3.4 promotes germ cell development and reproductive fitness

Corresponding Author: Professor Antoine Peters

This file contains all reviewer reports in order by version, followed by all author rebuttals in order by version.

Version 0:

Reviewer comments:

Reviewer #1

(Remarks to the Author)
Review Komarov et al., 2025

In this manuscript Komarov and colleagues investigate the origin and function of the histone variant H3.4 in mice. H3.4 is a known testis specific variant of canonical histone H3. So far, H3.4 mutants has been shown impede spermatogenesis in mice and mutation in H3.4 in humans are tied to certain form of azoospermia. Yet, H3.4 specific contributions with respect to other H3s expressed in the testis have not been investigated in detail.
The authors begin their study using homology-based approaches to identify putative H3.4 orthologs in mammals. They confirmed that H3.4 is restricted to placental mammals and find universal linkage to a histone cluster shared between placental and marsupial mammals. Their comparative analysis suggests that H3.4 might have evolved from a H3.2-like histone found within this cluster after the split with marsupial.
For functional assays the authors deploy a number of in vivo mouse models. First, they build tagged lines and find that while most line have dominant negative effects, H3.4 cterm MYC/wt heterozygous animals can sustain spermatogenesis albeit with reduced testis weight. Using IF they show that H3.4 MYC is detectable in differentiating spermatogonia but disappears during spermatid elongation. Mapping MYC tagged H3.4 with MNAse-ChIP on FACS testis populations shows enrichment over TSS decorated by H3K4me3 in spermatogonia and spermatocytes but no enrichment in following stages – mirroring what is observed for H3.3.
Then, they investigate the contribution of three H3.4-specific amino-acids replacing them by those found in H3.1 using targeted CRISPR. While substituted H3.4 animal were fertile, heterozygous animals failed to fully rescue spermatogenesis defects in H3.4 null background (H3.1/-). H3.1/- had impaired meiotic progression and failed to produce elongating spermatids. These phenotypes were accompanied by transcriptional changes suggestive of to delayed gene silencing at the end of spermatogenesis. Through individual substitution, the authors end their study showing that V24 and H42 seem to support most of H3.4 functions.

Overall, this manuscript presents original data, the methods are clear, and the results built from technically sound experiments. As you can appreciate from my summary, this is a dense manuscript with multiple mouse models and diverse experimental approaches, this is a significant effort that should be noted. Some of the genetic intricacies and their associated developmental and molecular phenotypes can be difficult to follow for an un-trained reader and challenging to accurately evaluate for reviewers. However, I have several comments listed below to improve the accuracy and clarity of reporting. With this changes in mind, this work should be of interest to reproductive and chromatin biologists alike.

Major:
- Through the manuscript the authors seem to use the term “isoform” for “paralogs” or “gene families” or “variants”. Isoforms should be produced from the same gene locus but not from different loci. I invite them to review their manuscript and clarify when dealing with distinct genes or families.

- While likely, support for syntenic relationship between marsupial and placental TRIM/Histone cluster remain to be confirmed. To help with unambiguous assignment authors could address the following: 1) how many TRIM genes are located in the vicinity of a histone cluster in these species; 2) how unique is the TRIM histone configuration in these genomes.

- L163 « Finally, these H3 proteins harbor a serine at residue 98 (S98) which is frequently present in eutherian H3.4 orthologs suggesting common origin » - sharing a residue doesn't support a common origin without further phylogenetic analysis (with substitution rates modeling …). The authors have all the sequences to perform such analysis that will help with such claim.
- When comparing H3.4 homologs, the authors suggest that they identified extensive variation (e.g. L175). However, there is no direct support for this claim:
o What is the expected rate of variation in this gene? And how is it extensive?
o Same for substitution rates, what is the actual dN/dS measured? And how is it supporting variation?
o There are multiple pseudogenes identified – what is the loss rate in mammals?

- When comparing expression level by WB, could the authors provide quantification details (e.g. L255; L347) perhaps as a supplement?

- ChIP data and methods:
o Can the authors provide additional details on how MNase ChIP signal was normalized and controlled (any input?)? This is important to avoid unspecific enrichments at promoters which are famously known to be hyperChIPable.
o How was H3.4-MYC ChIP specificity controlled? Maybe an anti-myc ChIP in non-transgenic animals? Such control would help evaluate signal/noise.
o In Figure 3A H3.4 signal appears to move from TSS to surrounding regions in ScLZ and PD. Does this indicate that H3.4 is deposited in these regions?
o A few additional controls on MNase digested chromatin would be appreciated to evaluate their quality (in supplement). Thes include: insert size produced; mapping statistics… ?

- The TSS numbers in each cluster are different between Figure 3 and Sup Figure 6 (they were likely done separately) – can the authors specify how the comparison was made to conclude L294: « Along the developmental trajectory from SgD to rSts, H3.4 became gradually evicted and replaced by H3.3 at TSS of genes in a sequence, histone modification and transcription related manner"

- The temporal aspect of this study relies on separately sorted cells by FACS. Considering for example that H3.4 MYC have altered spermatogenesis, the purity of each population might change, altering comparison of profiling results between genotypes. Could the authors discuss this hypothesis?

- Maybe the authors should consider re-working Figure 6 for clarity. The correlative nature of the analyses performed are hard to follow in the displayed items and writing:
o E.g., sentences such as L462: "H3K4me3 levels are normally higher at genes that either failed to become upregulated in H3f4H3.1/- elongating spermatids" what does this means?
o Wouldn't including H3.1/H3.1 as a control be more appropriate to investigate transcriptional changes associated with H3.1 dosage? Why use wt/wt?

- A few discussion points to revise:
o L500: if Hist3 alteration is found in eutherian why would it be expected that elephant wouldn't have it? Please clarify this statement.
o Conclusions on molecular evolution and selection have little direct support in the present manuscript. Please consider revising for accuracy.
o L514: "exceedingly highly"?
o L525: I see no evidence for "directional" selection, please re-write.
o There is no discussion on dosage effects, and yet most of the phenotypes reported are subject to it? Which chromatin processes might be sensitive to this?
o L537: there no such thing as "ancestral genes" in marsupials.

Minor:
- Which alignment software is used within the msa R package?
- When comparing multiple groups and ANNOVA would be preferred over paired-t test (Figure 4 and 7)
- Figure 2D the first 2 rows shows many additional cells with H3.4MYC signal – can the authors describe what these cells are? Are they c-KIT+?
- L312: what is the evidence for a "replacement"?
- Indicate the statistical tests used in Figure 5b
- L396: there is little evidence supporting this conclusion – Maybe the author could move Figure 5 to the supplement?
- L437-441: any statistical support for these correlations? What is the expected overlap considering the developmental progression defects observed in H3.1/- animals

Reviewer #2

(Remarks to the Author)
This study presents a comprehensive characterization of the evolutionary aspects of the histone variant H3.4 (also known as H3T). The authors have generated several independent mouse lines to determine the functional significance of H3.4. Through meticulous characterization, they identified the roles of critical residues by reverting non-synonymous substitutions

in H3f4 to those present in canonical H3.1 (e.g., H3f4-V24A, H3f4-H42R, H3f4-S98A). Overall, the study is of high quality and provides important insights into the functional roles of H3.4. I support the publication of this study and suggest the following major and minor points to further clarify key conceptual aspects.

Major points
1. An important finding is that H3f4-H3.1/H3.1 (homozygous replacement with H3.1) mice did not exhibit any obvious phenotype in spermatogenesis. However, phenotypes were observed in H3f4-H3.1/- mice (heterozygous for the replacement allele and a deletion allele). This suggests that H3.4 itself is not essential for spermatogenesis, but rather, that histone dosage plays a critical role. Moreover, H3.4 may influence reproductive fitness. This conclusion revises previous findings (e.g., Ueda et al., Cell Reports), which suggested H3.4 is essential for spermatogenesis. The authors should explicitly state this updated interpretation in the manuscript.
2. The phenotype observed in H3f4-H3.1/- mice highlights the importance of histone dosage over H3.4-specific function. It appears that the promoter activity of H3.4 may be sufficient to support spermatogenesis, even when driving expression of H3.1. This conceptual distinction—between the role of the H3.4 protein itself and the necessity of proper histone levels—should be clarified in the manuscript.
3. The RNA-seq comparisons among H3f4-H3.1/-, H3f4-wt/-, and H3f4-wt/wt males are solid. However, I suggest including RNA-seq data from H3f4-H3.1/H3.1 mice compared to wild-type. This would help determine whether observed gene expression changes are due to the absence of H3.4 per se or in part due to a consequence of altered histone dosage.
4. The current evaluation of meiotic double-strand break (DSB) repair is insufficient. If the authors claim that impaired meiotic DSB repair is a primary phenotype, they should assess additional recombination intermediates. The γH2AX signal is not exclusive to DSB repair; it also marks abnormal synapsis. In Figure 5d, the phenotype appears minor, suggesting that DSB repair is likely not significantly impaired. I recommend removing the conclusion that DSB repair is defective and instead describing the subtle alterations in γH2AX distribution. This could be discussed in the context of minor defects in chromosome behavior or meiotic prophase progression. Line 528: I disagree with the statement “enhancing meiotic double strand break repair.” Discussion (lines 542–554): This section is overly speculative. The authors should either remove it or provide stronger evidence to support their conclusions regarding DSB repair.
5. The purity of the isolated ScLZ population is not clearly presented. Lines 313–329 mention increased H3.3 levels in ScLZ and describe some H3.3 incorporation but isolating pure ScLZ cells is known to be technically challenging. The authors should report the purity of the isolated populations. If the purity is low, the conclusions drawn from these data should be appropriately revised.

Minor points
1. H3.4 is also known as H3T. Please clarify this early in the abstract.
2. Line 523: Reference #54 does not mention MSCI and should be corrected.
3. Line 525: The phrase “H3f4 has likely been under evolutionary directional selection” is unclear. Please clarify or rephrase.
4. Line 264: Supplementary Figure S5 can be cited here.
5. Please label each lane in Supplementary Figure 3 for clarity.
6. Labels in Figure 6 are not clear and should be improved for readability.

Reviewer #3

(Remarks to the Author)
In this manuscript entitled “The eutherian-specific histone H3.4 promotes germ cell development and reproductive fitness” by Komarov et al., the authors investigate the evolutionary origin and functional significance of the histone H3.4 variant (also known as H3t) in eutherian mammals. Phylogenetic analyses reveal that H3f4 diverged from the ancestral H3.2 gene prior to the eutherian–marsupial split, with elevated substitution rates in eutherian lineages. Functional experiments using mice expressing H3.4 with reverted amino acid residues (V24A, H42R, S98A) to canonical H3.1 demonstrate that these mutations lead to reduced testis weight, defective meiotic double-strand break repair, pachytene spermatocyte apoptosis, impaired spermatid differentiation, and widespread gene expression dysregulation during spermiogenesis. Additionally, the authors employed a Myc-tagged H3.4 fusion protein to analyze its dynamics during spermatogenesis. This approach revealed that H3.4 undergoes dynamic exchange and incorporation into chromatin, highlighting its active role in chromatin remodeling during germ cell development. Overall, the study provides evidence that H3.4 has undergone adaptive evolution, promoting male reproductive fitness via specialized functions in spermatogenesis.

Although previous studies have reported H3.4 knockout experiments, the in vivo functional significance of specific amino acid substitutions in H3.4 has not yet been elucidated. Therefore, the focus of this study is highly interesting and addresses a notable gap in the field. The data and presentation of results are of exceptionally high quality, with meticulous and thorough explanations that make the findings easy to follow. The discussion is engaging and situates the work in a broader biological and evolutionary context. Overall, the manuscript is of very high quality.

For further improvement, I would like to highlight the following points:

Minor Points

1. Line 189: “including the (Supplementary Fig...” may be a typo.

2. Line 197: H3f4 is a gene name, while H3.3 refers to a protein. Consistency in naming is recommended.

3. Line 318: Please define the abbreviation ‘histone PTM’ at its first occurrence.

4. Line 335: For improved readability, I recommend replacing “four” with “three” and “H3.2 genes” with “canonical H3.1 genes.” Following these changes, the phrase “while leaving H3f4^{C96} intact since this residue is present in H3.1” in line 338 should be removed.

5. Line 390: “SCP3” should be corrected to “SYCP3.”

6. Line 457: “H3H27me3” should be corrected to “H3K27me3.”

Major Points

1. Mechanistic Insight (Lines 555–564):
The proposed mechanism, whereby the H3.4^{V24} residue enhances recognition by PHF1 and PHF19, leading to a reduction of H3K27me3 and consequent upregulation of target genes in H3.4$^{H3.1}$, is conceptually convincing. However, it remains unclear whether PHF1 and PHF19 are actually recruited to these upregulated gene loci during the relevant developmental stage. Incorporating public ChIP-seq datasets to demonstrate occupancy would significantly strengthen the discussion and provide direct mechanistic support for this model.

2. Interpretation of Gene Expression Changes (Lines 565–572):
While all observed gene expression abnormalities may be a direct consequence of H3.4$^{H3.1}$ effect, it is also plausible that direct effects are limited to a subset of genes critical for normal spermatid elongation. The majority of expression changes could instead be secondary consequences, resulting from perturbations in spermatid elongation caused by misregulation of these key genes. Considering this distinction would help refine interpretation of the transcriptomic data and the proposed mechanism.

3. Limitations of Tagged Histone Experiments (Lines 613–617):
The results obtained using tagged histones likely reflect a dominant-negative effect of the tag. Although the Myc tag is presumably mild, there is no guarantee that the incorporation and eviction dynamics of tagged histones are identical to those of normal, untagged proteins. This limitation should be explicitly acknowledged in the manuscript. Furthermore, the dynamics observed for H3.4^{MYC} should ideally be validated in future studies using H3.4-specific antibodies to monitor the behavior of the normal protein, thereby confirming that the findings accurately reflect natural H3.4 dynamics in chromatin.

Version 1:

Reviewer comments:

Reviewer #1

(Remarks to the Author)
In this revised manuscript Komarov and colleagues have addressed the majority of reviewers comments. There responses are satisfactory leading to a much improved manuscript. I still have a few minor points that need to be addressed. Assuming these are addressed, I fully support the publication of this manuscript.

Final minor points:
1) The observed MYC cross-reactivity needs to be reported in the main text (results and/or methods)
2) Sup Fig. 3 PhyML tree:
• What substitution model was used?
• Why was the tree rooted on H3.4 sequences? Consider rooting the tree on core H3 or H3s from more distantly related species. This will allow a better analysis of the relative positioning of each protein clade.
• Branch tips: Why mix species names and predicted protein names? Maybe keeping species names would suffice.
3) Figure 4 d-g: it would be better to run a post hoc multiple comparison test following the ANOVA to compare pairs of conditions as presented in the panel. The legend reads that a t-test was used - I am afraid that this is not statistically valid.

Reviewer #2

(Remarks to the Author)
I appreciate the authors’ thorough and thoughtful responses to my previous comments. The additional RNA-seq analyses and the expanded discussion significantly strengthen the manuscript, particularly regarding the interplay between H3f4 sequence and dosage. Overall, the study is of high quality and close to being ready for publication. I have only a minor remaining point, concerning clarity of interpretation rather than additional experiments.

While the revised manuscript substantially improves the discussion of H3f4 dosage effects, I still find the wording around “essentiality” potentially confusing. In particular, the statement that “loss-of-function experiments show that H3f4 is essential for spermatogenesis” could be interpreted as referring to the H3.4 protein itself, whereas the data presented here strongly suggest that the H3f4 locus (expression level and regulatory context) is essential, rather than the H3.4-specific amino acid

sequence per se. I recommend clarifying this distinction in the Results and/or Discussion, to avoid ambiguity and to clearly state how the conclusions refine earlier studies (e.g. Ueda et al.). With this minor clarification, I believe the manuscript provides a rigorous and nuanced contribution to our understanding of histone variant evolution and function in spermatogenesis, and I support its publication.

Open Access This Peer Review File is licensed under a Creative Commons Attribution 4.0 International License, which permits use, sharing, adaptation, distribution and reproduction in any medium or format, as long as you give appropriate credit to the original author(s) and the source, provide a link to the Creative Commons license, and indicate if changes were made.
In cases where reviewers are anonymous, credit should be given to 'Anonymous Referee' and the source.
The images or other third party material in this Peer Review File are included in the article's Creative Commons license, unless indicated otherwise in a credit line to the material. If material is not included in the article's Creative Commons license and your intended use is not permitted by statutory regulation or exceeds the permitted use, you will need to obtain permission directly from the copyright holder.
To view a copy of this license, visit https://creativecommons.org/licenses/by/4.0/

RESPONSE TO REVIEWERS' COMMENTS

Reviewer #1 (Remarks to the Author):

Review Komarov et al., 2025

In this manuscript Komarov and colleagues investigate the origin and function of the histone variant H3.4 in mice. H3.4 is a known testis specific variant of canonical histone H3. So far, H3.4 mutants has been shown impede spermatogenesis in mice and mutation in H3.4 in humans are tied to certain form of azoospermia. Yet, H3.4 specific contributions with respect to other H3s expressed in the testis have not been investigated in detail.

The authors begin their study using homology-based approaches to identify putative H3.4 orthologs in mammals. They confirmed that H3.4 is restricted to placental mammals and find universal linkage to a histone cluster shared between placental and marsupial mammals. Their comparative analysis suggests that H3.4 might have evolved from a H3.2-like histone found within this cluster after the split with marsupial.

For functional assays the authors deploy a number of in vivo mouse models. First, they build tagged lines and find that while most lines have dominant negative effects, H3.4 cterm MYC/wt heterozygous animals can sustain spermatogenesis albeit with reduced testis weight. Using IF they show that H3.4 MYC is detectable in differentiating spermatogonia but disappears during spermatid elongation. Mapping MYC tagged H3.4 with MNAse-ChIP on FACS testis populations shows enrichment over TSS decorated by H3K4me3 in spermatogonia and spermatocytes but no enrichment in following stages – mirroring what is observed for H3.3. Then, they investigate the contribution of three H3.4-specific amino-acids replacing them by those found in H3.1 using targeted CRISPR. While substituted H3.4 animal were fertile, heterozygous animals failed to fully rescue spermatogenesis defects in H3.4 null background (H3.1/-). H3.1/- had impaired meiotic progression and failed to produce elongating spermatids. These phenotypes were accompanied by transcriptional changes suggestive of to delayed gene silencing at the end of spermatogenesis. Through individual substitution, the authors end their study showing that V24 and H42 seem to support most of H3.4 functions.

Overall, this manuscript presents original data, the methods are clear, and the results built from technically sound experiments. As you can appreciate from my summary, this is a dense manuscript with multiple mouse models and diverse experimental approaches, this is a significant effort that should be noted. Some of the genetic intricacies and their associated developmental and molecular phenotypes can be difficult to follow for an un-trained reader and challenging to accurately evaluate for reviewers. However, I have several comments listed below to improve the accuracy and clarity of reporting. With this changes in mind, this work should be of interest to reproductive and chromatin biologists alike.

Reply: We thank the reviewer for the positive appreciation of our manuscript and the constructive feedback provided. Addressing them helped us improve the paper substantially.

Major:
1. - Through the manuscript the authors seem to use the term “isoform” for “paralogs” or “gene families” or “variants”. Isoforms should be produced from the same gene locus but not from different loci. I invite them to review their manuscript and clarify when dealing with distinct genes or families.

Reply: We thank the reviewer for this important comment. We edited the text accordingly and generally replaced “isoforms” with “variants”.

2. - While likely, support for syntenic relationship between marsupial and placental TRIM/Histone cluster remain to be confirmed. To help with unambiguous assignment authors could address the following: 1) how many TRIM genes are located in the vicinity of a histone cluster in these species; 2) how unique is the TRIM histone configuration in these genomes.

Reply: The conservation of a *HIST3* cluster in 9 placental species (human, chimp, macaque, dog, cat, pig, horse, cow and mouse) was previously reported by Seal *et al.* (2022). We extended the analysis to 104 genomes of eutherian species (ENSEMBL Release 112). In 89 of them, we identified a sequence orthologous to *H3-4*, either as a functional gene or as a pseudogene. These *H3-4* orthologs generally

locate adjacent to one *H2A* and two *H2B* genes and to one or two *TRIM* genes (*TRIM17* and *TRIM11*), thereby constituting together orthologous *HIST3* clusters.

In the remaining species, due to poor genomic assemblies, we could not identify either orthologous *H3-4* sequences (shrew, hyrax, megabat, alpaca and American beaver), or neighboring histone genes next to *TRIM*/*H3-4* group (pika and American bison), or *TRIM* genes next to *H3-4* (sloth, lesser hedgehog, tenrec and hedgehog). In four species (blue whale, Bolivian squirrel monkey, kangaroo rat and microbat), despite having better assemblies, we were unable to identify an *H3-4* ortholog, either within the *HIST3* cluster or elsewhere.

In all cases when either (a) *TRIM* gene(s) was/were identified next to an orthologous *H3-4* sequence, they were in the same orientation as *H3-4* and downstream of it. Further, the *TRIM-HIST3* cluster is flanked by the *OBSCN* gene on one side and by *RNF187* on the other side. We would this in most assemblies. These data strongly support the use of *TRIM11*/*17* as a genomic anchor to search for sequences orthologous to *H3-4* in marsupial species. We added the configuration of *H3-4* and neighboring *TRIM* genes for all genomes into Supplementary Fig. 1b and genomic location and their sequences in Supplementary Table 1.

In marsupials, opossum has the best annotated genome. Using TBLASTN search against human H3.4, we identified 15 hits in opossum that matched predicted *H3* genes (Supplementary Table 2 and Supplementary Fig. 4). They correspond to 10 sequences found in three large histone clusters and to five standalone genes that are most similar to *H3.3* and *CENP-A*. Among the clustered *H3* genes, only one (*ENSMODG00000038443*) was found to be next to *TRIM11*, positioned in the same orientation. Similar situations were observed in other marsupial species (Supplementary Figure 5). In Tasmanian devil, Koala and in Common wombat, the predicted *H3-4* orthologs also locate next to *TRIM11*. Only in Wallaby, we were unable to identify the link to *TRIM11*, due to poor genomic assembly.

We have prepared a Supplementary Fig. 4 to show the composition of histone clusters in opossum that contain *H3* genes and the neighbourhood of standalone *H3* genes. In Supplementary Fig. 5, we show the location of putative *H3-4* orthologs in other marsupial species. Supplementary Fig. 6 is a cartoon that shows a graphical summary of marsupial histone gene clusters that contain the putative *H3-4* orthologs, the composition of eutherian *HIST3* and *HIST1* clusters and the composition of a proto-cluster that is ancestral to those in marsupials and eutherians.

3. - L163 « Finally, these H3 proteins harbor a serine at residue 98 (S98) which is frequently present in eutherian H3.4 orthologs suggesting common origin » - sharing a residue doesn't support a common origin without further phylogenetic analysis (with substitution rates modeling …). The authors have all the sequences to perform such analysis that will help with such claim.

Reply: We appreciate this argument. Indeed, in our analysis, we use the linkage of marsupial *H3* genes to *TRIM11* to argue that they are orthologous to placental *H3-4*. In four species (Opossum, Tasmanian devil, Koala and Common wombat), we found such candidates that are H3.2-encoding genes. However, we noticed that these genes also encode an S98 – a residue that is found in ~half of placental H3.4. In Opossum, Tasmanian devil and Koala, no other *H3* gene encodes an S98. Hence, their conserved location and a shared S98 could suggest their common origin. In Wallaby, due to poor assembly, we were unable to find the connection to *TRIM11* but have also identified an *H3.2S98* gene (ENSMEUG00000005042), which could be orthologous to placental *H3-4*. In Common wombat, however, we found an additional *H3.2S98* gene (ENSVURG00010005173) that is located in a large histone cluster, but no *TRIM* gene was found nearby. Most probably, there was a sequencing gap between these two *H3.2S98* genes (Supplementary Fig. 5 and 6). We do not know if such genes emerged due to gene duplication of a *TRIM*-linked *H3.2* gene or it was a spontaneous appearance of S98. Hence, we agree that it can be ambiguous to claim that sharing S98 indicates the common origin of these genes.

To resolve the ambiguity, we investigated phylogenetic relationships between all protein-coding *H3-4* and pseudogenes (Supplementary Table 1) and coding sequences of *H3.1*, *H3.2*, *H3.3* genes in the selected species (Supplementary Table 2). We performed codon alignments of these genes using ClustalW algorithm and then performed maximum likelihood phylogenetic analysis using PhyML tool (Guindon and Gascuel 2003; Guindon *et al.,* 2010). The results of this analysis are in Supplementary Fig. 3a. All H3.3-encoding genes form a separate clade, branching from *H3.1*, *H3.2* and *H3-4* genes. The placental *H3-4* genes and pseudogenes form a separate clade that does not include other genes

(the only exceptions are the *H3.1* paralogs from rabbit). The putative *H3-4* orthologs in marsupials identified using *TRIM* as an anchor form a separate clade among the *H3.2* genes. Interestingly, within this clade, we identified two additional Tasmainan devil *H3.2* genes that do not encode S98 and are also part of a histone cluster that is next to *BTN2A2* and *BTN1A1* genes that are found in the large *TRIM11*-containing cluster in Opossum. In Wombat, the *H3.2S98* gene that is not linked to *TRIM* also belongs to this clade and is also next to *BTN2A2* and *BTN1A1*. At the same time, in wallaby, neither the *H3.2S98* gene, nor any other *H3* genes do not have a sufficient degree of phylogenetic relationship with this clade. Hence, our analysis supports the reviewer's argument that sharing an S98 residue does not necessarily support the common origin of candidate genes.

In summary, we identified that *TRIM*-linked *H3.2* genes from different marsupials form a separate clade that also includes three other *H3.2* genes which could have emerged due to duplication of the original *TRIM*-linked *H3.2* gene. These genes could be orthologous to placental *H3-4*. Considering high separation of *H3-4* and their presumed marsupial orthologs, we hypothesize that they might have emerged from a common ancestor and, after separation of marsupial and placental lineages have evolved independently. Hence, their common origin should be treated with caution.

While doing this analysis, we performed a more thorough inspection of the marsupial *TRIM11*-linked histone clusters. In Opossum and Koala, we noticed that this cluster is flanked by *ZFP496* and *ZFP293* on one side and *BTNA2* and *BTN1A1* on the other. In Wombat, we identified two large histone clusters. One is flanked by *ZFP* genes and another is flanked by *BTN* genes. Both clusters were located at the edge of primary assemblies and most probably are a part of one cluster that is separated by a sequencing gap. In Tasmanian devil, we identified two smaller clusters on different chromosomes that could have emerged due to a breakage of the ancestral large cluster. In eutherians, *BTNA2* and *BTN1A1* are located next to *HIST1* – a large cluster or replication-dependent histone genes. These new data argue for the presence of a large ancestral cluster that underwent several independent fractionations during evolution and *HIST1* and *HIST3* are likely to be its derivatives in eutherians.

4. - When comparing H3.4 homologs, the authors suggest that they identified extensive variation (e.g. L175). However, there is no direct support for this claim:

Reply: Thank you for the next two comments below. We have calculated the variation of H3 variants at the protein level and the *dN/dS* rates of their genes. We included data for different H2A and H2B variants calculated in previous studies (Molaro et al. 2018 and Raman et al. 2022) as references for comparison.

4a. o What is the expected rate of variation in this gene? And how is it extensive?

Reply: We calculated the rate of variation of H3 variants (H3.1, H3.2, H3.3 and H3.4) as the percentage of amino acid identity as a function of evolutionary divergence. The rate of variation was slightly higher than that of replication-coupled H3.1 and H3.2. However, compared to specialized H2A and H2B variants (H2A.P, H2A.L, H2A.B and H2B.W), the rate of H3.4 protein variation is much lower. The results of this analysis are in Supplementary Fig. 3c and Supplementary Table 3.

4b. o Same for substitution rates, what is the actual dN/dS measured? And how is it supporting variation?

Reply: We calculated dN/dS (ω) rates using the CODEML tool from the PAML package (Yang 2007). We used model 0 (assuming similar evolutionary rates for all sites in all branches). The ω values were extremely low for *H3.1* (0.0002) and *H3.2* genes (0.0011 for marsupials and 0.0005 for eutherians), and slightly higher for *H3.3* genes (0.0277). For protein-coding *H3-4* genes, ω was comparable to that of *H3.3* (0.0248). This is in striking contrast to pseudogenized *H3-4* orthologs (0.46) and several specialized H2A and H2B variants (0.24, 0.49, 0,6 and 0.63 for *H2A.P*, *H2A.L*, *H2A.B* and *H2B.W* genes, respectively). This indicates strong purifying selection required to maintain the sequence of H3, which is slightly lower than for *H3.1* and *H3.2* genes. Finally, the likelihood ratio test (LRT) analysis comparing the results of model M0 and model M0 with fixed ω = 1 strongly rejected the possibility of neutral evolution of *H3-4* and other *H3* genes. We also tested model M8 to identify possible sites that could have ω values higher than 1, but were unable to detect those using the Bayes empirical Bayes (BEB) method.

These results are not surprising – contrary to certain H2A and H2B variants which demonstrated high variation at certain positions (e.g. residues 5, 34, 97, 98 and 114 for H2A.P, residue 38 for H2A.B, Molaro et al. 2018), the amino acid variation of H3.4 at certain sites represents the fixation of a given residue in

a given clade (e.g. H42 in muroids or M111 in hominids), while in other species it remains unchanged. The molecular roles of such polymorphisms are also known, for example H42 (Ueda et al. 2017) and M71 and M111 (Tachiwana et al. 2010) were shown to promote more open nucleosome configuration *in vitro*, compared to their somatic counterparts. In line with these data, we provide physiological evidence for the requirement to keep H42 and V24 in mouse.
Overall, our data shows that (1) H3.4 is slightly more variable than H3.1 and H3.2 but is still under strong purifying selection; (2) fixation of certain residues in given clades, possibly in adaptation to physiological and molecular roles. The results of this analysis are in Supplementary Fig. 3d and Supplementary Table 4.

4c. o There are multiple pseudogenes identified – what is the loss rate in mammals?

Reply: We define an orthologous *H3-4* sequence as a pseudogene if it was identified within the *HIST3* cluster, along with a linkage to *TRIM11/17* gene and when this sequence is not predicted to produce a functional protein due truncations and/or frameshift mutations.

Among the 104 eutherian genomes analyzed, 28 (26.9%) of them had *H3-4* as a pseudogene. Those include New World monkeys (2 species), gorilla, Crab-eating macaque, 5 rodents, 5 *Felidae* species, 7 *Canidae* genomes (including 6 different genomic assemblies of *Canis lupus familiaris* of different breeds), four *Artiodactyla* species, sea lion and polar bear.

In 12 genomes (11.5%), we were unable to identify any *H3-4* orthologs either within the orthologous *HIST3* cluster or elsewhere, which could be either due to poor genomic assemblies or because it was indeed lost during evolution.

Unfortunately, there are only four genomes of New World monkeys but it is remarkable that in all of them, the *H3-4* sequence was either as a pseudogene or was not identified at all. Further studies could be interesting to reveal if this loss could be associated with evolutionary history of this primate group. We have added more examples of pseudogenes in Supplementary Fig. 3b. For all genomes that we have analyzed, the information about the orthologous *HIST3* loci is in Supplementary Fig. 1b.

5. - When comparing expression level by WB, could the authors provide quantification details (e.g. L255; L347) perhaps as a supplement?

Reply: We have added the quantification of the western blot data (Supplementary Fig. 8d and Fig. 4c) and added the panels showing the results as Supplementary Fig. 8e and Supplementary Fig. 15c, respectively. For each plot we performed statistical testing that is indicated in legends.

6. - ChIP data and methods:

6a. o Can the authors provide additional details on how MNase ChIP signal was normalized and controlled (any input?)? This is important to avoid unspecific enrichments at promoters which are famously known to be hyperChIPable.

Reply: A detailed description of ChIP normalization is provided in the materials and methods section. In brief, we outline the most important details of normalization here:

Prior to normalization, we have removed blacklisted regions (ENCODE annotation, Amemiya *et al.* 2019) from the downstream analysis. We also excluded some clustered genes, such as keratin, cytochrome and vomeronasal receptor genes, as they tend to have low mappability rates, as well as genes with poor annotation, such as RIKEN genes and gene models (Gm*).

For normalization, we calculated the signal at regions that surround TSS of UCSC-annotated genes (±25 kb). We then added the counts on non-promotor 50 kb regions that have the lowest and highest (below Q25 and above Q75) raw ChIP signal, to capture the genome-wide dynamic range of ChIP signal at the genome-wide scale. Then we performed loess normalization with normalizeBetweenArrays function from the Limma R package (method = "cyclicloess", cyclic.method = "fast") within the same ChIP experiment (e.g. within H3.4-MYC data on SgU, SgD, ScLZ and ScPD cells). After normalization, we removed non-genic regions and proceeded to downstream analysis.

We did not perform normalization using input and did not perform spike-in.

6b. o How was H3.4-MYC ChIP specificity controlled? Maybe an anti-myc ChIP in non-transgenic animals? Such control would help evaluate signal/noise.

Reply: Thank you, we have included the analyses on input and anti-MYC libraires in non-transgenic (*H3f4*$^{wt/wt}$) animals performed in ScPD and rSt cells into the manuscript (Supplementary Fig. 13g). The data are described in the legend of the figure as follows:

> **Supplementary Fig. 13g:** Heatmap comparing ChIP-signal in input, anti-MYC libraries (in *H3f4*$^{wt/wt}$ and MYC-expressing *H3f4*$^{MYC/wt}$ animals separately) and anti-H3.3 libraries (signal pooled from control *H3f4*$^{wt/wt}$ and MYC-expressing *H3f4*$^{MYC/wt}$ animals). The intensity scale for input and anti-MYC libraries is the same. The order of clusters and genes is the same as in Fig. 3a.
>
> The experiment shows the presence of H3.4^{MYC} in chromatin of ScPD and rSt cells at regions up- and downstream of TSS in a sequence-context specific manner. In cells from transgenic animals we observe the following: At CGI promoter genes, H3.4^{MYC} occupancy is visible in flanking regions surrounding TSS while the signal is reduced at the central CGI regions (see Fig 3a for CGI information). At non-CGI promoter genes, H3.4^{MYC} occupancy is largely uniform across the regions except for genes at which nucleosome turnover is observed. Finally, H3.4^{MYC} occupancy is absent at X-linked genic regions indicative of MSCI-related nucleosome replacement of H3.4 by H3.3. H3.3 occupancy reflects an inverse occupancy pattern as seen for H3.4^{MYC}, with high levels at regions having undergone eviction of H3.4^{MYC}, either at TSS or along the X chromosome. In cells of wildtype control mice, the anti-MYC antibody reveals a non-uniform pattern around TSS of CGI genes and partially along X-linked genic regions, opposite of the patterns observed in transgenic H3.4^{MYC} expressing cells. Since H3.4^{MYC} enrichment does not reflect the presence or absence of the DNA-sequence motif of the endogenous MYC protein at promoter regions, it may point to some cross-reactivity with other epitopes. Together, given the inverse ChIP-signal patterns of H3.4^{MYC} and H3.3 proteins, we consider the H3.4^{MYC} signal as representing genuine H3.4^{MYC} occupancy in transgenic animals.

We further calculated the ChIP signal (log2CPM) on 10 kb non-overlapping tiles genome-wide and plotted it against GC percentage (Rebuttal Fig. 1). For input libraries, we observed a uniform distribution of reads on autosomes and the X chromosome irrespective of GC content indicating absence of technical sequence biases.

In anti-MYC libraries generated from transgenic mice, we measured a similar uniform distribution of reads on autosomes while read counts were reduced for X-linked tiles. The opposite effect was seen for the anti-H3.3 libraries. The X-chromosome effects reflect MSCI, where H3.3 replaces H3.4 on sex chromosomes, as reported before for pachytene spermatocytes.

In anti-MYC libraries performed on non-transgenic animals, we observed only slightly different signal distributions for autosomes and the X chromosome, with read counts on chrX being partially overrepresented, particularly in round spermatids.

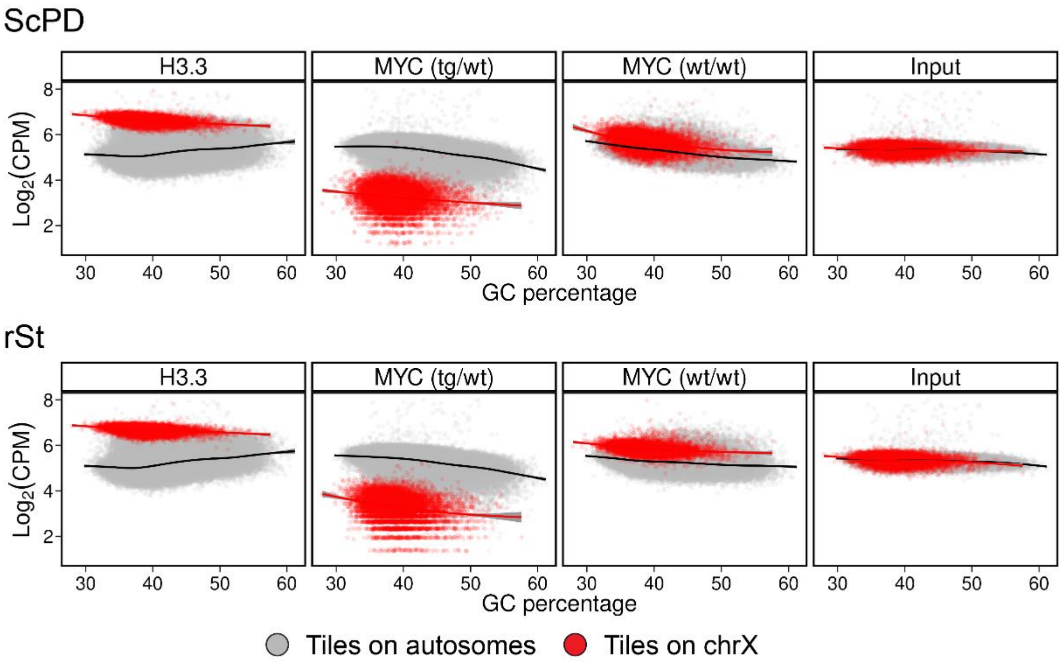

Rebuttal Fig. 1: Distribution of reads counts (Log2(CPM) along 10 kb tiles at autosomes and X chromosome as a function of the GC percentage of respective tiles for the different ChIP-seq samples in ScPD and rSt samples. X-linked read counts were normalized according to chromosome copy number.

Finally, we assessed to what extent ChIP-enrichment of the anti-MYC antibody observed in wildtype animals could reflect binding of the endogenous MYC protein, if expressed, to cognate genomic binding sites in the genome. Towards this, we plotted the anti-MYC ChIP enrichment at TSS +/- 1 kb regions as a function of the GC percentage for the 9 different TSS clusters as shown in Supplementary Fig. 13g and indicated in red which regions contain a MYC motif as represented in the JASPAR2020 database (Rebuttal Fig. 2).

From these analyses, it is clear that regions containing a MYC motif do not show more enrichment compared to regions lacking such motif. Hence, we interpret that the anti-MYC enrichment observed at TSS in control animals, shown in Supplementary Fig. 13g, reflects cross-reactivity to unknown chromatin epitopes.

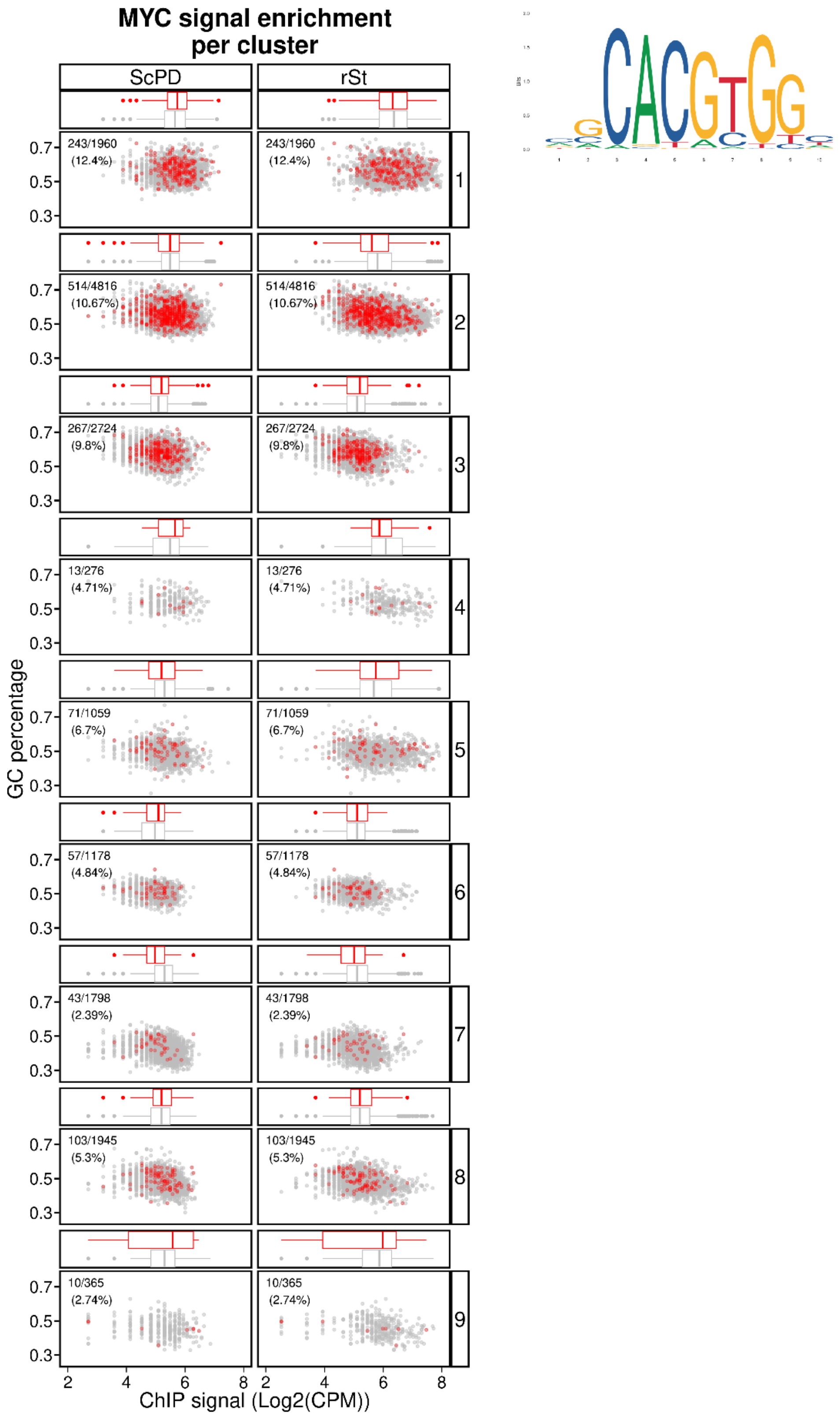

Rebuttal Fig. 2: Distribution of reads counts (Log2(CPM) at different TSS (+/- 1 kb) gene clusters (as shown in Supplementary Fig. 13g) as a function of the GC percentage for the anti-MYC ChIP-seq samples in ScPD and rSt samples of control animals

.

6c. o In Figure 3A H3.4 signal appears to move from TSS to surrounding regions in ScLZ and PD. Does this indicate that H3.4 is deposited in these regions?

Reply: Our data shows genome-wide deposition of H3.4 during spermatogonial amplification because (1) at this developmental time point, *H3f4* is the most highly expressed *H3* gene, (2) the expression of other *H3* genes is downregulated in SgD cells, compared to SgU and (3) H3.4 protein levels outcompetes H3.1 and H3.2 in SgD and ScLZ samples. Hence, during spermatogonial amplification, prior to meiosis, the genome becomes progressively occupied by H3.4 at the expense of H3.1 and H3.2. During and after meiosis, sites of high H3.4 to H3.3 histone turnover, e.g. at TSS of actively expressed genes and chrX (following MSCI) show depletion of H3.4.

To demonstrate the effect better and visualize the broad regions that surround the TSS, we have re-sized the heatmaps (Fig. 3a and Supplementary Fig. 14a) to TSS +/- 25 kb regions. From here, one could see that flanking regions largely stay unchanged during the SgD – ScLZ – ScPD transitions, while it is only the TSS that gets depleted of H3.4. The effect is most dramatic at the TSS of active genes, which are marked by H3K4me3 and also have high levels of H3.3 (e.g. clusters 1, 2 and 3). Hence, it is not the re-location of H3.4 signal to the regions that surround the TSS but rather the retention of H3.4 outside of the TSS and eviction of H3.4 at TSS.

In addition to the heatmaps, we also generated metaplots showing the average ChIP (log2(CPM)) values at TSS +/-25 kb in each cluster (Fig. 3b and Supplementary Fig. 14b).

6d. o A few additional controls on MNase digested chromatin would be appreciated to evaluate their quality (in supplement). Thes include: insert size produced; mapping statistics… ?

Reply: We have prepared Supplementary Fig. 13 to show the mapping rates of libraries used in the analysis, fragment size distribution (for paired-end experiments).

7. - The TSS numbers in each cluster are different between Figure 3 and Sup Figure 6 (they were likely done separately) – can the authors specify how the comparison was made to conclude L294: « Along the developmental trajectory from SgD to rSts, H3.4 became gradually evicted and replaced by H3.3 at TSS of genes in a sequence, histone modification and transcription related manner".

Reply: Thank you for your suggestion. Accordingly, we have re-plotted Supplementary Fig. 6a (now Supplementary Fig. 14a) according to the clustering made in Fig. 3a. The order of genes in each cluster is also the same as in Fig. 3a. Further, to improve the heatmaps, we have ordered the rows within each cluster according to the levels of RNA expression.

8. - The temporal aspect of this study relies on separately sorted cells by FACS. Considering for example that H3.4 MYC have altered spermatogenesis, the purity of each population might change, altering comparison of profiling results between genotypes. Could the authors discuss this hypothesis?

Reply: To address this question, we have performed FACS purity analysis experiment (Supplementary Fig. 10), comparing *wt/wt* and *MYC/wt* animals. The gating parameters of sorted SgU, SgD, ScLZ and ScPD cells are not different between the genotypes. We also performed quality control experiments of sorted spermatocytes (ScLZ and ScPD) to estimate if there is any contamination with other cell types (Supplementary Fig. 10d, e). To this end, we stained sorted cells with gH2A.X (stains unrepaired DNA double strand breaks at autosomes and the X chromosome), HORMAD1 (protein which labels unsynapsed chromosome axes) and synaptonemal complex protein 3 (SYCP3). In late zygotene spermatocytes, where most gH2A.X is located at sex chromosomes, HORMAD1 still marks the unsynapsed axes, which allows us to distinguish those cells with early pachytenes. Our analysis showed no significant contamination with cells other than the target cell population. The only difference we noticed was slightly less SgD in *MYC/wt* animals, compared to *wt/wt* control (Supplementary Fig. 10c), which could be attributed to cell death (Supplementary Fig. 9).

9. - Maybe the authors should consider re-working Figure 6 for clarity. The correlative nature of the analyses performed are hard to follow in the displayed items and writing:

9a. o E.g., sentences such as L462: “H3K4me3 levels are normally higher at genes that either failed to become upregulated in H3f4H3.1/- elongating spermatids” what does this means?

Reply: Thank you for pointing out this unclear sentence. To increase readability, we have edited as follows:

> “Furthermore, H3K27me3 levels are indifferent among the groups of non-CGI genes (Supplementary Fig 21g) and low compared to CGI-type promoter groups with evident H3K27me3 enrichments (Fig. 6i). Finally, differential H3K4me3 occupancies largely reflect expression level differences among the different groups of non-CGI genes in rSts, with genes up- and downregulated in *H3f4*$^{H3.1/-}$ elongating spermatids being more highly expressed and H3K4me3-marked in rSts compared to genes with unchanged expression (Supplementary Fig 21g).”

9b. o Wouldn’t including H3.1/H3.1 as a control be more appropriate to investigate transcriptional changes associated with H3.1 dosage? Why use wt/wt?

Reply: We performed an additional RNA-seq experiment comparing *H3.1/H3.1* animals with *wt/wt* on FACS-isolated ScLZ, ScPD, rSt and elSt cell populations. In total, we performed edgeR analysis on four comparisons:

(1) *wt/-* vs *wt/wt*

(2) *H3.1/H3.1* vs *wt/wt*

(3) *H3.1/-* vs *wt/wt* (presented in the original Fig. 6a)

(4) *H3.1/-* vs *wt/-*

Solely, the effect of gene dosage (1) is very mild, with only one *Nmnat3* gene upregulated in all cell populations. The effect of H3.1 substitution (2) is mild in all cell types, except for ScPD. Interestingly, these genes are mostly unique to contrast (2) and are associated with germ cell development, as seen from GO terms analysis. Accordingly, these tissue specific genes upregulated in *H3.1/H3.1* ScPD have non-CGI promoters. Since we do not observe statistically significant reduction of cell counts in *H3.1/H3.1* mutants (Fig. 4i), these data suggests that upregulation of these genes does not lead to cell death.

Our data show that it is only the combination of gene dosage and H3.4 to H3.1 substitution that leads to major gene mis-regulation and cell death (contrasts 3 and 4). Contrasts (3) and (4) share most of the upregulated genes. We further refer to our response to points 1 – 3 of Reviewer 2.

10. - A few discussion points to revise:

10a. o L500: if Hist3 alteration is found in eutherian why would it be expected that elephant wouldn’t have it? Please clarify this statement.

Reply: The original sentence was meant to underscore the evolutionary origin of the *H3f4* gene, including in elephant.
To be more concise, we have re-written the first paragraph in the discussion as follows:

> “Through synteny analyses, we identified that among eutherians with analyzable genomes, all contain a small *HIST3* gene cluster harboring the *H3-4* locus and one *H2A* and two *H2B* genes flanked by *OBSCN* and *RNF187* genes (Supplementary Fig. 1b). We observed only minor variations to this configuration, such as inversion of *HIST3* and *RNF187* relative to *OBSCN* and a loss of one *H2B* gene in *Cetacea*. Overall, this configuration is highly conserved, as seen from the genomes of elephant and armadillo, being the eutherian species evolutionarily most distantly related to mouse and human.”

10b. o Conclusions on molecular evolution and selection have little direct support in the present manuscript. Please consider revising for accuracy.

Reply: Thank you for the comments on the evolutionary part of the text. We have revised the discussion and results accordingly and added new data on H3.4 protein variation and the rates of synonymous/nonsynonymous mutations of *H3-4* orthologs. Please find the responses to each comment in separate sections above.

10c. o L514: “exceedingly highly”?

Reply: We have removed the word “exceedingly”.

10d. o L525: I see no evidence for “directional” selection, please re-write.

Reply: Thank you for your comment. As described in previous section, we show evidence for purifying selection of amino acids specific to H3.4 and functionally prove the need to keep them. In accordance with the revised evolutionary part of this study, we re-phrase this section as follows:

> “By generating transgenic animals in which we substituted amino acids that are variant between H3.4 and H3.1, we obtained evidence for purifying selection of the *H3-4* sequence resulting in fixation specific residues.”

10e. o There is no discussion on dosage effects, and yet most of the phenotypes reported are subject to it? Which chromatin processes might be sensitive to this?

Reply: We have revised the discussion and included a more in-depth discussion on the impact of sequence alterations and gene dosage as follows:

> In *H3f4*$^{H3.1/-}$ males, gene expression is only moderately affected during spermatogonial proliferation, meiosis and round spermatid differentiation. We predominantly observed preferential upregulation of genes normally bivalently marked by PRC2-mediated H3K27me3 and H3K4me3. These data are consistent with residue H3.4^{V24} being previously shown to promote recognition of tri-methylated H3.4K27 by PHF1 and PHF19, two accessory components of PRC2 complex, stabilizing its chromatin binding and promoting H3K27me3[44,51,93]. Indeed, we observed a clear overlap among genes upregulated in round spermatids isolated from *H3f4*$^{V24A/-}$ and *H3f4*$^{H3.1/-}$ mice. Many of these genes are normally marked by H3K27me3, supporting an *in vivo* role for H3.4 in promoting PRC2-mediated gene repression.
>
> Remarkedly, in *H3f4*$^{H3.1/H3.1}$ animals, we monitored a precocious upregulation of over 300 genes in ScPD cells that otherwise get upregulated in haploid spermatids. These germ cell specific fertilization-associated genes are different from those upregulated in *H3f4*$^{H3.1/-}$ germ cells. The reasons for such distinct *H3.4* gene dosage related transcriptional responses are unknown. Contrasting *H3.4*$^{H3.1/-}$ males, spermatogenesis in *H3f4*$^{H31./H3.1}$ males is, however, not impaired. Then again, expressing the tagged *H3f4*^{MYC} allele at 20% of total H3.4 protein level affects spermatogenesis. And peculiarly, 83% (280/336) of the genes upregulated in *H3f4*$^{H3.1/H3.1}$ ScPD cells are also upregulated in *H3f4*$^{MYC/+}$ ScPD cells (Supplementary table 5). Our data demonstrate a genetic interaction between sequence and dosage of the *H3f4* gene in regulating different sets of genes in a cell type specific manner. These remarkable findings warrant further mechanistic investigations into the dynamics and function of H3.4-nucleosomes considering the H3.4 protein sequence and level.

10f. o L537: there no such thing as “ancestral genes” in marsupials.

Reply: We have removed this sentence and the argument for the selective pressure to keep S98 residue.

Minor:
11. - Which alignment software is used within the msa R package?

Reply: We run msa() R function with default parameters, which uses ClustalW algorithm. For Figure 1A, the order of sequences was defined by the TimeTree (timetree.org), which represents the evolutionary relationships between species. For all other alignments, we have put more description in the respective section in Methods as follows:

> "To generate Fig. 1a, Supplementary Fig. 2 and 3b, alignments of DNA and protein sequences were performed with the msa R package[87] with the default parameters (method = “ClustalW”, order = “input”). For protein sequences, the first amino acid (methionine) was excluded from the analysis. All other alignments were performed using ClustalW algorithm[88] using the available online tool (https://www.genome.jp/tools-bin/clustalw), or MEGA12[89]."

12. - When comparing multiple groups and ANNOVA would be preferred over paired-t test (Figure 4 and 7)

Reply: For each panel, we added the *p*-values from ANOVA test, in addition to the already existing results of t-test. We also updated the legends accordingly.

13. - Figure 2D the first 2 rows shows many additional cells with H3.4MYC signal – can the authors describe what these cells are? Are they c-KIT+?

Reply: Unfortunately, we cannot co-stain GFRa1 or PLZF with c-KIT because all these antibodies have been raised in the same host species (goat). However, based on nuclear morphology, the bright MYC positive cells are most probably late differentiating spermatogonia or preleptotene spermatocytes. These data are in line with previously reported data by Ueda *et al.* (2017) where they used home-made anti-H3.4 (H3t) antibody co-stained with PLZF/KIT.

14. - L312: what is the evidence for a "replacement"?

Reply: The concept of replacement is based on the observation that local $H3.4^{MYC}$ occupancy levels decrease while those of H3.3 increase over time. Combined with the absence of replication and hence lack of renewal of replication dependent histones like H3.4MYC, H3.1 and H3.2 during meiosis and haploid spermatid differentiation, we interpret the change in occupancy as a replacement of $H3.4^{MYC}$-containing nucleosomes by nucleosomes containing the replication-independent H3.3 protein.

15. - Indicate the statistical tests used in Figure 5b

Reply: We have added the description of the statistical test in the legend as follows:
"For each stage, paired t-test was performed between the indicated genotypes. * $P < 0.05$, ** $P \leq 0.01$, *** $P \leq 0.001$, NS – not significant"

16. - L396: there is little evidence supporting this conclusion – Maybe the author could move Figure 5 to the supplement?

Reply: We have extensively revised the content of Figure 5 and related text in the manuscript. We refer Reviewer 1 to the main text.

17. - L437-441: any statistical support for these correlations? What is the expected overlap considering the developmental progression defects observed in H3.1/- animals

Reply: Here, our aim was to only provide empirical description that the genes that are upregulated in *H3.1/-* elongating spermatids are mostly specific to the preceding cell populations (round spermatids), whereas the downregulated genes are indeed involved in the elongation phase. This is explained by the skewed pattern of the scatters in Fig. 6e and Supplementary Fig. 21c.

Reviewer #2 (Remarks to the Author):

This study presents a comprehensive characterization of the evolutionary aspects of the histone variant H3.4 (also known as H3T). The authors have generated several independent mouse lines to determine the functional significance of H3.4. Through meticulous characterization, they identified the roles of critical residues by reverting non-synonymous substitutions in H3f4 to those present in canonical H3.1 (e.g., H3f4-V24A, H3f4-H42R, H3f4-S98A). Overall, the study is of high quality and provides important insights into the functional roles of H3.4. I support the publication of this study and suggest the following major and minor points to further clarify key conceptual aspects.

We thank reviewer #2 for her/his very positive assessment of our study. We appreciate the constructive feedback that helped us to improve the paper.

Major points
1. An important finding is that H3f4-H3.1/H3.1 (homozygous replacement with H3.1) mice did not exhibit any obvious phenotype in spermatogenesis. However, phenotypes were observed in H3f4-H3.1/- mice (heterozygous for the replacement allele and a deletion allele). This suggests that H3.4 itself is not essential for spermatogenesis, but rather, that histone dosage plays a critical role. Moreover, H3.4 may influence reproductive fitness. This conclusion revises previous findings (e.g., Ueda *et al.*, Cell Reports),

which suggested H3.4 is essential for spermatogenesis. The authors should explicitly state this updated interpretation in the manuscript.

Reply: Please see our response below point 3.

2. The phenotype observed in H3f4-H3.1/- mice highlights the importance of histone dosage over H3.4-specific function. It appears that the promoter activity of H3.4 may be sufficient to support spermatogenesis, even when driving expression of H3.1. This conceptual distinction—between the role of the H3.4 protein itself and the necessity of proper histone levels—should be clarified in the manuscript.

Reply: Please see our response below point 3.

3. The RNA-seq comparisons among H3f4-H3.1/-, H3f4-wt/-, and H3f4-wt/wt males are solid. However, I suggest including RNA-seq data from H3f4-H3.1/H3.1 mice compared to wild-type. This would help determine whether observed gene expression changes are due to the absence of H3.4 per se or in part due to a consequence of altered histone dosage.

Reply: We thank the reviewer for raising the interesting questions under points 1-3 regarding the impact of *H3f4* sequence and dosage variation.

To be able to address them, we performed additional RNA-sequencing experiments on FACS sorted germ cells of *H3f4*$^{H3.1/H3.1}$ and *H3f4*$^{wt/wt}$ animals. Interestingly, specifically in ScPD cells, we observed upregulation of genes normally activated in spermatids, arguing for a premature activation of gene expression that is largely associated with GO terms related to fertilization. CpG density analysis indicate that upregulated genes harbor non-CGI-type promoters, characteristic of typical tissue specific genes. Along these lines, we did not observe any enrichment H3K27me3 in control cells while H3K4me3 levels increased upon differentiation of spermatocytes into spermatids (Supplementary Fig. 22).

We presented these new findings in the results section and discussed them in the discussion of the manuscript as follows (lines 642 – 663):

> In *H3f4*$^{H3.1/-}$ males, gene expression is only moderately affected during spermatogonial proliferation, meiosis and round spermatid differentiation. We predominantly observed preferential upregulation of genes normally bivalently marked by PRC2-mediated H3K27me3 and H3K4me3. These data are consistent with residue H3.4^{V24} being previously shown to promote recognition of tri-methylated H3.4K27 by PHF1 and PHF19, two accessory components of PRC2 complex, stabilizing its chromatin binding and promoting H3K27me3[44,51,82]. Indeed, we observed a clear overlap among genes upregulated in round spermatids isolated from *H3f4*$^{V24A/-}$ and *H3.4*$^{H3.1/-}$ mice. Many of these genes are normally marked by H3K27me3, supporting an *in vivo* role for H3.4 in promoting PRC2-mediated gene repression.
>
> Remarkedly, in *H3f4*$^{H3.1/H3.1}$ animals, we monitored an precocious upregulation of over 300 genes in ScPD cells that otherwise get upregulated in haploid spermatids. These germ cell specific fertilization-associated genes are different from those upregulated in *H3.4*$^{H3.1/-}$ germ cells. The reasons for such distinct *H3.4* gene dosage related transcriptional responses are unknown. Contrasting *H3f4*$^{H3.1/-}$ males, spermatogenesis in *H3f4*$^{H31./H3.1}$ males is, however, not impaired. Then again, expressing the tagged *H3.4*^{MYC} allele at 20% of total H3.4 protein level impacts on spermatogenesis. And peculiarly, 83% (280/336) of the genes upregulated in *H3f4*$^{H3.1/H3.1}$ ScPD cells are also upregulated in *H3f4*$^{MYC/+}$ ScPD cells (Supplementary Table 5). Our data reveal a genetic interaction between sequence and dosage of the *H3f4* gene in regulating different sets of genes in a cell type dependent manner. These remarkable findings warrant further mechanistic investigations into the dynamics and function of H3.4-nucleosomes considering the H3.4 protein sequence and level.

Genetically speaking, the loss-of-function experiments of the *H3f4* locus show that the gene is essential for spermatogenesis (Ueda et al., 2017; this study). Our current study further refines this conclusion in a sense that the sequence composition of *H3f4* as well as it levels matter with respect to the H3.4 function during spermatogenesis.

4. The current evaluation of meiotic double-strand break (DSB) repair is insufficient. If the authors claim that impaired meiotic DSB repair is a primary phenotype, they should assess additional recombination intermediates. The γH2AX signal is not exclusive to DSB repair; it also marks abnormal synapsis. In Figure 5d, the phenotype appears minor, suggesting that DSB repair is likely not significantly impaired. I recommend removing the conclusion that DSB repair is defective and instead describing the subtle alterations in γH2AX distribution. This could be discussed in the context of minor defects in chromosome

behavior or meiotic prophase progression. Line 528: I disagree with the statement "enhancing meiotic double strand break repair." Discussion (lines 542–554): This section is overly speculative. The authors should either remove it or provide stronger evidence to support their conclusions regarding DSB repair.

Reply: We acknowledge the concern of the reviewer. To start approaching this point, we aimed to obtain a more quantitative view on the efficiency by which spermatocytes progress through zygotene and early pachytene in *H3f4*$^{H3.1/-}$ animals versus *H3f4*$^{wt/-}$ animals. By carefully reviewing histological sections stained by clPARP1, we noticed death of spermatocytes at stage XII that are normally transitioning from zygotene to pachytene (Supplementary Fig. 18), suggestive for defects in chromosome synapsis and/or DSB repair. This data is in line with the reduced number of pachytene spermatocytes at stage V while the number of zygotene spermatocytes at stage X was unaltered (Fig. 4i). Quantitative analysis of meiotic spreads shows that early pachytene-like spermatocytes, characterized by (almost) complete autosomal chromosome synapsis and absence of H1t labeling, are more frequently labeled by γH2AX in *H3f4*$^{H3.1/-}$ animals than in *H3f4*$^{wt/-}$ animals. We describe these findings as follows:

> We observed comparable patterns of γH2A.X and SCYP3 in leptotene, zygotene and in mid-pachytene to diakinesis stage spermatocytes of *H3f4*$^{wt/-}$ and *H3f4*$^{H3.1/-}$ mice. However, early pachytene-like spermatocytes, characterized by (almost) complete autosomal chromosome synapsis and absence of H1T labeling, were more frequently labeled by γH2AX in *H3f4*$^{H3.1/-}$ animals than in *H3f4*$^{wt/-}$ animals (Fig. 5c, 5d, Supplementary Fig. 19). The data suggest that not all *H3f4*$^{H3.1/-}$ spermatocytes manage to process meiotic DSBs on autosomes appropriately prior their entry into pachytene stage, consistent with the increased cell death of such early *H3f4*$^{H3.1/-}$ spermatocytes (Fig. 5b, Supplementary Fig. 18). In summary, our data argues that chromatin containing H3.4-nucleosomes facilitates progression through early meiotic prophase more effectively than H3.1-comprising chromatin.

We further consider that a full dissection of the molecular mechanism underlying the observed defects requires an extensive in-depth follow up study which goes beyond the scope of this paper.

5. The purity of the isolated ScLZ population is not clearly presented. Lines 313–329 mention increased H3.3 levels in ScLZ and describe some H3.3 incorporation but isolating pure ScLZ cells is known to be technically challenging. The authors should report the purity of the isolated populations. If the purity is low, the conclusions drawn from these data should be appropriately revised.

Reply: Thank you for raising this comment. For FACS sorting of spermatogonial and meiotic cells, we used a protocol previously described (Fanourgakis *et al.*, 2025; ref 69). The double check the purity of ScLZ and ScPD populations we performed new FACS experiments and analyzed the purity of meiotic cells using immunofluorescence analysis of spread cells as shown in Supplementary Figs. 10d and 10e. These analyses confirmed that the purity of both sorted meiotic populations was above 95%. We refer to the purity of ScLZ cells when discussing the notable early deposition of H3.3 histones into chromatin along the X chromosome prior to the cytologically observable nucleosome eviction of H3.4 in pachytene spermatocytes as discovered by Van der Heijden *et al.*,2007 (ref 46).

Minor points
1. H3.4 is also known as H3T. Please clarify this early in the abstract.

Reply: We have changed the beginning of the abstract as follows:

> "Many genes encoding chromatin proteins are subject to evolutionary selection driving reproductive fitness. In mice and men, the histone H3.4 variant (formerly known as H3t) is essential to spermatogenesis"

2. Line 523: Reference #54 does not mention MSCI and should be corrected.

Reply: Thank you, this was an error. We have removed this reference accordingly.

3. Line 525: The phrase "H3f4 has likely been under evolutionary directional selection" is unclear. Please clarify or rephrase.

Reply: Thank you for your comment. Following your and reviewer 1's comment, we have re-written this phrase as follows:

> "By generating transgenic animals in which we substituted amino acids that are variant between H3.4 and H3.1, we provide evidence for purifying selection of the *H3f4* sequence, resulting in fixation of specific residues".

4. Line 264: Supplementary Figure S5 can be cited here.

Reply: Thank you, we now refer to Supplementary Fig. 5, now 12, in the revised text.

5. Please label each lane in Supplementary Figure 3 for clarity.

Reply: Most probably, this comment refers to panel e. Here, each lane corresponds to the fractions of HPLC separation of histones in the sequential order. We updated the panel with the numbers of fractions accordingly.

6. Labels in Figure 6 are not clear and should be improved for readability.

Reply: We have increased the size of labels in most panels and especially improved the readability of panels h (GO terms) in Fig. 6 and Supplementary Fig. 21f.

Reviewer #3 (Remarks to the Author):

In this manuscript entitled "The eutherian-specific histone H3.4 promotes germ cell development and reproductive fitness" by Komarov et al., the authors investigate the evolutionary origin and functional significance of the histone H3.4 variant (also known as H3t) in eutherian mammals. Phylogenetic analyses reveal that H3f4 diverged from the ancestral H3.2 gene prior to the eutherian–marsupial split, with elevated substitution rates in eutherian lineages. Functional experiments using mice expressing H3.4 with reverted amino acid residues (V24A, H42R, S98A) to canonical H3.1 demonstrate that these mutations lead to reduced testis weight, defective meiotic double-strand break repair, pachytene spermatocyte apoptosis, impaired spermatid differentiation, and widespread gene expression dysregulation during spermiogenesis. Additionally, the authors employed a Myc-tagged H3.4 fusion protein to analyze its dynamics during spermatogenesis. This approach revealed that H3.4 undergoes dynamic exchange and incorporation into chromatin, highlighting its active role in chromatin remodeling during germ cell development. Overall, the study provides evidence that H3.4 has undergone **adaptive** evolution, promoting male reproductive fitness via specialized functions in spermatogenesis

Although previous studies have reported H3.4 knockout experiments, the in vivo functional significance of specific amino acid substitutions in H3.4 has not yet been elucidated. Therefore, the focus of this study is highly interesting and addresses a notable gap in the field. The data and presentation of results are of exceptionally high quality, with meticulous and thorough explanations that make the findings easy to follow. The discussion is engaging and situates the work in a broader biological and evolutionary context. Overall, the manuscript is of very high quality.

We thank also this reviewer for the sincere appreciation of our study and for raising the interesting questions to our study.

For further improvement, I would like to highlight the following points:

Minor Points
1. Line 189: "including the (Supplementary Fig..." may be a typo.

Reply: Thank you for noticing the typo. We have corrected the sentence.

2. Line 197: H3f4 is a gene name, while H3.3 refers to a protein. Consistency in naming is recommended.

Reply: Thank you for this remark. We have carefully checked the paper to consistently use the gene or protein name, when appropriate.

3. Line 318: Please define the abbreviation 'histone PTM' at its first occurrence.

Reply: Thank you, we have incorporated the definition into the text

4. Line 335: For improved readability, I recommend replacing “four” with “three” and “H3.2 genes” with “canonical H3.1 genes.” Following these changes, the phrase “while leaving H3f4^{C96} intact since this residue is present in H3.1” in line 338 should be removed.

Reply: We thank the reviewer for raising this point which we do understand well. Nevertheless, since *H3f4* likely evolved from an ancestral H3.2-encoding gene, we want to convey this notion to the readers of the paper. Hence, we have further tried to improve the readability of the text while retaining this notion.

5. Line 390: “SCP3” should be corrected to “SYCP3.”

Reply: Thank you, we have corrected this abbreviation.

6. Line 457: “H3H27me3” should be corrected to “H3K27me3.”

Reply: Thank you for noticing this mistake. We have corrected it.

Major Points

1. Mechanistic Insight (Lines 555–564):
The proposed mechanism, whereby the H3.4^{V24} residue enhances recognition by PHF1 and PHF19, leading to a reduction of H3K27me3 and consequent upregulation of target genes in H3.4$^{H3.1}$, is conceptually convincing. However, it remains unclear whether PHF1 and PHF19 are actually recruited to these upregulated gene loci during the relevant developmental stage. Incorporating public ChIP-seq datasets to demonstrate occupancy would significantly strengthen the discussion and provide direct mechanistic support for this model.

Reply: We fully share the view of the reviewer. Unfortunately, there are no public ChIP-seq data for PHF1 and PHF19 in germ cells available. Nonetheless, to somehow approach the question, we hypothesized that substitution of H3.4 to H3.1 may reduce the efficacy of PRC2 recruitment to its target genes and hence may reduce local H3K27me3 occupancy, particularly at genes derepressed in *H3.4*$^{H3.1/-}$ germ cells.

To investigate this hypothesis, we performed ChIP-seq experiment for H3K27me3 in ScPD and rSt cells of *H3.4*$^{H3.1/-}$ and *H3.4*$^{wt/wt}$ genotypes. Unfortunately, we failed to identify genes with reduced H3K27me3 occupancy that correlated with upregulated RNA expression in *H3.4*$^{H3.1/-}$ germ cells (Rebuttal Fig. 3). More quantitative approaches will be needed in the future to address this point further.

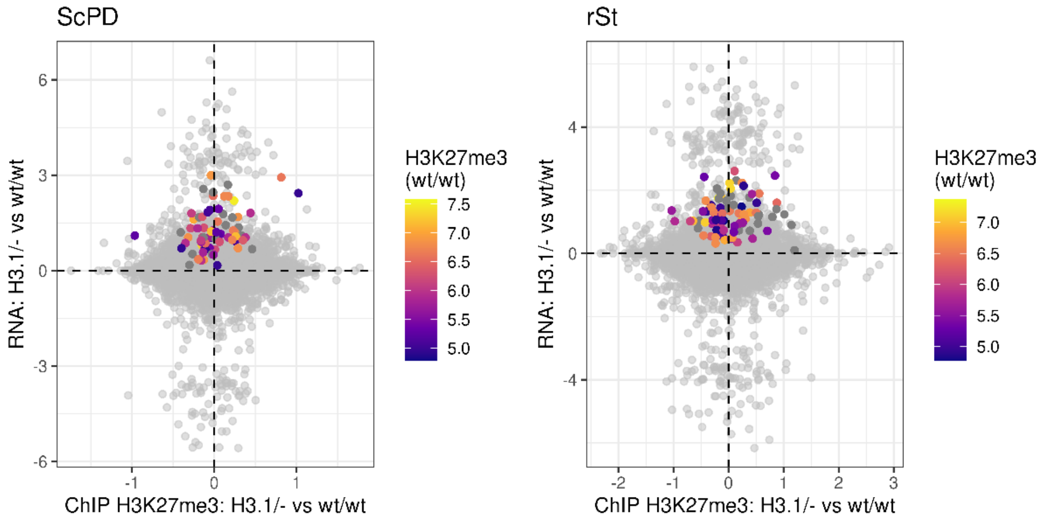

Rebuttal Fig. 3. Scatter plots displaying differences in H3K27me3 occupancy at promotors of UCSC-annotated genes (x-axis) versus RNA levels (y-axis) between *H3.4*$^{H3.1/-}$ and *H3.4*$^{wt/wt}$ genotypes in ScPD (left) and rSt (right) cell types. While grey dots represent all genes, colored dots indicate genes with upregulated RNA levels in *H3.4*$^{H3.1/-}$ mutants. The color scale represents H3K27me3 occupancy (CPM) at TSS (+/- 1 kb) in control samples.

2. Interpretation of Gene Expression Changes (Lines 565–572):
While all observed gene expression abnormalities may be a direct consequence of $H3.4^{H3.1}$ effect, it is also plausible that direct effects are limited to a subset of genes critical for normal spermatid elongation. The majority of expression changes could instead be secondary consequences, resulting from perturbations in spermatid elongation caused by misregulation of these key genes. Considering this distinction would help refine interpretation of the transcriptomic data and the proposed mechanism.

Reply: Thank you for the insightful question. To address this point, we investigated whether there are any DNA binding motifs of known transcription factors (TFs) enriched at promoters of differentially expressed genes. We also reviewed expression levels of possible regulatory TFs. This approach and findings are described as follows in the results section:

> Next to a possible direct impact of altered nucleosome composition, changes in expression of transcription factors (TFs) may also have contributed to the massive transcriptional derailment observed in $H3.4^{H3.1/-}$ elSts. To explore this notion, we first searched for the presence of transcription factor motifs in promoters of aberrantly regulated genes and secondly reviewed transcript levels of corresponding TFs (Supplementary Fig. 21h). Among downregulated genes, we measured significant enrichment of motifs for the immediate early gene response activating TFs FOS and JUN and observed reduced *Jund* and *Fosb* transcript levels specifically in $H3.4^{H3.1/-}$ elSts. These two factors are critical for controlling spermatogenesis (Johnson et al., 1991; Thépot et al., 2000). Likewise, motifs for HMG box TFs including SOX and HMG family members were significantly enriched. While transcript levels of *Hmgb2* and *Sox17* were aberrantly increased, those of *Sox18* and *Sox30* were downregulated particularly in $H3.4^{H3.1/-}$ elSts. Hmgb2 is normally highly expressed in spermatocytes and is essential for spermatogenesis (Ronfani et al. 2001). *Sox30* is required for spermatid differentiation (Feng et al., 2017). An altered balance of such activating and repressive TFs may ultimately suppress spermiogenic genes during spermatid elongation (Horowitz et al., 2005). Among upregulated genes, the motif of the transcriptional repressor Nr6a1 (Gcnf) was enriched and expression of its transcripts reduced, possibly explaining sustained expression of target genes normally downregulated during spermatid elongation (Rajkovic et al., 2010; Sabour et al., 2014). In contrast, expression levels of the *Creb3l4* and *Creb5*, for which motifs were enriched in upregulated target genes, were downregulated, possibly affecting spermiogenesis as well (Adham et al. 2005).

3. Limitations of Tagged Histone Experiments (Lines 613–617):
The results obtained using tagged histones likely reflect a dominant-negative effect of the tag. Although the Myc tag is presumably mild, there is no guarantee that the incorporation and eviction dynamics of tagged histones are identical to those of normal, untagged proteins. This limitation should be explicitly acknowledged in the manuscript. Furthermore, the dynamics observed for $H3.4^{MYC}$ should ideally be validated in future studies using H3.4-specific antibodies to monitor the behavior of the normal protein, thereby confirming that the findings accurately reflect natural H3.4 dynamics in chromatin.

Reply: Thank you for this important comment about the limitations of $H3.4^{MYC}$ model. To better characterize the possible phenotypic effects introduced by the MYC tag, we performed an additional RNA-seq experiment on FACS-isolated ScLZ, ScPD, rSt and elSt populations (Supplementary Fig. 9). We described these findings as follows:

> The reduction in testis weight in $H3f4^{MYC/wt}$ heterozygotes could be attributed to elevated cell death of spermatogonia and spermatocytes, compared to control animals (Supplementary Fig. 9a, b, 10). In $H3f4^{MYC/wt}$ FACS-sorted ScPD spermatocytes, we detected premature upregulation of genes associated with spermatid development while developmental genes were upregulated in elongating spermatids (Supplementary Fig. 9c-e). A substantially higher percentage of X-linked genes was upregulated compared to autosomal genes in ScPD and elSts possibly pointing to a partial derailment of MSCI (Supplementary Fig. 9f). This is surprising given that $H3.4^{MYC}$ appears by immunofluorescence to be evicted and replaced by H3.3 along sex chromosomes during MSCI as reported before[42] (Supplementary Fig. 11). Western blot experiments of FACS-isolated spermatocytes from $H3f4^{MYC/wt}$ mice showed that $H3.4^{MYC}$ was expressed at a level of ~20% of non-tagged H3.1/H3.2/H3.4 proteins (Supplementary Fig. 8d, e). Despite these disturbances of spermatogenesis, we next used the transgenic $H3f4^{MYC/wt}$ mice to investigate the global dynamics of H3.4 deposition, removal and its genomic distribution.

RESPONSE TO REVIEWERS' COMMENTS

Reviewer #1 (Remarks to the Author):

In this revised manuscript Komarov and colleagues have addressed the majority of reviewers comments. There responses are satisfactory leading to a much-improved manuscript. I still have a few minor points that need to be addressed. Assuming these are addressed, I fully support the publication of this manuscript.

Reply: We thank the reviewer for the highly positive evaluation of the manuscript after the first round of revision. Below we provide our responses to the final points.

Final minor points:

1) The observed MYC cross-reactivity needs to be reported in the main text (results and/or methods)

Reply: To clarify the source of cells used in the H3.4^{MYC} ChIP-seq studies, we now added in the main text and legend of Fig.3a a sentence referring to *H3f4*$^{MYC/wt}$ mice as the origin of the cellular material. We further added the following description to the Materials and Method section (lines 1155 – 1161).

> In ScPD and rST cells of wildtype control mice, the anti-MYC antibody revealed a non-uniform pattern around TSS of CGI genes and partially along X-linked genic regions, opposite of the patterns observed in H3.4^{MYC}-expressing *H3f4*$^{MYC/wt}$ transgenic cells (Supplementary Fig. 13g). Since H3.4^{MYC} enrichment in *H3f4*$^{wt/wt}$ mice does not reflect the presence or absence of the DNA-sequence motif of the endogenous MYC protein at promoter regions, it may point to cross-reactivity with other epitopes. We consider the H3.4^{MYC} signal in *H3f4*$^{MYC/wt}$ transgenic animals as representing genuine H3.4 occupancy.

2) Sup Fig. 3 PhyML tree:

• What substitution model was used?

Reply: We described the substitution model in the Methods section of the original manuscript as follows (the sentence related to the model is highlighted in yellow). We now added the description of the rooting on *H3.2* genes obtained from the *Xenopus* genome (highlighted in bold) (lines 762 – 772).

> For maximum likelihood analysis (Supplementary Fig. 3a), we retrieved coding sequences of protein-coding orthologs of *H3-4*, DNA sequences of *H3-4* pseudogenes (regions of homology to functional *H3-4*) from all eutherian species listed in Supplementary Table 1, as well as coding sequences of *H3.1*, *H3.2* and *H3.3* genes from the selected species (Supplementary Table 2). We aligned the sequences and downloaded the output in the Phylip format. Next, we uploaded the alignment file to the PhyML tool[101,102] available online (http://www.atgc-montpellier.fr/phyml/) to build a tree using the HKY85 substitution model with 100 bootstrap replicates[102]. To edit the tree, we used the Interactive Tree of Life tool[103] available online (https://itol.embl.de/). **For rooting, we used coding sequences of *H3.2* genes from *Xenopus tropicalis*, as an outgroup, being an evolutionarily distant species**.

• Why was the tree rooted on H3.4 sequences? Consider rooting the tree on core H3 or H3s from more distantly related species. This will allow a better analysis of the relative positioning of each protein clade.

Reply: Thank you for suggesting this important point. We added the coding sequences of *H3.2* genes from the *X.tropicalis* genome to the analysis. We later rooted the tree on one of these *H3.2* genes. We have updated Supplementary Figure 3a accordingly.

• Branch tips: Why mix species names and predicted protein names? Maybe keeping species names would suffice.

Reply: There is only one *H3-4* unique ortholog (protein-coding or pseudogene) for each species, which is why we indeed use species names. As for the H3.1/2/3-encoding genes, there are multiple gene copies per species, which is why it is more informative to use formal gene names (for mouse and human) and ENSEMBL IDs for the other species. Finally, using species names for *H3-4* genes and ENSEMBL IDs for *H3.1/2/3* genes is also a way to distinguish the variants.

3) Figure 4 d-g: it would be better to run a post hoc multiple comparison test following the ANOVA to compare pairs of conditions as presented in the panel. The legend reads that a t-test was used - I am afraid that this is not statistically valid.

Reply: We thank the reviewer for this note. We performed the ANOVA testing and then the Games–Howell post hoc multiple-comparison test. We changed panels accordingly and legends as follows:

> "Statistical analysis was performed using one-way ANOVA followed by a two-sided Games–Howell post hoc multiple-comparison test to assess pairwise differences between genotypes. Significance levels are based on adjusted p-values from the post hoc test. NS, not significant; * $p < 0.05$; ** $p \leq 0.01$; *** $p \leq 0.001$."

Reviewer #2 (Remarks to the Author):

I appreciate the authors' thorough and thoughtful responses to my previous comments. The additional RNA-seq analyses and the expanded discussion significantly strengthen the manuscript, particularly regarding the interplay between *H3f4* sequence and dosage. Overall, the study is of high quality and close to being ready for publication. I have only a minor remaining point, concerning clarity of interpretation rather than additional experiments.

Reply: We greatly thank the reviewer for the highly positive evaluation of our improvements to the text and data.

While the revised manuscript substantially improves the discussion of *H3f4* dosage effects, I still find the wording around "essentiality" potentially confusing. In particular, the statement that "loss-of-function experiments show that *H3f4* is essential for spermatogenesis" could be interpreted as referring to the H3.4 protein itself, whereas the data presented here strongly suggest that the *H3f4* locus (expression level and regulatory context) is essential, rather than the H3.4-specific amino acid sequence per se. I recommend clarifying this distinction in the Results and/or Discussion, to avoid ambiguity and to clearly state how the conclusions refine earlier studies (e.g. Ueda et al.). With this minor clarification, I believe the manuscript provides

a rigorous and nuanced contribution to our understanding of histone variant evolution and function in spermatogenesis, and I support its publication.

Reply: to accommodate the reviewer's concern we made three changes in the main text of the manuscript as follows:

Abstract: Line 29; we added the wording: "…**the *H3-4 (H3f4)* gene encoding** the histone H3.4 variant…"

Introduction: Line 98: we rephrased as follows: "…**the *H3f4* locus** is essential…"

Discussion: Lines 615 to 633: we added the sentences in bold:

> "**Through the H3.4 to H3.1 reconstitution experiment we show that expression of a H3 protein from the *H3f4* locus is critical to male fertility in mice, thereby refining the notion that the *H3f4* locus is essential for spermatogenesis (Ueda et al., 2017).** Moreover, by generating transgenic animals in which we substituted amino acids that are variant between H3.4 and H3.1, we obtained evidence for purifying selection of the *H3f4* sequence, resulting in fixation of specific residues. Our data show that particularly the combination of $H3.4^{V24}$, $H3.4^{H42}$ and $H3.4^{S98}$ substitutions majorly promotes spermatogenesis, enhancing progression through meiotic prophase, gene regulation and germ cell viability. Considering the positive yet limited impact of single amino acid substitutions on germ cell development, we conclude that the $H3.4^{V24}$ and $H3.4^{H42}$ substitutions synergize in some way in improving spermatogenesis. Surprisingly, the positive cellular impact of H3.4 over H3.1 sequence was noticeable only under reduced gene dosage conditions of RC H3 histones, even though we did not detect differences in total H3 levels by Western blot analyses between homozygous or hemizygous expression conditions of H3.4 or H3.1. Our data reveal that progression through meiotic prophase as well as nuclear elongation and condensation are particularly sensitive to H3.4 specific sequence acquisitions, while plain heterozygosity for H3.4 induced only limited cell death. The impact of $H3.4^{S98A}$ is neutral. **Together, our data show that the expression level and amino acid composition of H3.4 specify its optimal function during spermatogenesis**".
